# Supplementary material for: Reaction hijacking inhibition of Plasmodium falciparum asparagine tRNA synthetase
Source: Nat Commun. 2024 Jan 31;15:937. doi: 10.1038/s41467-024-45224-z (PMC10831071; doi:10.1038/s41467-024-45224-z)
Supplement: Supplementary file 1 — Supplementary Information [file 41467_2024_45224_MOESM1_ESM.pdf]

Supplementary information

Stanley C. Xie, Yinuo Wang, Craig J. Morton, Riley Metcalfe *et al*.
Corresponding authors: Elizabeth Winzeler (ewinzeler@health.ucsd.edu), Michael Griffin (mgriffin@unimelb.edu.au), Matthew Todd (matthew.todd@ucl.ac.uk), Leann Tilley (ltilley@unimelb.edu.au)

The PDF file includes:

Supplementary Figures 1 to 13
Supplementary Tables 1 to 9
Chemistry Materials and Methods
Supplementary References

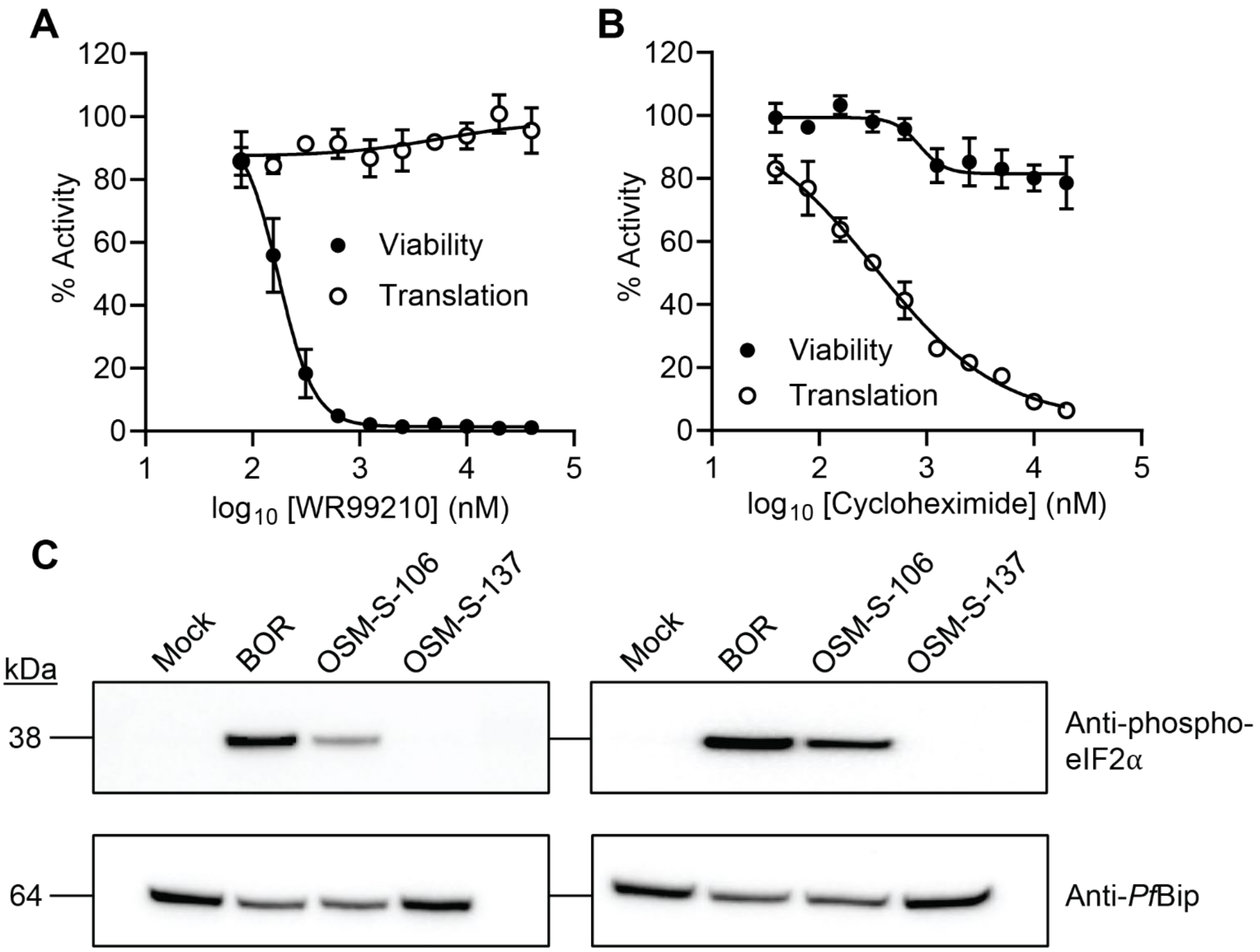

Supplementary Figure 1. Effects of compounds on protein translation inhibition and eIF2α phosphorylation.

(**A, B**). *P. falciparum* cultures (Cam3.II-rev; trophozoite stage; 30-35 h p.i.) were exposed to *Pf*DHFR inhibitor, WR99210 (**A**) or protein translation inhibitor, cycloheximide (**B**) for 6 h. Protein translation was assessed in the last two hours of the incubation, via the incorporation of O-propargyl-puromycin (OPP). Aliquots of inhibitor-exposed cultures were washed and returned to cultures, and viability was estimated at the trophozoite stage of the next cycle. WR99210: IC_{50} (Translation) > 1 μM, IC_{50} (Viability) = 0.9 nM. Cycloheximide: IC_{50} (Translation) = 331 nM, IC_{50} (Viability) > 1 μM. Error bars correspond to SEM of three independent experiments. (**C**) Trophozoite stage Cam3.II_rev parasites (30-35 h p.i.) were incubated with 0.05% DMSO (Mock), 50 nM borrelidin (BOR) or 2.5 μM OSM-S-106 or 2.5 μM OSM-S-137 for 3 h. Western blots of lysates were probed for phosphorylated-eIF2α with *Pf*BiP as a loading control. The data represent additional blots related to data presented in Fig. 2B.

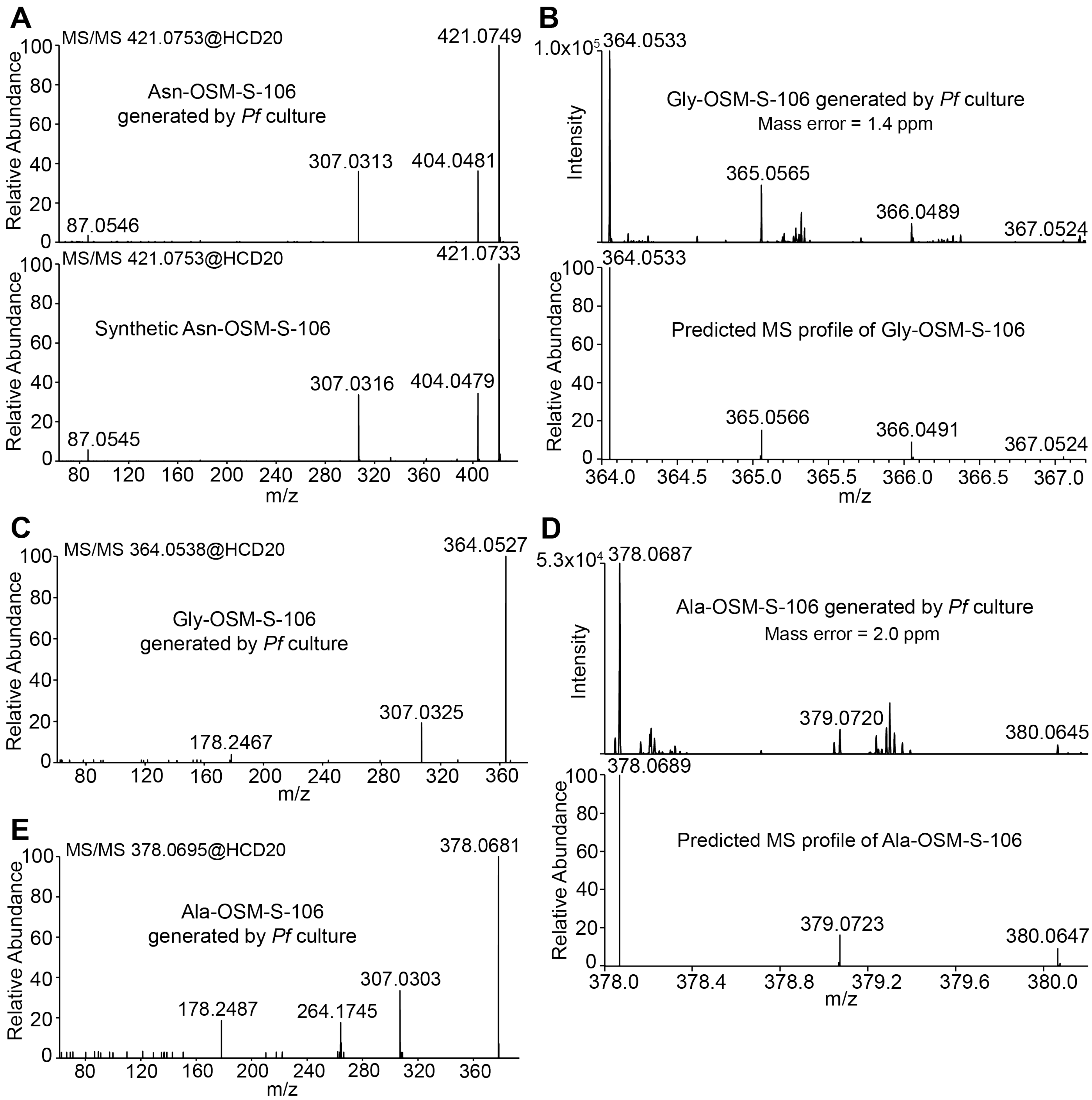

Supplementary Figure 2. **Production of Asn-OSM-S-106, Gly-OSM-S-106 and Ala-OSM-S-106 adducts by *P. falciparum* cultures.**

P. falciparum cultures were treated with 10 µM OSM-S-106 for 3 h. Extracts were subjected to LCMS. (**A**) MS/MS analysis of detected Asn-OSM-S-106 adduct compared with the synthetic conjugate. MS (**B, D**) and MS/MS (**C, E**) analysis of detected Gly-OSM-S-106 (**B, C**) and Ala-OSM-S-106 (**D, E**) from cell extracts. (**B, D**) Upper panels show the protonated adducts made by the parasites and lower panels show the predicted mass spectra.

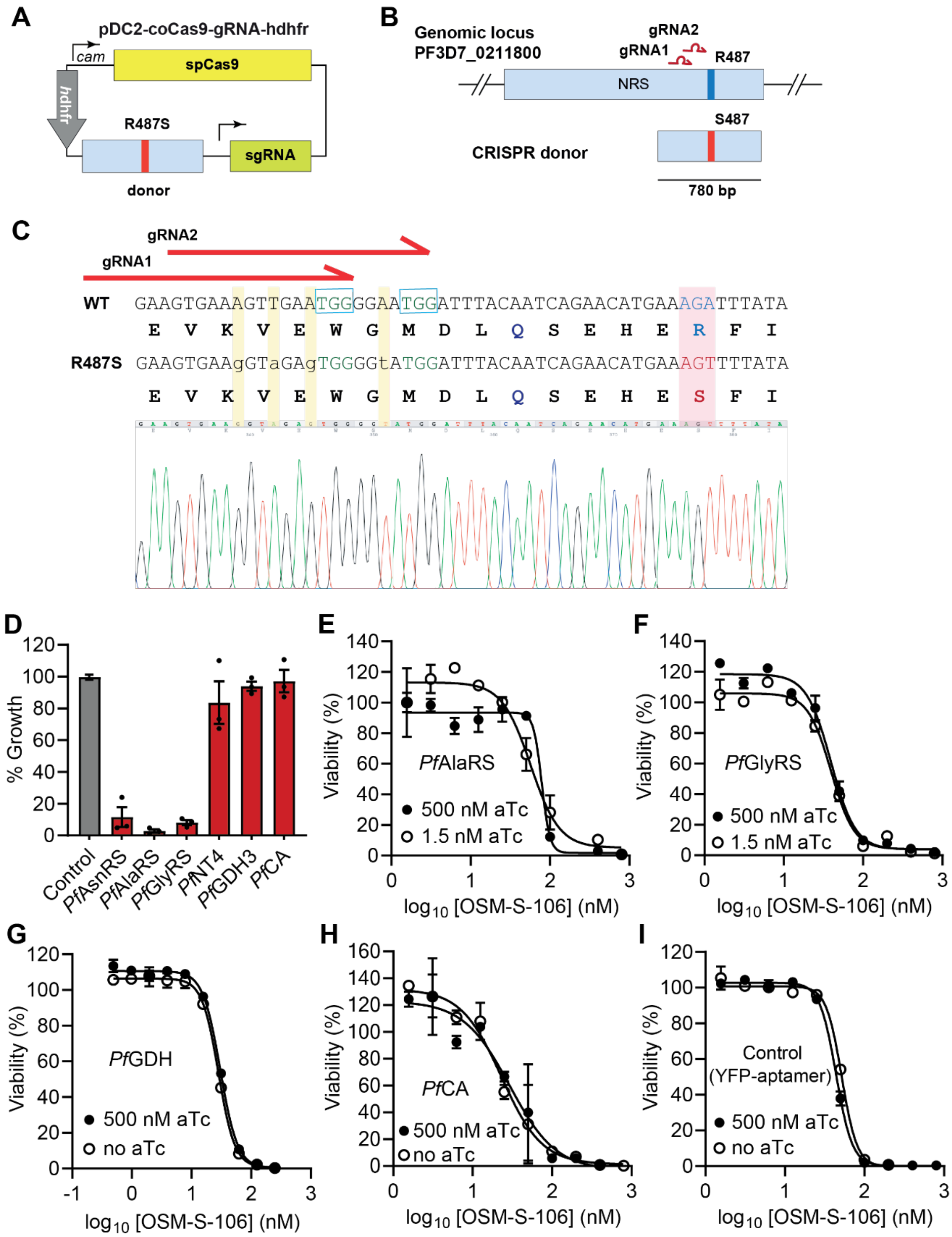

Supplementary Figure 3. Generation of CRISPR-edited *P. falciparum* transfectants harbouring the AsnRS R487S mutation, and aptamer-regulatable lines for several genes identified as potential targets.

(**A**) Plasmid map illustrating the all-in-one Cas9-gRNA-donor plasmid used for editing the R487S mutation in *PfAsnRS* (Dd2 background). (**B**) Genomic locus of the *PfAsnRS* gene (PF3D7_0211800). The R487S mutation was edited using a 780 bp donor template. (**C**) Sequence of the edited region, indicating the binding site of the two gRNAs and their corresponding PAM motifs (blue boxes), the R487S codon (highlighted in red) as well as the silent shield mutations (yellow) that were introduced to prevent gRNA binding. (**D**) Growth of aptamer-regulatable *Pf*AlaRS, *Pf*GlyRS, *Pf*GDH3, *Pf*CA lines and a control YFP line was assessed over 72 h, relative to aTc-treated controls. Error bars correspond to SEM or range of 2-4 independent experiments. (**E-I)** Sensitivity to OSM-S-106 exposure (72-h) of aptamer-regulatable *Pf*AlaRS, *Pf*GlyRS, *Pf*GDH3, *Pf*CA lines and a control YFP line, upon addition of aTc, with data normalized to a no drug control. See Supplementary Table 7 for data values.

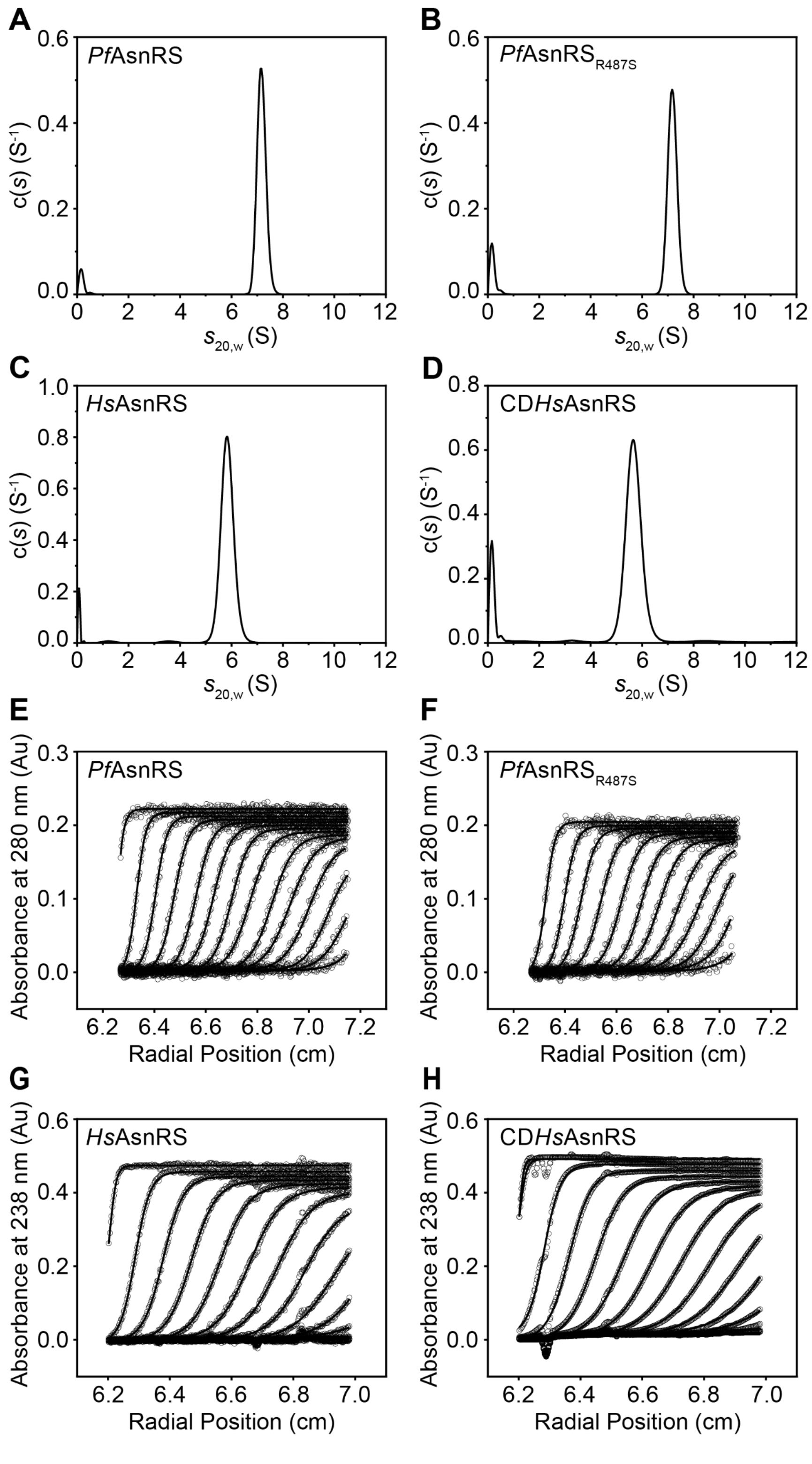

Supplementary Figure 4. Physical characterization of recombinant *Pf*AsnRS, *Pf*AsnRS$_{\mathrm{R478S}}$, full-length *Hs*AsnRS and CD*Hs*AsnRS.

Continuous sedimentation coefficient (c(s)) distributions derived from sedimentation velocity analytical ultracentrifugation for *Pf*AsnRS (**A**), *Pf*AsnRS$_{\mathrm{R478S}}$ (**B**), *Hs*AsnRS (**C**), and CD*Hs*AsnRS (**D**). The measured sedimentation coefficients (s20,w) at 2.8 μM are 7.1 S for *Pf*AsnRS, 7.1 S for *Pf*AsnRS$_{\mathrm{R478S}}$, 5.8 S for *Hs*AsnRS and 5.6 for CD*Hs*AsnRS, corresponding to masses of 141.1 kDa for *Pf*AsnRS, 141.0 kDa for *Pf*AsnRS$_{\mathrm{R478S}}$, 126.0 kDa for *Hs*AsnRS and 103.7 kDa for CD*Hs*AsnRS, all consistent with a dimeric state in solution. Raw analytical ultracentrifugation-sedimentation velocity data for (**E**) *Pf*AsnRS, (**F**) *Pf*AsnRS$_{\mathrm{R478S}}$, (**G**) *Hs*AsnRS, and (**H**) CD*Hs*AsnRS, overlaid with fits to a continuous sedimentation coefficient (c(s)) model, at 2.8 μM. For clarity, every second (**E,F**) or sixth (**G,H**) scan is shown.

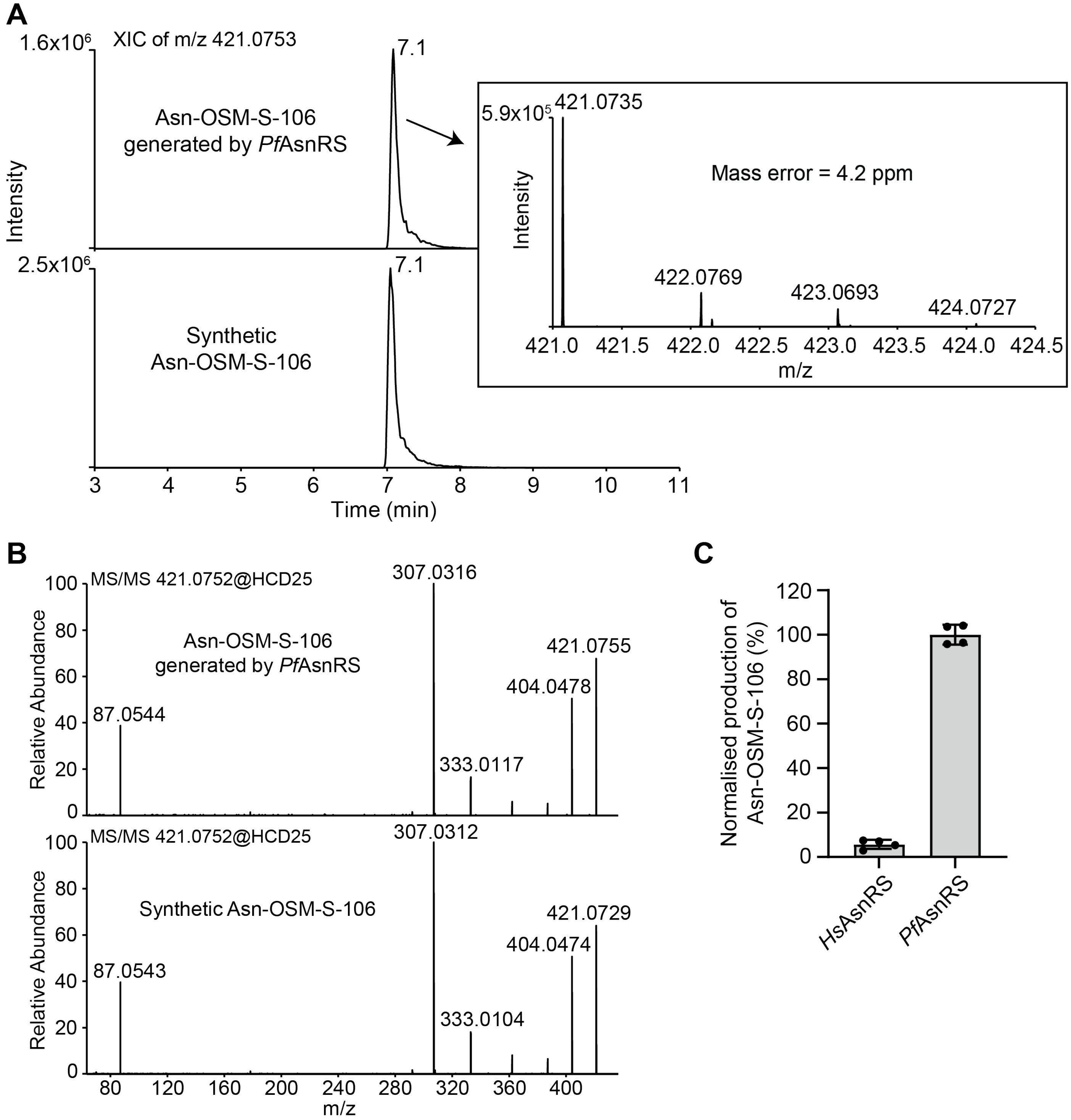

Supplementary Figure 5. **Recombinant *Pf*AsnRS is markedly more efficient in generating Asn-OSM-S-106 than *Hs*AsnRS.**

*Pf*AsnRS or *Hs*AsnRS (1 µM) was incubated with OSM-S-106 (10 µM), ATP (10 µM), asparagine (20 µM) and *E. coli* tRNA (2.5 mg/mL) for 1 h at 37°C. Following protein denaturation and precipitation, the supernatant was subjected to LCMS analysis. (**A**) The extracted ion chromatograms showing Asn-OSM-S-106 generated by *Pf*AsnRS (upper panel), and the synthetic conjugate (3 µM, lower panel), at m/z 421.0753. The inset shows MS analysis of the enzyme-generated Asn-OSM-S-106. (**B**) MS/MS analysis of *Pf*AsnRS-generated Asn-OSM-S-106 (upper panels) compared with the synthetic conjugate (lower panels). (**C**) The amount of Asn-OSM-S-106 produced by *Pf*AsnRS and *Hs*AsnRS is quantified based on the intensity of the peaks at m/z 421.0752 from the extracted ion chromatograms. Results are normalised using Asn-OSM-S-106 made by *Pf*AsnRS as 100%. Data represent mean $\pm$ SD from four independent experiments.

```
Pv  --------------------------------MGATFSRLALSTTLLGLLARGVHRGEGRAEASALALQLCGQRGRRRRAGRRAEAYGGRTTRRTATSGG  68
Pf  ----------------------------------------------------------------------------------------------------
Hs  ----------------------------------------------------------------------------------------------------
Sc  ----------------------------------------------------------------------------------------------------
Bm  ----------------------------------------------------------------------------------------------------
Eh  ----------------------------------------------------------------------------------------------------


Pv  TTRRTTPGGTPLMRGAFIFKGNAHHSWCISRGTPNALGWSTPPVRRRFFAKVRGGSGSQGGGHDGAHPGGAPNESASVAPHPGGAPHESASVAPHPGGAS 168
Pf  ------------------------------------------------------------MCEKDDITVNEEILQKAQEFQVENEKDIKMKKLKPITEGLL  41
Hs  ----------------MVLAELYVSDREGSDATGDGTKEKPFKTGLKALMTVGKEPFPTIYVDSQKENERWNVISKSQLKNIKKMWHREQMKSESREKKE  84
Sc  -------------MSSLYIKEATGVDELTTAGSQDHPFKTPAYALFASQQKSDATEPKLFVFKTEDNEYQEISASALKKARKGCDGLKKKAVKQKEQELK  87
Bm  ------------------MTVYICPETGDDGNDGSELKPLRTLYQAMIITKSSKGDFLIRTKKDGKQIWEAASKTALKKSWKHYEQEMLKNEKVAAKMLE  82
Eh  ----------------------------------------------------------------------------------------------------


Pv  NEGTSSELLPGEASNERTGRTKIKDILR--------------AQGTHSEGETCTICGWVKSVRTVGKNRFSFIDVNDGSHVKNLQVVVDAGIPNYGEVSR 254
Pf  KPE--VDLLQ-ISERGSRGRVKICNVLNVPRSEKEYNNNNSDKVENKYIGKIITVCGWSKAIRKQGGGRFCFVNLNDGSCHLNLQIVVNQCIENYEKLLK 138
Hs  -AEDSLR---REKNLEEAKKITIKNDPSLP-----EPKCVKIGALEGYRGQRVKVFGWVHRLRRQGKN-LMFLVLRDGTGYLQCVLAD--ELCQCYNGVL 172
Sc  KQQKEAE---NAAKQLSALNITIKEDESLP-----AAIKTRIYDSYSKVGQRVKVSGWIHRLRSN-KK-VIFVVLRDGSGFIQCVLSG--DLALAQQTLD 175
Bm  KDATEVG---VKAALEEAKKVQIELDTSLS-----YITGVKIRDLVKHRNERVCIKGWIHRMRRQGKS-LMFFILRDGTGFLQVVLLMD--KLCQTYDALT 171
Eh  -------------------MTEATTTPVE-----TPIVCNIRDAAGLEGKLVTFKGWAYHIRKARKT-LIFVELRDGSGYCQCVIFG-KELCEPEKVKL  73
                                                                  .:   . **   :*      . *. :.**:   :  :     :    .

Pv  VLTDDAVECSGELKCSLGKKQRVELCVRD--AARAHYVRLLGRGEGARSGGGSEGGSADGEGEKRDDGEGDRRDDGEGDRRDDGEGDRRGKHYAIAKKYH 352
Pf  CGAGCCFRFTGELIISPVQND----------NN--------------K------------KGLLKENVELALNNN-DIHNFEIYGENLDPQKYPLSKKNH 201
Hs  LSTESSVAVYGMLNLTPKGKQAPG-----GHELSCDFWELIGLAP-------AGGA--DN--------------------------------LINEES 224
Sc  LTLESTVTLYGTIVKLPEGKTAPG-----GVELNVDYYEVVGLAP---------GGEDSFTN--------------------------------KIAEGS 229
Bm  VNTECTVEIYGAIKEVPEGKEAPN-----GHELIADFWKIIGNAP---------PGGI--DN--------------------------------VLNEEA 223
Eh  LTRECSLEITGRLNAYAGKNHPPEIADILNLEMQVTEWKVIGESP---------I---DLEN--------------------------------IINKDS 129
          .    * :       :                                              .                                    :

                                                        Mobile residues
                          Motif I                      Flipping Loop                    LCR
Pv  SKEHLRSFPHLRARTKLYSCVFRLKSDVVAETFKFWRERNCTYINTPVLTSNDCEGAGELFHATTLMSRRGSGGGGEA------------------NGSD 434
Pf  GKEFLREVAHLRPRSYFISSVIRIRNSLSIATHLFFQSRGFLYIHTPLITTSDCEGGGEMFTVTTLLNENGDIRSIPRINLKNKKKEKREDILNEKNGKK 301
Hs  DVDVQLNNRHMMIRGENMSKILKARSMVTRCFRDHFFDRGYYEVTPPTLVQTQVEGGATLF--------------------------------------- 285
Sc  DPSLLLDQRHLALRGDALSAVMKVRAALLKSVRRVYDEEHLTEVTPPCMVQTQVEGGSTLF--------------------------------------- 290
Bm  SVDKMLDNRHLVIRGENAAALLRLRAAATRAMREHFYNAGYLEVAPPTLVQTQVEGGSTLF--------------------------------------- 284
Eh  SIPQKMQNRHIVIRSEHTQQVLQLRSEIQWYFRKYYHDNHFTEIQPPTIVKTQCEGGSTLF--------------------------------------- 190
    .     .  *:  *       ::: :             :  .     :  *  :.  .: **..  :*

                                                                                    Mobile residues   Motif II
             LCR
Pv  ---------------GSGGASGIDQRDERPGEPVKGDPPFCRDFFKKPCYLNVSSQLALECLCCSMGDVFTLNQSFRAEQSNTVRHLSEFLMMEVELAFS 519
Pf  DHMNDSLNNNTCNNNNNNGNSSSSNIVSSPQYEDNYLIDYKKDFFSKQAFLTVSGQLSLENLCSSMGDVYTFGPTFRAENSHTSRHLAEFWMIEPEIAFA 401
Hs  -----------------------------------------KLDYFGEEAFLTQSSQLYLETCLPALGDVFCIAQSYRAEQSRTRRHLAEYTHVEAECPFL 345
Sc  -----------------------------------------KMNYYGEEAYLTQSSQLYLETCLASLGDVYTIQESFRAEKSHTRRHLSEYTHIEAELAFL 350
Bm  -----------------------------------------NLDYFGEQSFLTQSSQLYLETCIPTLGDVFCIAQSYRAEKSRTRRHLAEYAHVEAECPFI 344
Eh  -----------------------------------------KLQYFNEPAYLTQSSQLYLESVIASLGKSFCMLSSYRAEQSRTVRHLAEYLHLEAELPFI 250
                                                     ::: :  .:*.  *.** **     ::*.  : :  ::***:*.* ***:*:   :*  *   *

Pv  NLTSIISLAEEYIKTMVKFALHQS-EDVDYIHEHHDRTLKEKLQA---VLQKPFAVVTYDEAMQIVKRHVGRVGVAVDPHVAARTDVSQRGGAPPPRADL 615
Pf  DLYDNMELAEAYIKYCIDYVLNNNFHDIYYFEENVETNLIKRLKN---ILNEDFAKITYTNAIEILQNYSDSFEVKVE------------------WGMDL 481
Hs  TFDDLLNRLEDLVCDVVDRILKSP-AGS----------IVHELNPNFQPPKRPFKRMNYSDAIVWLKEHDVKKE---------------DGTFYEFGEDI 419
Sc  TFDDLLQHIETLIVKSVQYVLEDPIAGP----------LVKQLNPNFKAPKAPFMRLQYKDAITWLNEHDIKNE---------------EGEDFKFGDDI 425
Bm  TLDDLMEKIEELVCDTVDRLLADEEAKK----------LLEHINPKFQPPERPFLRMEYKDAIKWLQEHNVENE---------------FGNTFTYGEDI 419
Eh  SFEDLLNHLEDLVCTVIDNVMAV--HGD----------KIRKMNPHLKLPTRPFKRMTYADAIKYCNDHGILNK---------------D-KPFEYGEDI 322
      : . :.  *  :   :.  :                    ..::          *  : *:*:   : :                           *:

    Mutant PfAsnRS R487S                                                                         Motif III
Pv  TFEEQRFLTEVHFRSPVVVVNYPQEIKPFYMALNAD-GKTVACMDVLLPHVGEVVGGSEREIRIHTLERRMKEKRLDLRLYEPYLQLRRQGNVPHAGFGL 714
Pf  QSEHERFIAEKIFKKPVIVYNYPKDLKAFYMKLNED-NKTVAAMDVLVPKIGEVIGGSQREDNLERLDKMIKEKKLNIDSYWWYRQLRQYGSHPHAGFGL 580
Hs  PEAPERLMTDTI-NEPILLCRFPVEIKSFYMQRCPEDSRLTESVDVLMPNVGEIVGGSMRIFDSEEILAGYKREGIDPTPYYWYTDQRKYGTCPHGGYGL 518
Sc  AEAAERKMTDTI-GVPIFLTRFPVEIKSFYMKRCSDDPRVTESVDVLMPNVGEITGGSMRIDDMDELMAGFKREGIDTDAYYWFIDQRKYGTCPHGGYGI 524
Bm  AEAAERFMTDTI-NKPILLNRFPSEIKAFYMQRDAQDNTLTESVDLLMPGVGEIVGGSMRIWKFDELSKAFKNVEIDPKPYYWYLDQRLYGTCPHGGYGL 518
Eh  SEKPERQMTDEI-GCPIFMIHFPSKMKAFYMSKVPGHPDLTESVDLLMPGVGEIVGGSMRIWNYDELMGAYKANGLNPDPYYWYTQQRKYGSCPHGGYGL 421
         :* :::      *:.:.:* .:* ***            . .:*:*:* :**: *** *     . :     *    ::    *   : : *   *.  **.*:*:

    Motif III
Pv  GVDRLIMFLTSMSNIRDVVPFPRAPGSLFM 744
Pf  GFERLIMLVTGVDNIKDTIPFPRYPGHAEF 610
Hs  GLERFLTWILNRYHIRDVCLYPRFVQRCTP 548
Sc  GTERILAWLCDRFTVRDCSLYPRFSGRCKP 554
Bm  GLERFICWLTNTNHIRDVCLYPRFVGRCVP 548
Eh  GVERLVMWLLGEDHIRKVCLYPRYLERCEP 451
    * :*::  : .   ::.   :**
```

Supplementary Figure 6. Sequence alignment of AsnRS sequences from a range of species.
Type II aaRSs exhibit three motifs (I-III, grey shading) involved in ATP binding and dimerization. Alignment of AsnRS sequences from *Homo sapiens* (Hs), *Saccharomyces cerevisiae* (Sc), *Brugia malayi* (Bm), *Entamoeba histolytica* (Eh), *Plasmodium vivax* (Pv), and *Plasmodium falciparum* (Pf) reveals a high level of conservation

of these motifs. One *Plasmodium*-specific feature of interest is the presence of a large insert (highlighted in black, white font). R487 (Pf) and the equivalent residue in other species are indicated (bold, underline type). The mobile residues that are stabilised upon ligand binding in the active site are boxed. The flipping loop that has previously been shown to undergo dynamic motions that facilitate tRNA binding (Schmitt et al., 1998) is highlighted in khaki green.

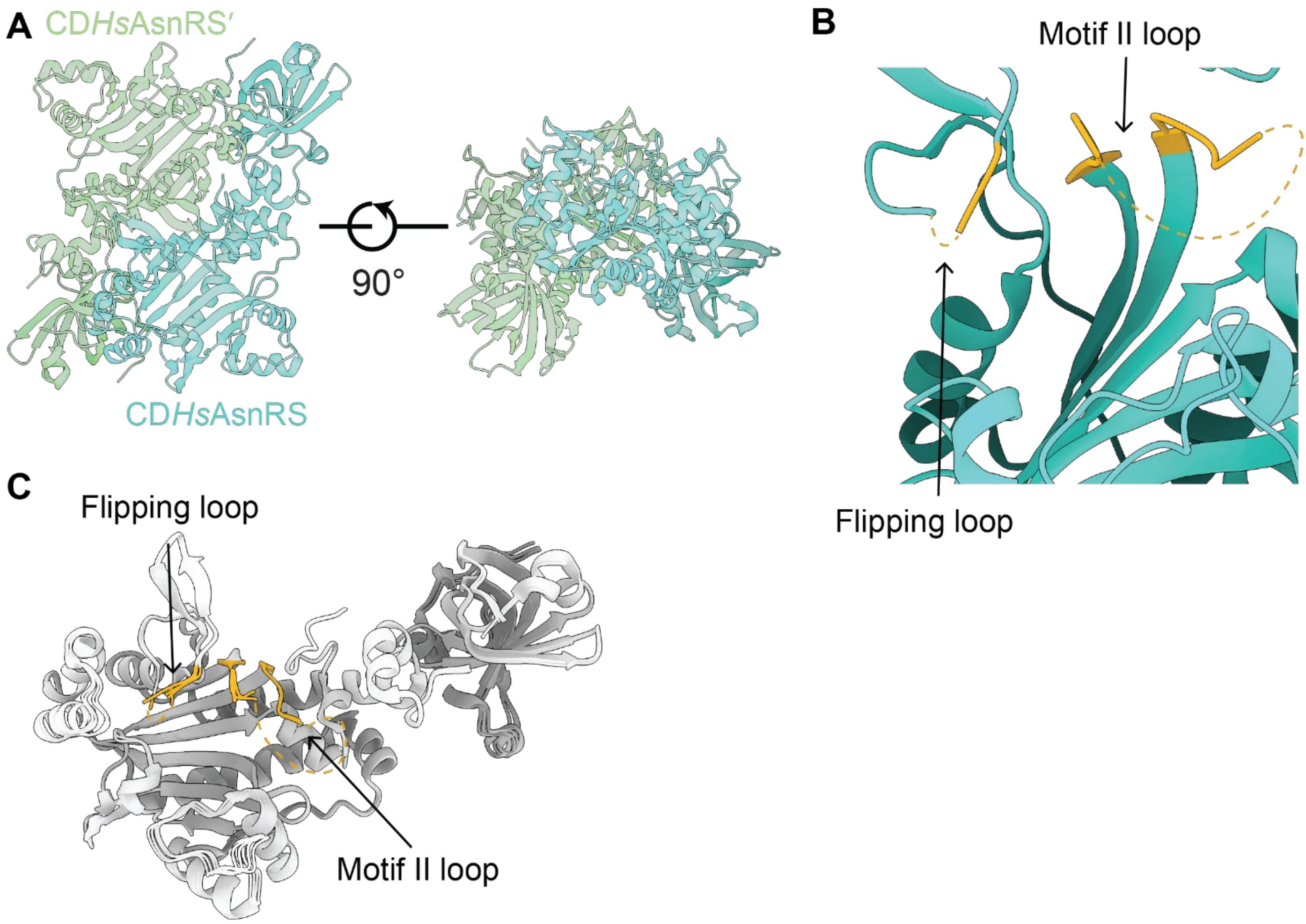

Supplementary Figure 7. Crystal structure of apo CD*Hs*AsnRS.
(**A**) Ribbon diagram of the structure of dimeric apo CD*Hs*AsnRS showing monomers in light green and teal. (**B**) Ribbon diagram of the active site of apo CD*Hs*AsnRS showing the positions of the flipping loop (E279 - T283) and motif II loop (Y321 - E334) (yellow). Density for some residues in these loops was insufficient to allow modeling, and missing residues are represented as dashed yellow lines. (**C**) Overlay of the four protein chains in the asymmetric unit, with the flipping loop and motif II loop indicated. Residues in these loops that could not be modelled are indicated as dashed yellow lines.

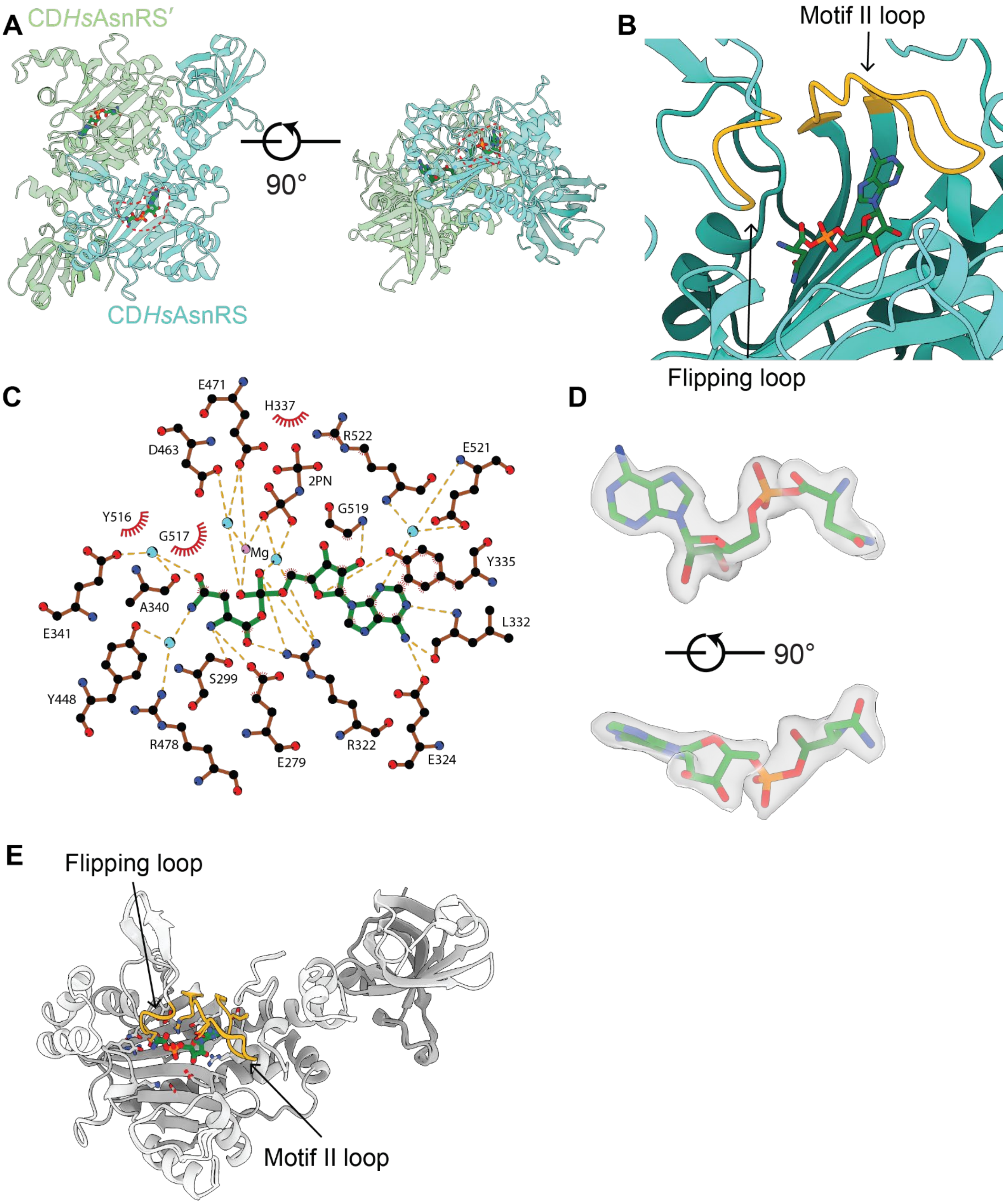

Supplementary Figure 8. Crystal structure of CD*Hs*AsnRS in complex with Asn-AMP.
(**A**) Ribbon diagram of the structure of the dimeric CD*Hs*AsnRS/Asn-AMP complex showing monomers in light green and teal. Asn-AMP is shown in stick representation (circled with dashed red line). (**B**) Ribbon diagram of the active site of the CD*Hs*AsnRS/Asn-AMP complex showing the positions of the flipping loop (E279 - T283) and motif II loop (Y321 - E334) (yellow). Asn-AMP is shown in stick representation. 2PN = imidodiphosphoric acid. (**C**) LigPlot map of interacting residues for the CD*Hs*AsnRS/Asn-AMP complex. (**D**) Composite omit maps (2mFo-DFc), showing electron density supporting the position of Asn-AMP. Maps contoured at 1.5 σ. (**E**) Overlay of the four protein chains in the asymmetric unit, with the flipping loop and motif II loop indicated.

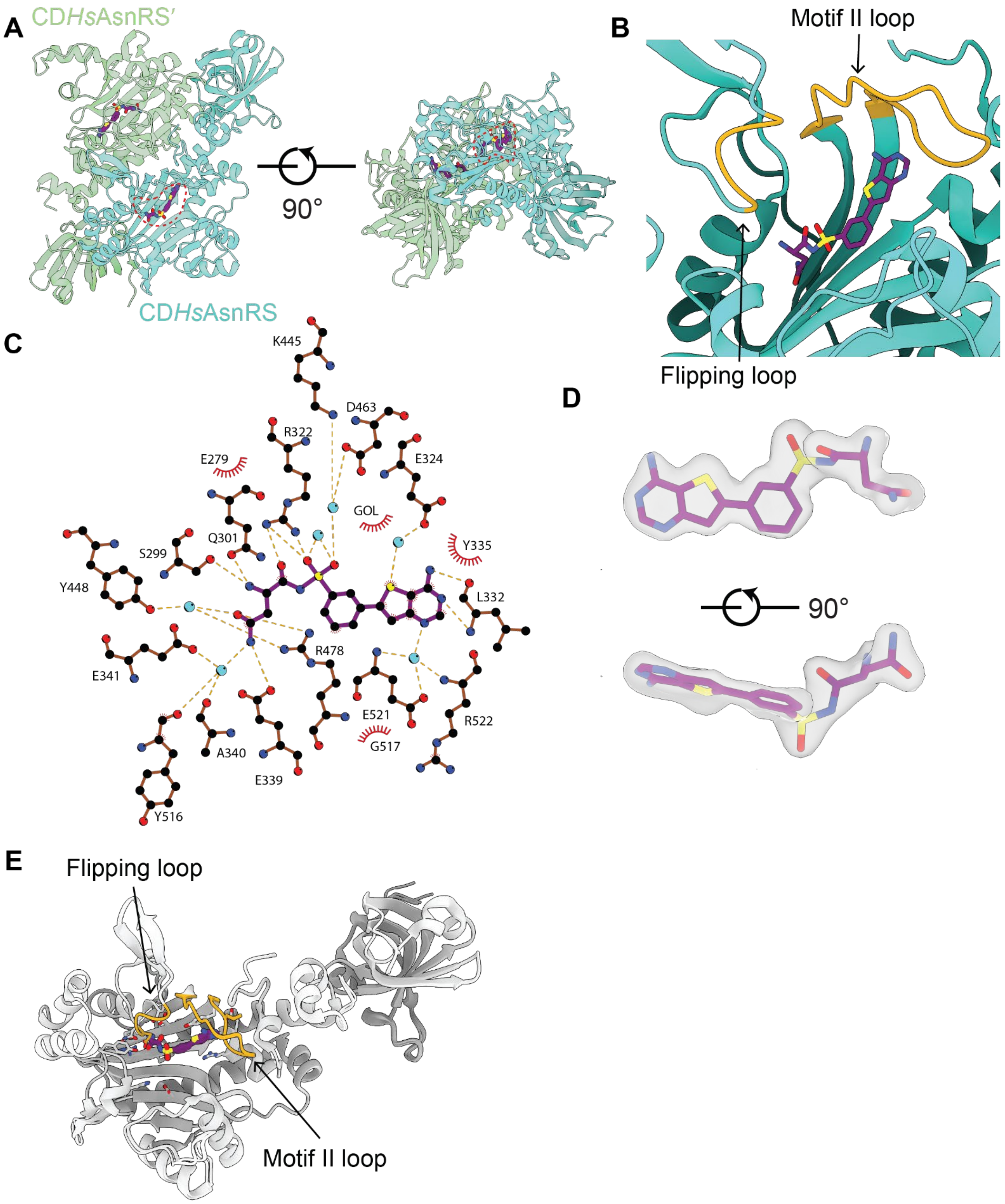

Supplementary Figure 9. Crystal structure of CD*Hs*AsnRS in complex with Asn-OSM-S-106.
(**A**) Ribbon diagram of the structure of the dimeric CD*Hs*AsnRS/Asn-OSM-S-106 complex showing monomers in light green and teal. Asn-OSM-S-106 is shown in stick representation (circled with dashed red line). (**B**) Ribbon diagram of the active site of the CD*Hs*AsnRS/Asn-OSM-S-106 complex showing the positions of the flipping loop (E279 - T283) and motif II loop (Y321 - E334) (yellow). Asn-OSM-S-106 is shown in stick representation. (**C**) LigPlot map of interacting residues for the CD*Hs*AsnRS/Asn-OSM-S-106 complex. GOL = glycerol. (**D**) Composite omit maps (2mFo-DFc) showing electron density supporting the position of Asn-OSM-S-106. Maps contoured at 1.5 σ. (**E**) Overlay of the four protein chains in the asymmetric unit, with the flipping loop and motif II loop indicated.

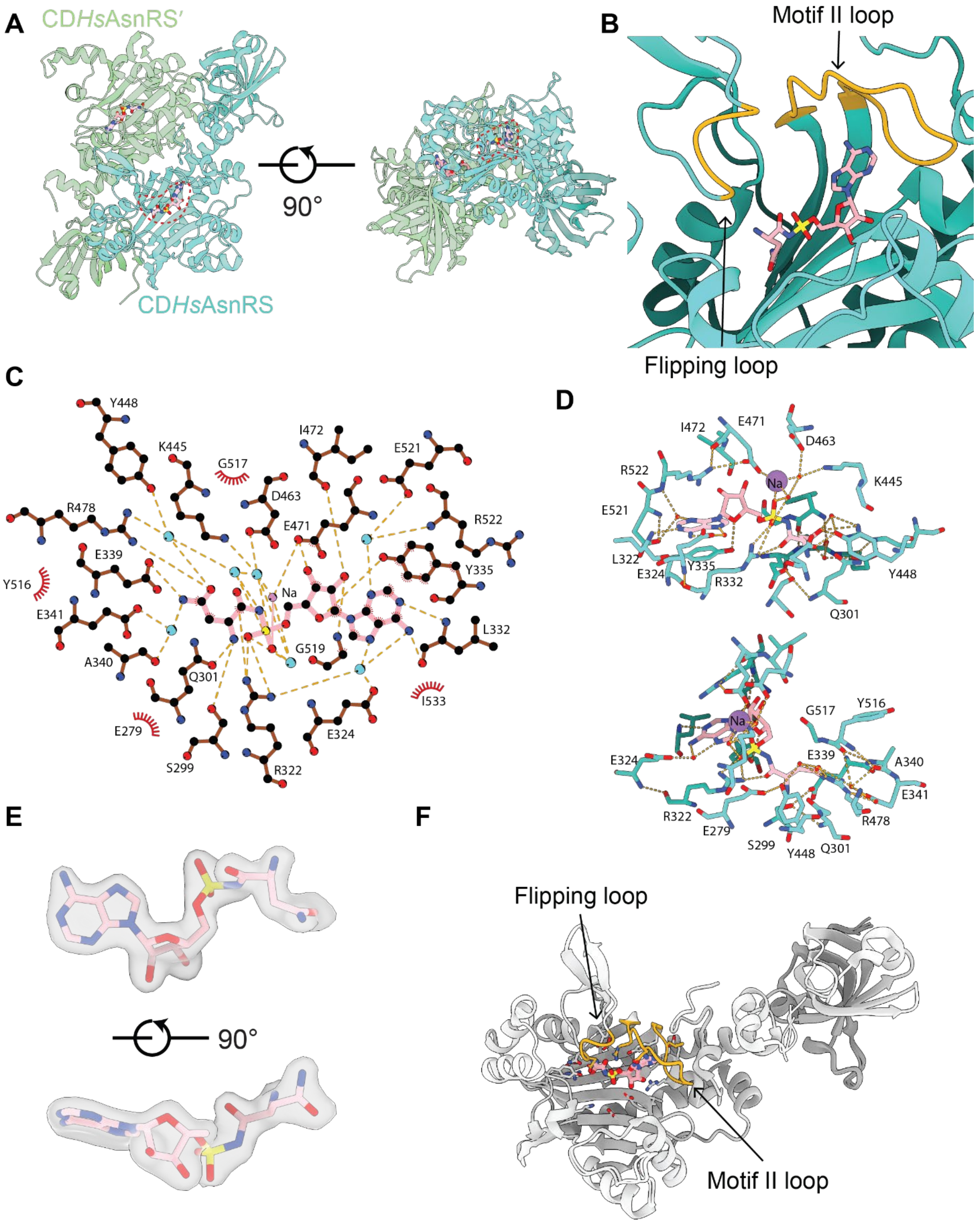

Supplementary Figure 10. Crystal structure of CD*Hs*AsnRS in complex with Asn-AMS.
(**A**) Ribbon diagram of the structure of the dimeric CD*Hs*AsnRS/Asn-AMS complex showing monomers in light green and teal. Asn-AMS is shown in stick representation (circled with dashed red line). (**B**) Ribbon diagram of the active site of the CD*Hs*AsnRS/Asn-AMS complex showing the positions of the flipping loop (E279 - T283) and motif II loop (Y321 - E334) (yellow). Asn-AMS is shown in stick representation. (**C**) LigPlot map of interacting residues for the CD*Hs*AsnRS/Asn-AMS complex. (**D**) Key inhibitor contact residues in the CD*Hs*AsnRS/Asn-AMS complex. Hydrogen bonds are indicated by yellow dashed lines. Two orientations of the complex are shown. (**E**) Composite omit maps (2mFo-DFc) showing electron density supporting the position of Asn-AMS. Maps contoured at 1.5 σ. (**F**) Overlay of the four protein chains in the asymmetric unit, with the flipping loop and motif II loop indicated.

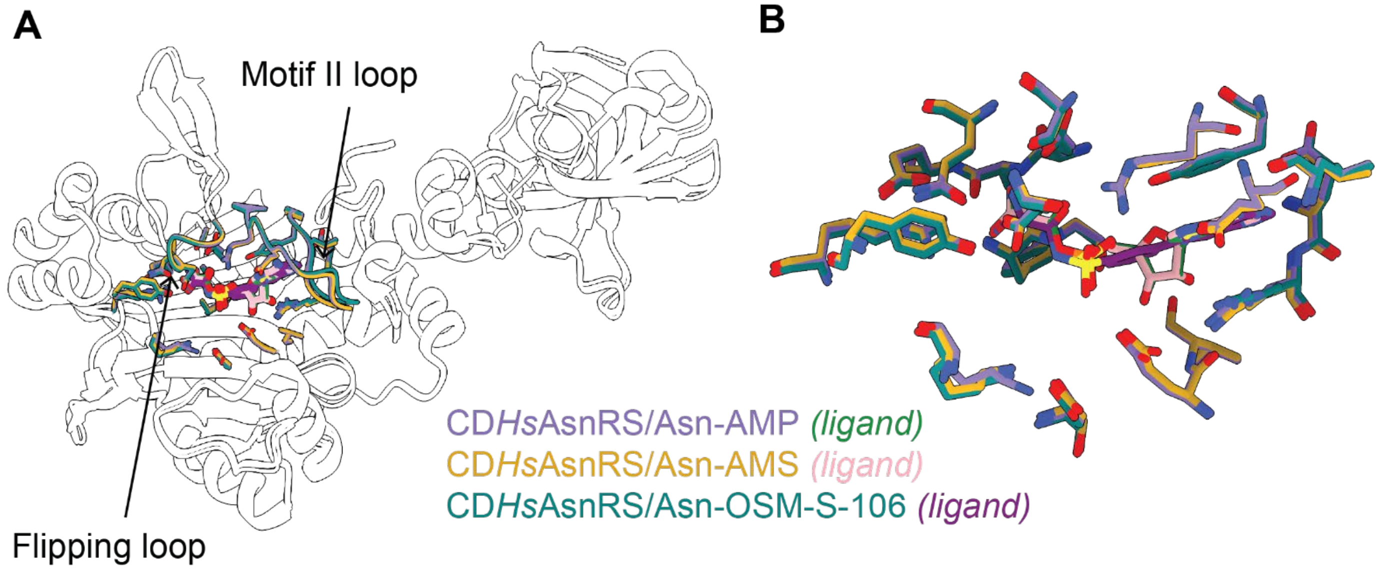

Supplementary Figure 11. Overlay of the structures of CD*Hs*AsnRS in complex with Asn-AMP, Asn-AMS and Asn-OSM-S-106. The flipping loop (E279 - T283), motif II loop (Y321 - E334) and interacting residues are shown in (**A**). A close-up view of the interacting residues is shown in (**B**). Backbone residues and ligands are colored as indicated in the figure.

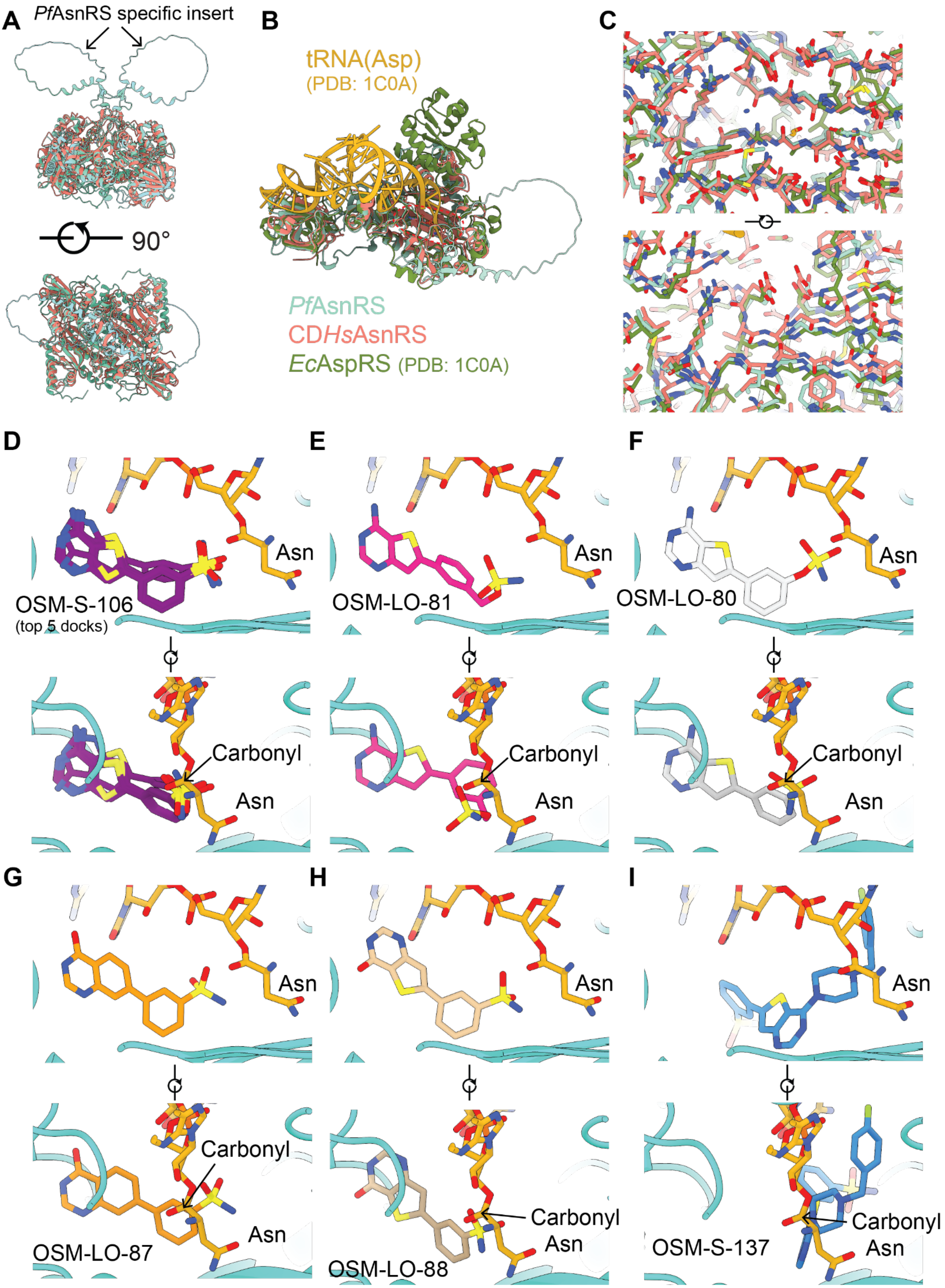
A
PfAsnRS specific insert
90°
B
tRNA(Asp)
(PDB: 1C0A)
PfAsnRS
CDHsAsnRS
EcAspRS (PDB: 1C0A)
C
D
Asn
OSM-S-106
(top 5 docks)
Carbonyl
Asn
E
Asn
OSM-LO-81
Carbonyl
Asn
F
Asn
OSM-LO-80
Carbonyl
Asn
G
Asn
Carbonyl
Asn
OSM-LO-87
H
Asn
Carbonyl
Asn
OSM-LO-88
I
Asn
OSM-S-137
Carbonyl
Asn

Supplementary Figure 12. The *Pf*AsnRS-Asn-tRNA complex model and compound docking

(**A**) Alignment of the crystal structure of the CD*Hs*AsnRS dimer (salmon) with the predicted model of the *Pf*AsnRS dimer (teal). (**B**) Alignment of the *Pf*AsnRS-Asn-tRNA model monomer (teal) with the crystal structure of the CD*Hs*AsnRS dimer (salmon) and the *E. coli* AspRS/tRNA(Asp) complex (PDB ID 1C0A, (Eiler et al., 1999)). (**C**) Zoom of the active site of the alignment in (**B**) showing close superposition of the active site structure. (**D**) Overlay of the top five *in silico* docks of OSM-S-106 to the *Pf*AsnRS-Asn-tRNA model illustrating rotation of the sulfonamide. (**E**)-(**I**) Representative *in silico* docks of compounds to the *Pf*AsnRS-Asn-tRNA model for (**E**) OSM-LO-81, (**F**) OSM-LO-80, (**G**) OSM-LO-87, (**H**) OSM-LO-88 and (**I**) OSM-S-137. Two orientations of each docked compound are shown to illustrate alignment of the reactive groups with the Asn-tRNA carbonyl carbon.

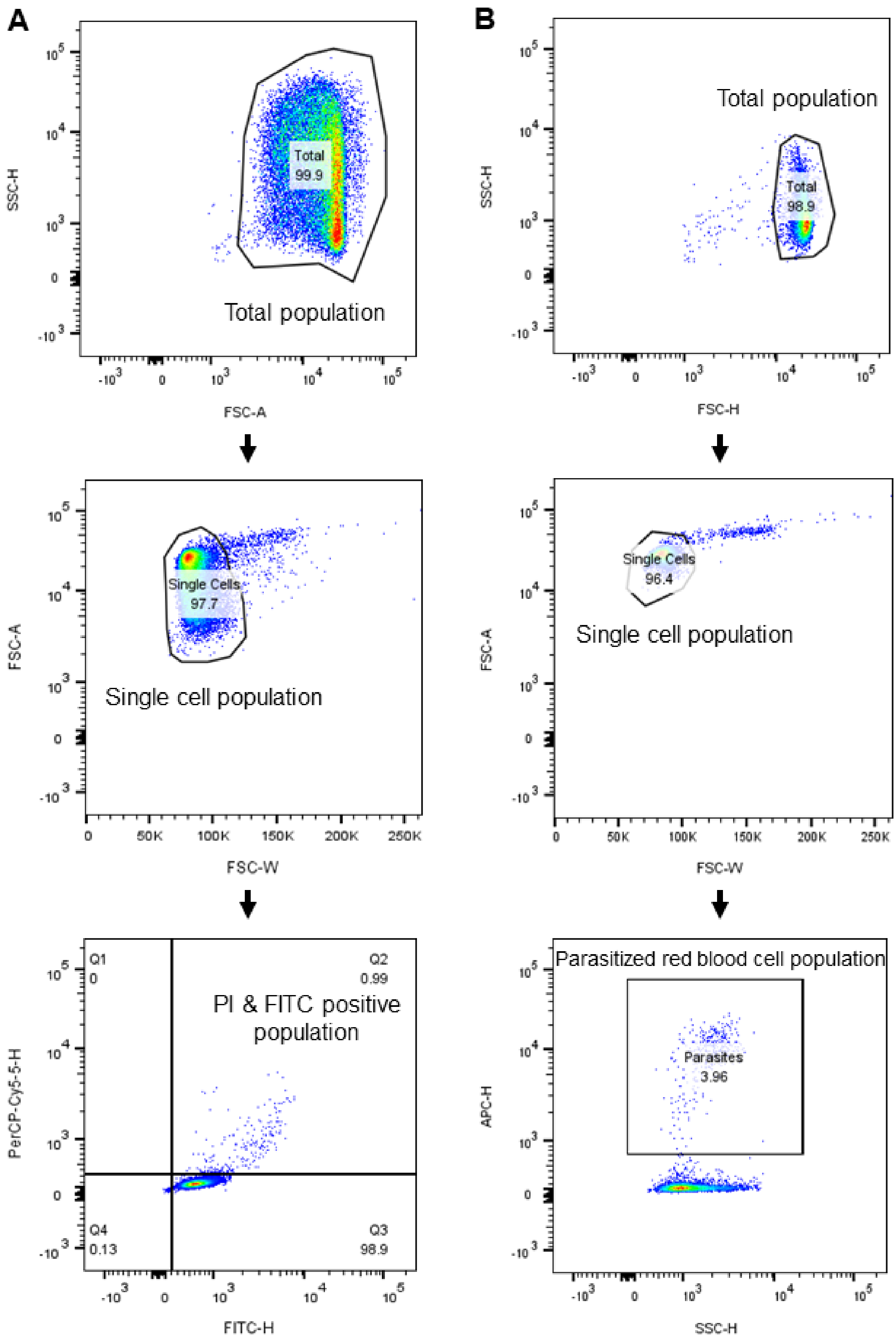

Supplementary Figure 13. Flow cytometry gating strategies used for P. falciparum cell-based translation assay (A) and viability assay (B).

Top panels: Side scatter height (SSC-H) and forward scatter area (FSC-A)/forward scatter height (FSC-H) density plots were used to gate the total cell population. Middle panels: A FSC-A and forward scatter width (FSC-W) density plot was used to select the single cell population. Bottom panel in (A): Parasitized red blood cells are propidium iodide (PI) positive and detected using PerCP-Cy5-5-H channel. FITC channel was used to detect Alexa Fluor 488 that labelled the incorporated O-propargyl-puromycin (OPP). Bottom panel in (B): Parasitized red blood cells were Syto61 positive and detected using an APC-H vs SSC-H density plot. The gating strategies were applied to calculate killing and translation inhibition activity of the compounds shown in Fig. 2A and Supplementary Fig. 1A,B.

Supplementary Tables

Supplementary Table 1. Physicochemical properties of OSM-S-106

Mass	306 Da
Solubility	pH 2: 50 – 100 μg/mL; pH 6.5: 12.5 – 25 μg/mL
Calculated logP/ ALogP	1.49/ 1.65
Number of rotatable bonds	2
Polar Surface Area/ TPSA	149/ 112 $Å^2$
Molecular Species	Neutral
Hydrogen bond acceptors/ donors	5/ 2
Ro5 Violations	0

Supplementary Table 2. ADME properties of OSM-S-106

System	Degradation half-life (min)	Intrinsic clearance (CL) (μL/min/mg protein)
Human microsomes	395 / 619	3.5 / 2.2
Mouse microsomes	19.7 / 20.4	70.5 / 68.1

Supplementary Table 3. Changes in bulk recrudescent culture sensitivity to DSM265 following evolution of resistance.

Two recrudescent wells (G7 and F3) showed 3.1 × IC_{50}, and 2.5 × IC_{50} shifts, respectively.

	**Dd2-B2 (parent)**	**DSM265_G7**	**DSM265_F3**
**Mean IC_{50} (nM) $\pm$ Standard Error of the Mean (SEM) (N)**	22.6 $\pm$ 0.9 (3)	71.0 $\pm$ 1.8 (3)	56.6 $\pm$ 3.8 (3)
**Fold Shift**	N/A	3.1	2.5

Supplementary Table 4. Sequencing statistics for DSM265-selected parasites subjected to whole-genome sequencing.

Whole-genome sequencing revealed a CNV amplification of ~2× within a 438 kb segment on chromosome 6 in G7, and a CNV amplification of ~1.4× within a 36 kb segment, also on chromosome 6, in one clone (Supp Dataset 2). Both segments contain *Pf*DHODH, the amplification of which is known to mediate low-level resistance to DSM265.

		**DSM265 treated clones**		**Parent**
**Sample names**		**F3**	**G7**	**Dd2_B2_Parent**
**Total reads**		4,542,517	2,914,074	4,680,824
**# Mapped reads**		3,803,690	2,703,481	4,342,594
**Duplication rate**		28.05%	20.01%	31.51%
**General error rate**		1.99%	2.08%	1.71%
**Mean mapping quality (Phred)**		56.47	56.33	56.74
**Depth of coverage**	**mean**	28.60	21.45	40.85
	**SD**	33.49	37.34	37.53

**% of PF genome with > x no. reads**	**1X**	96.15%	96.01%	96.07%
	**5X**	93.94%	92.65%	94.20%
	**10X**	90.83%	85.51%	91.81%
	**20X**	75.98%	55.19%	
	**30X**	46.59%	17.49%	71.42%

Supplementary Table 5. Sequencing statistics for ramp-up OSM-S-106-selected parasites subjected to whole genome sequencing.

**Sample Name**	**Mean Whole Genome Coverage (x)**	**Number of Reads Aligned to 3D7 Reference**	**Proportion of Total Reads Aligned to 3D7 Reference**
Dd2-B2-Parent-11-2018	98.94	27645387	0.937
Dd2-OSM-S-106-3-E5	107.19	30413089	0.984
Dd2-OSM-S-106-3-H7	95.07	26571029	0.984
Dd2-B2-Parent-12-2020	136.19	47589603	0.978
Dd2-OSM-S-106-2A6	146.06	56424362	0.981
Dd2-OSM-S-106-2A9	153.35	52393477	0.978
Dd2-OSM-S-106-2B2	168.67	58309916	0.977
Dd2-OSM-S-106-2D6	122.4	45467320	0.983

Supplementary Table 6. Activity of OSM-S-106 and control compounds against CRISPR-edited *Pf*AsnRS^{R487S} mutant lines. The data points represent the mean ± S.D. from five independent experiments.

**CRISPR-edited *Pf*AsnRS^{R487S} mutant lines**		
**Parasite line**	**Dd2 parent**	***Pf*AsnRS^{R487S} mutant lines**
IC_{50} (μM)	0.071 $\pm$ 0.008	0.165 $\pm$ 0.016

Supplementary Table 7. Activity of OSM-S-106 against knockdown lines. n = Number of biological repeats, each of which has three technical repeats. The data points represent the mean ± SEM (where available).

**Knockdown line**	**Mean IC_{50} (μM) with 500 nM aTc**	**Mean IC_{50} (μM) with low (1 or 1.5 nM)/ no aTc**	**Average fold-shift**
*Pf*AsnRS	0.14 $\pm$ 0.02 (n = 3)	0.026 $\pm$ 0.006 (n = 3)	6.6
*Pf*AlaRS	0.078/ 0.100 (n = 2)	0.057/ 0.046 (n = 2)	1.8
*Pf*GlyRS	0.039/ 0.037 (n = 2)	0.040/ 0.041 (n = 2)	1.0
*Pf*NT4	0.021 $\pm$ 0.001 (n = 3)	0.007 $\pm$ 0.001 (n = 3)	3.0
*Pf*GDH3	0.040 $\pm$ 0.010 (n = 4)	0.046 $\pm$ 0.015 (n = 4)	1.0
*Pf*CA	0.033 $\pm$ 0.003 (n = 3)	0.026 $\pm$ 0.002 (n = 3)	1.2

Supplementary Table 8. Crystallographic data collection and refinement statistics.

	CD*Hs*AsnRS (Apo)	CD*Hs*AsnRS/ Asn-AMP	CD*Hs*AsnRS/ Asn-AMS	CD*Hs*AsnRS/ Asn-OSM-S-106
**Data collection**				
Space group	$P2_12_12_1$	$P2_12_12_1$	$P2_12_12_1$	$P2_12_12_1$
Wavelength (Å)	0.95373	0.9796	0.95373	0.95373
Number of images	1800	180	1800	1800
Oscillation range per image (˚)	0.1	1.0	0.1	0.1
X-ray source	Australian Synchrotron beamline MX2	PAL BL-5C	Australian Synchrotron beamline MX2	Australian Synchrotron beamline MX2
Detector	Eiger 16M	Q315r	Eiger 16M	Eiger 16M
Cell dimensions				
a, *b*, *c* (Å)	113.49, 127.19, 163.16	115.42, 125.84, 161.13	115.85, 126.29, 160.60	115.09, 126.81, 161.14
α, β, γ (˚)	90, 90, 90	90, 90, 90	90, 90, 90	90, 90, 90
Resolution range for data processing (Å)	49.05-1.90 (1.93-1.90)	50.00-2.16 (2.20-2.16)	49.29-1.90 (1.93-1.90)	49.46-2.00 (2.03-2.00)
$R_{sym}^{\dagger}$	0.108 (2.021)	0.160 (1.069)	0.176 (2.916)	0.195 (2.522)
$R_{meas}^{\S}$	0.117 (2.426)	0.172 (1.149)	0.191 (3.152)	0.212 (2.718)
$R_{pim}^{\ddagger}$	0.044 (0.921)	0.063 (0.420)	0.072 (1.186)	0.080 (1.035)
$CC_{1/2}$	0.99 (0.513)	0.993 (0.868)	0.998 (0.502)	0.997 (0.471)
I/σ(I)	9.5 (0.8)	12.3 (2.1)	7.5 (0.7)	6.4 (0.8)
Total observations	1279483 (60343)	931105	1277523 (62399)	1093766 (52694)
Unique reflections	185343 (8986)	126122 (6260)	185028 (9037)	159099 (7783)
Completeness (%)	99.8 (98.7)	100.0 (100.0)	99.9 (99.3)	99.9 (99.7)
Multiplicity	6.9 (6.7)	7.4 (7.4)	6.9 (6.9)	6.9 (6.8)
Wilson *B* factor ($Å^2$)	32.2	30.8	27.5	32.6
**Refinement**				
Resolution (Å)	46.58-1.90 (1.97-1.90)	49.88-2.16 (2.24-2.16)	49.29-1.90 (1.97-1.90)	49.46-2.0 (2.07-2.00)
Reflections used in refinement	185022 (18240)	125294 (12332)	184540 (18148)	158766 (15664)
R_{free} reflections	9064 (873)	6229 (646)	9326 (914)	7948 (778)
R_{work}	0.186 (0.380)	0.203 (0.278)	0.181 (0.343)	0.182 (0.315)
R_{free}	0.221 (0.407)	0.237 (0.321)	0.217 (0.373)	0.221 (0.335)
Protein molecules in asymmetric unit	4	4	4	4
Total nonhydrogen atoms	15123	14571	15820	15457
Protein	13884	13501	14007	14080
Ligand/ion	134	324	224	346
Solvent	1105	746	1589	1031
Mean B *factor (Å²)*	44.9	39.9	37.0	41.3
Protein	44.7	39.9	36.4	41.1
Ligand/ion	55.0	42.6	35.5	48.7
RMS deviations				
Bond lengths (Å) (outliers >4σ)	0.013 (1)	0.003 (0)	0.007 (0)	0.007 (0)
Bond angles (°) (outliers >4σ)	1.172 (2)	0.58 (0)	0.861 (0)	0.873 (0)
Rotamer outliers	0.72	0.63	0.46	0.65

Clashscore	2.81	3.51	3.70	3.45
Cβ outliers	0	0	0	0
Molprobity score	1.09	1.34	1.41	1.35
Ramachandran Plot				
Favoured (%)	97.92	96.82	96.28	96.63
Allowed (%)	1.78	3.18	3.72	3.31
Outliers (%)	0.30	0	0	0.06
PDB code	8TC7	8H52	8TC8	8TC9

† $R_{\mathrm{sym}} = \Sigma_{hkl}\Sigma_i \, |I_i(hkl) \quad \langle I(hkl)\rangle \quad| / \Sigma_{hkl}\Sigma_i I_i(hkl)$

§ $R_{\mathrm{meas}} = \Sigma_{hkl} \, [N/(N \; 1)]^{½} \, \Sigma_i \, |I_i(hkl) \quad \langle I(hkl)\rangle \quad| / \Sigma_{hkl} \, \Sigma_i \, I_i(hkl)$

‡ $R_{\mathrm{pim}} = \Sigma_{hkl} \, [1/(N \; 1)]^{½} \, \Sigma_i \, |I_i(hkl) \quad \langle I(hkl)\rangle \quad| / \Sigma_{hkl} \, \Sigma_i \, I_i(hkl)$

$CC_{1/2}$ = Pearson correlation coefficient between independently merged half datasets

Supplementary Table 9. List of oligonucleotides for gene knockdown donor vector construction.

Description	Nucleotide sequence
AsnRS cKD RHR forward	gtacggtacaaacccggaattcgagctcggATGTGTATGCCCTTATAATTTACAT
AsnRS cKD RHR reverse	aagacgagagattgggtattagacctagggataacagggtaatGACAACATGCAAGACAAAGAACTCTTTG
AsnRS sgRNA target site	CCACATGCAGGATTTGGTTT
AlaRS cKD RHR forward	gtacggtacaaacccggaattcgagctcggGCCATATACATTGTTTAGCTAATTAAC
AlaRS cKD RHR reverse	aagacgagagattgggtattagacctagggataacagggtaatCACATGAACAACGATCGAATA
AlaRS sgRNA target site	GTCACTAATGTGATAGGTGT
GlyRS cKD RHR forward	gtacggtacaaacccggaattcgagctcggCCATGTATGTATATTTTTACGTATG
GlyRS cKD RHR reverse	aagacgagagattgggtattagacctagggataacagggtaatTTCATCACAAGAACAAGTCTGATCA
GlyRS sgRNA target site	AAAGGGTATACCGATTTCAT
NT4 cKD RHR forward	gtacggtacaaacccggaattcgagctcggGGTTTTGTAATGCAATAATTCATTTGTAATTTGAAC
NT4 cKD RHR reverse	aagacgagagattgggtattagacctagggataacagggtaatGAAAAATATACCCTGTACCTTTTATATTCCTTC
NT4 sgRNA target site	GAAATATAAGATCACTGAGT
GDH3 cKD RHR forward	gtacggtacaaacccggaattcgagctcggCATTATATATTGCAGTACTCCCATATGTTG
GDH3 cKD RHR reverse	aagacgagagattgggtattagacctagggataacagggtaatCTGGATACACATCATCAAGTTACTC
GDH3 sgRNA target site	ATGATGATGACTATTATCCT
CA cKD RHR forward	gtacggtacaaacccggaattcgagctcggGATAAATACGATTAATTAGGTTTG
CA cKD RHR reverse	aagacgagagattgggtattagacctagggataacagggtaatTGGGTCTACAAAAAAAACTG
CA sgRNA target site	GTATCAAGTGCAGTTCACGT

(RHR, right homology region; cKD, conditional knockdown

Chemical Methods

Chemical abbreviations

Asn: Asparagine
AMS: Acyl-sulfonyladenosine
Boc = *tert*-Butoxycarbonyl
DCM: Dichloromethane

DIPEA = *N,N*-Diisopropylethylamine
DMA = *N,N*-Dimethylacetamide
DMAP: 4-Dimethylaminopyridine
DMF = *N,N*-Dimethylformamide
EDCl: 1-Ethyl-3-(3-dimethylaminopropyl)carbodiimide
Et: Ethyl group
HATU = 1-[bis(dimethylamino)methylene]-1H-1,2,3-triazolo[4,5-*b*]pyridinium 3-oxide hexafluorophosphate
LDA: Lithium diisopropylamide
TBAF = Tetrabutylammonium fluoride
TBSCl = *tert*-Butyldimethylsilyl chloride
TFA = Trifluoroacetic acid
THF = Tetrahydrofuran

General information 1

Reagents were purchased from Sigma-Aldrich, Merck, Fisher Scientific, Apollo Scientific, and Fluorochem and were used without further purification unless specified. Anhydrous conditions: glassware was dried at >130 °C for >12 h, assembled hot, and allowed to cool under a high vacuum or purged with inert gas where suitable. Nitrogen (N_2) and argon (Ar) gas were used as obtained, from a cylinder. On a Schlenk line, the phrase *in vacuo* equates to 10 mbar. Reduced pressure means 900 to 50 mbar under rotary evaporation at 40 °C. Davisil Grace Davison 40 – 63 µm (230 – 400 mesh) silica gel and a Biotage Isolera One or Biotage Selekt were used for automated flash chromatography. Analytical thin-layer chromatography (TLC) was carried out on Merck Silica Gel 60 F_{254}-precoated aluminum plates (0.2 mm) and observed using UV irradiation (254 nm and 280 nm) and staining with potassium permanganate, anisaldehyde, or ninhydrin. High-temperature reactions were carried out in either temperature-controlled silicone oil baths or DrySyn heating blocks.

Melting points (M.P.) were measured using a Stanford Research Systems OptiMelt instrument at 1 °C min^{-1} (capillaries = 1.5 – 1.6 mm, 90 mm). Without atmospheric adjustment, infrared spectroscopy was performed using a Bruker Alpha-E (attenuated total reflectance) and analysed with Microlab PC software. The samples were examined neat. Bruker spectrometers were used for nuclear magnetic resonance spectroscopy at 300 K: AVANCE III 400 (^{1}H at 400 MHz, ^{13}C at 101 MHz), or AVANCE III 500 (^{1}H at 500 MHz, ^{13}C at 126 MHz). Spectra were analysed with Mestrelab Research Mnova. Deuterated solvents ($CDCl_3$ and DMSO-d_6) were obtained from Sigma-Aldrich. ^{1}H and ^{13}C chemical shifts are reported in parts per million (ppm). Spectroscopic chemical shifts were calibrated to residual solvent peaks (^{1}H: $CHCl_3$ 7.26 ppm, dimethyl sulfoxide (DMSO) 2.50 ppm; ^{13}C: $CHCl_3$ 77.16 ppm, DMSO 39.52 ppm). The multiplicities are described as either a singlet (s), doublet (d), triplet (t), quartet (q), quintet (qn) or multiplet (m).

High resolution mass spectrometry was carried out on Agilent 6545XT AdvanceBio LC/Q-TOF with ESI ionisation. The charge of the ion specifies whether the detection is positive or negative; for instance, $[M+H]^+$ denotes positive-ion detection. Analytical liquid chromatography-mass spectrometry (LCMS) was performed on an Agilent Infinity 1290 II system consisting of a quaternary pump (G7111A) and a diode array detector WR (G7115A) coupled to a InfinityLab LC/MSD (G6125B) using ESI. An Agilent Poroshell 120 EC-C18 column (2.7 µm, 4.6 x 50 mm) was eluted at a flow rate of 1.5 mL/min with a mobile phase of 0.05 % formic acid in H_2O and 0.05 % formic acid in MeCN. Preparative LCMS was performed on a combined Agilent Infinity 1260 II and Infinity 1290 II system consisting of a preparative binary pump (G7161A) and a multiple wavelength detector (G7165A) coupled to a InfinityLab LC/MSD (G6125B) using ESI and a preparative open-bed fraction collector (G7159B). An Agilent Eclipse XDB-C18 column (5 µm, 9.4 x 250 mm) was eluted at a flow rate of 5 mL/min with a mobile phase of 0.05% formic acid in H_2O and 0.05% formic acid in MeCN. As determined by NMR spectroscopy, the purity of all substances exceeded 95%.

General information 2 (for Asn-AMS synthesis)

Reagents: Reagents were obtained from Aldrich Chemical (www.sigma-aldrich.com) or Acros Organics (www.fishersci.com) and used without further purification. Optima or HPLC grade solvents were obtained from Fisher Scientific (www.fishersci.com), degassed with Ar, and purified on a solvent drying system unless otherwise indicated.

Reactions: All reactions were performed in flame-dried glassware under positive Ar pressure with magnetic stirring unless otherwise noted. Liquid reagents and solutions were transferred through rubber septa via syringes flushed with Ar prior to use.Cold baths were generated as follows: 0 °C, wet ice/water; –10 °C, wet ice/brine; –20 °C, dry ice/isopropanol monitored with a thermometer; –44 °C, dry ice/CH_3CN; –63 °C, dry ice/chloroform; –78 °C, dry ice/acetone; –100 °C, dry ice/Et_2O.

Chromatography: TLC was performed on 0.25 mm E. Merck silica gel 60 F254 plates and visualized under UV light (254 nm) or by staining with potassium permanganate ($KMnO_4$), cerium ammonium molybdate (CAM), phosphomolybdic acid (PMA), iodine (I_2), or *p*-anisaldehyde. Silica flash chromatography was performed on E. Merck 230–400 mesh silica gel 60. Analytical to semi-preparative HPLC was carried out on a Waters Alliance 2695 HPLC with 2996 diode array detector with a Higgins Analytical Targa C18 reverse phase column (5 cm × 4.6 mm, 3 um, 120 Å), using a flow rate of 1.0 mL/min and a gradient of 5 – 95% CH_3CN in 0.1% aq TFA over 5 min with UV detection at 254 nm and ELSD detection.

Analytical instrument: NMR spectra were recorded on a Bruker UltraShield Plus 500 MHz Avance III NMR or UltraShield Plus 600 MHz Avance III NMR with DCH CryoProbe at 24 °C in $CDCl_3$ unless otherwise indicated. Chemical shifts are expressed in ppm relative to TMS (^1H, 0 ppm) or solvent signals: $CDCl_3$ (^{13}C, 77.0 ppm), C_6D_6 (^1H, 7.16 ppm; ^{13}C, 128.0 ppm) or acetone-d_6 (^{13}C, 206.2 ppm); coupling constants are expressed in Hz. NMR spectra were processed using Bruker TopSpin, Mnova (www.mestrelab.com/software/mnova-nmr), or nucleomatica iNMR (www.inmr.net) software. Mass spectra were obtained at the MSK Analytical Core Facility on a Waters Acuity SQD LC-MS or PE SCIEX API 100 by electrospray (ESI) ionization. High resolution mass spectra were obtained on a Waters Acuity Premiere XE TOF LC-MS by electrospray ionization (ESI).

Nomenclature: N.B.: Atom numbers in chemical structures herein refer to the standard nucleoside numbering system used in the text of the article and Supporting Information and not to IUPAC nomenclature, which was used solely to name each compound. Compounds not cited in the paper are numbered herein from S1.

General Synthetic Procedure

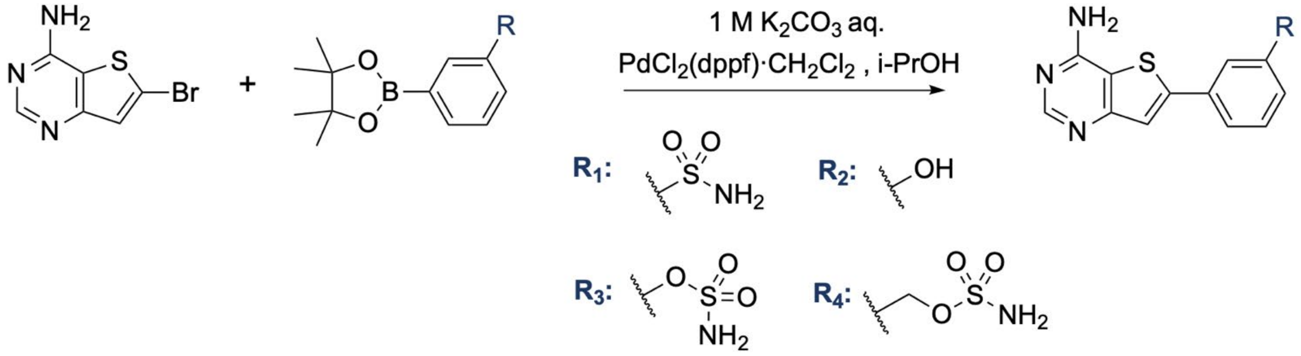

General procedure 1: Suzuki reaction between thienopyrimidine core and functionalised aryl halides.

Organohalide (1 equiv.), pinacol boronate (1.1 equiv.) and $PdCl_2$(dppf)·DCM (0.1 equiv.) were combined. *i*-PrOH and 1 M aq. K_2CO_3 (1 – 3 equiv. for the stated condition) were added and the reaction mixture was heated using conventional heating or microwave irradiation for the stated time after being degassed with Ar. The reaction mixture was diluted with MeOH, filtered through celite, and concentrated under reduced pressure to give a residue that was purified by automated flash chromatography on silica to give the coupled product.

Synthesis and Characterisation of Compounds

Synthesis of OSM-S-106

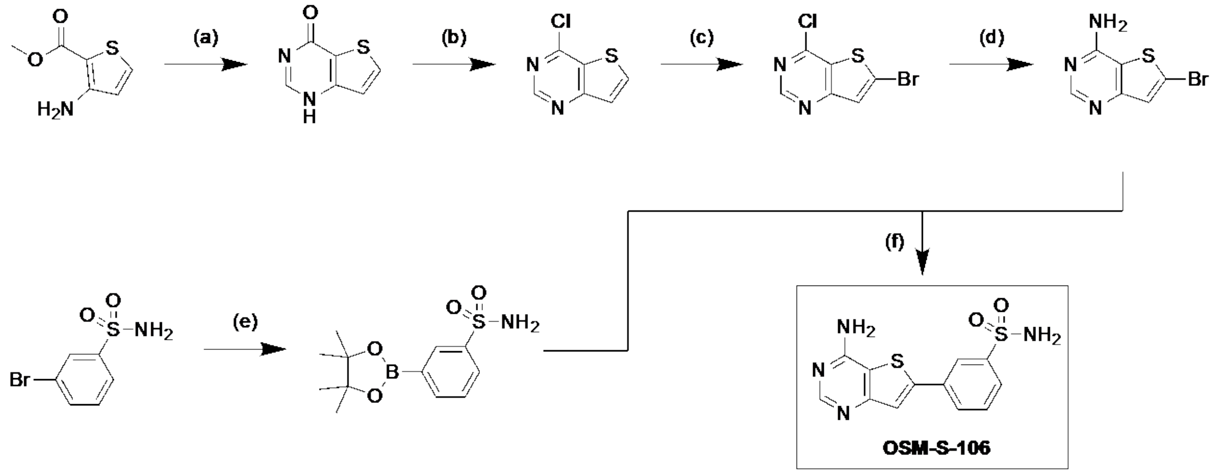

Supplementary Figure 14: Synthetic route to OSM-S-106. Reagents and conditions: (a) ammonium formate, formic acid, formamide, 140 °C, 23 h; (b) $POCl_3$, reflux, 2.5 h; (c) THF, –78 °C, 10 min *then n*-BuLi, –78 °C, 40 min *then* Br_2, –78 °C, rt, 1 h ; (d) 28% NH_4OH solution, isopropanol, 120 °C, 18 h; (e) bis(pinacolatodiboron), potassium acetate, dioxane, $PdCl_2$(dppf)·DCM, microwave, 140 °C, 60 min; (f) General procedure 1: $PdCl_2$(dppf)·DCM, 1 M aq. K_2CO_3, *i*-PrOH, microwave, 90 °C, 90 min

Thieno[3,2-*d*]pyrimidin-4(1*H*)-one (1)

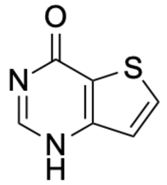

1

Methyl-3-aminothiophene-2-carboxylate (2.79 g, 17.8 mmol, 1 equiv.), ammonium formate (1.19 g, 18.8 mmol, 1 equiv.) and formic acid (0.73 mL, 19 mmol, 1.1 equiv.) were combined to form a slurry. Formamide (4.30 mL, 100 mmol, 5 equiv.) was added, and the reaction mixture was stirred at 140 °C for 23 h. The solution was allowed to cool to rt, and the solvent was removed under reduced pressure. The resulting solid was washed with H_2O and then filtered and dried *in vacuo* to give *the title compound* as a cream needle-like solid (1.78 g, 67%); R_F = 0.28 (100% EtOAc); M.P. 219 – 220 °C (lit. (Song, 2007): 222 – 223 °C); ^{1}H NMR (500 MHz, $CDCl_3$) δ 8.16 (s, 1 H), 7.88 (d, *J* = 5.4 Hz, 1 H), 7.42 (d, *J* = 5.4 Hz, 1 H), 7.26 (s, 1 H) ppm; ^{13}C NMR (101 MHz, $CDCl_3$) δ 157.8, 159.2, 146.5, 135.9, 135.2, 112.8 ppm. The NMR data matched those in the literature (Wang et al., 2017) (Woodring et al., 2015).

4-Chlorothieno [3,2-*d*]pyrimidine (2)

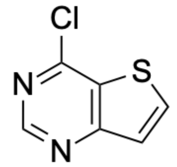

2

Phosphoryl chloride (13.7 mL, 147 mmol, 9.9 equiv.) was added to compound **1** (2.28 g, 15.0 mmol, 1 equiv.) under Ar. The reaction was heated at 110 °C until completion as indicated by TLC. The reaction mixture was allowed to cool to rt and neutralised to pH 7 with 4 M NaOH solution in an ice bath whilst stirring. The aqueous mixture was extracted with EtOAc (3 ×) and the combined organic layers were washed with brine, dried ($MgSO_4$), filtered and concentrated under reduced pressure to give the crude product which was purified by automated flash chromatography on silica (Biotage Isolera, 12 – 100% EtOAc in hexane) to give *the title compound* as a cream solid (0.87 g, 40%). R_F = 0.41 (20% EtOAc in hexane); M.P. 122.1 – 123.0 °C (lit. (Song, 2007) 123 – 124 °C); ^{1}H NMR (500 MHz, $CDCl_3$) δ 8.94 (s, 1 H), 8.00 (d, *J* = 5.5 Hz, 1 H), 7.55 (d, *J* = 5.5 Hz, 1 H); ^{13}C NMR (126 MHz, $CDCl_3$) δ 161.9, 155.0, 154.3, 137.0, 130.7, 125.1. The NMR data matched those in the literature (Ham et al., 2010) (Woodring et al., 2015).

6-Bromo-4-chlorothieno[3,2-*d*]pyrimidine (3)

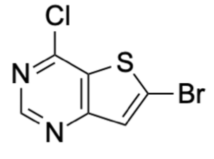

3

Compound **2** (4.55 g, 26.7 mmol, 1 equiv.) was dissolved in dry THF (100 mL) and stirred at –78 °C under an Ar atmosphere for 20 min. *n*-Butyllithium (1.09 M in hexane (Burchat et al., 1997), 26.0 mL, 28.4 mmol, 1.1 equiv.) was added to the solution at –78 °C. The stirring was continued at –78 °C for 40 min before bromine (1.50 mL, 29.3 mmol, 1.1 equiv.) was added. The reaction was stirred at rt for a further 1 h and quenched with sat. aq. $Na_2S_2O_3$ solution (100 mL). The solvent was removed under reduced pressure. Sat. aq. $Na_2S_2O_3$ solution (20 mL) and EtOAc (20 mL) were added to the residue, and the organic layer was separated. The aqueous phase was extracted with EtOAc (3 ×) and the combined organic layers were washed with H_2O (2 ×), brine (2 ×), dried ($MgSO_4$), filtered and concentrated under reduced pressure to give the crude product which was purified by automated flash chromatography on silica (Biotage Isolera, 0 – 15% EtOAc in hexane) to give *the title compound* as a light-yellow solid (4.07 g, 61%). R_F = 0.48 (10% EtOAc in hexane); M.P. 135.0 –135.5 °C (no lit. M.P.); ^{1}H NMR (500 MHz, $CDCl_3$) δ 8.94 (s, 1 H), 7.62 (s, 1 H) ppm; ^{13}C NMR (126 MHz, $CDCl_3$) δ 160.5, 153.8, 152.2, 131.5, 127.9, 126.8. The NMR data matched those in the literature (Woodring et al., 2015).

6-Bromothieno[3,2-*d*]pyrimidin-4-amine (4)

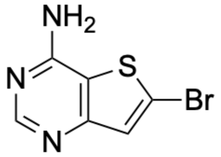

4

Compound **3** (35.2 mg, 140 mmol, 1.00 equiv.) was dissolved in isopropanol (4 mL). Ammonium hydroxide solution (28%, 900 mM, 0.550 mL, 140 mmol, 100 equiv.) was added and the reaction was heated in an oil bath set at 120 °C for 18 h. A 1:1 solution of EtOAc and H_2O (20 mL) was added, and the aqueous phase was extracted with EtOAc (3 ×). The combined organic layers were washed with brine (2 ×), dried ($MgSO_4$), filtered and concentrated under reduced pressure to give the crude product which was purified by automated flash chromatography on silica (Biotage Isolera, 15 – 100% EtOAc in hexane) to give *the title compound* as a white solid (28.0 mg, 87%). R_F = 0.11 (50% EtOAc in hexane); M.P. 241 – 242 °C (no lit. M.P.); ^{1}H NMR (400 MHz, $CDCl_3$) δ 8.33 (s, 1 H), 7.55 (s, 1 H), 7.50 (s, 2 H, NH_2) ppm; ^{13}C NMR (101 MHz, $CDCl_3$) δ 159.6, 157.0, 155.4, 127.6, 122.4, 115.4; *m/z* (ESI+) 231 ([M+H]$^+$, 100%); HRMS (ESI+) found 231.9369 ([M+H]$^+$), $C_6H_4BrN_3S^+$ requires 231.9309.

3-(4,4,5,5-Tetramethyl-1,3,2-dioxaborolan-2-yl)benzenesulfonamide (5)

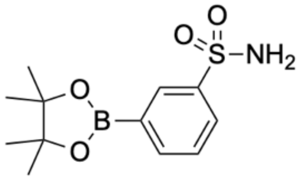

5

3-Bromobenzenesulfonamide (500 mg, 2.12 mmol, 1 equiv.), *bis*(pinacolatodiboron) (645 mg, 2.54 mmol, 1.2 equiv.), potassium acetate (626 mg, 6.37 mmol, 3 equiv.) and $PdCl_2$(dppf)·DCM (34.6 mg, 42.4 mmol, 0.02 equiv.) were combined in a microwave vial that was filled with Ar and evacuated three times. Dioxane (20 mL, 0.10 M) was added, and the reaction mixture was degassed before heating under microwave irradiation at 140 °C for 1 h. The reaction mixture was diluted with MeOH (10 mL) and filtered through celite. The filtrate was concentrated and the residue was purified by automated flash chromatography on silica (Biotage Isolera, 10 – 100% EtOAc in hexane) to give *the title compound* as a white solid (570 mg, 95%). R_F = 0.12 (20% EtOAc in hexane); M.P. 215 – 216 °C (no lit. M.P.); ^{1}H NMR (400 MHz, DMSO-d_6) δ 8.14 (s, 1 H), 7.91 – 7.94 (m, 1 H), 7.84 – 7.86 (m, 1 H), 7.57 – 7.61 (m, 1 H), 7.36 (s, 2 H, NH_2), 1.32 (s, 12 H); *m/z* (ESI+) 589 ($[2M+Na]^+$, 100%). The spectroscopic data matched those in the literature (Abdelsamie et al., 2017).

3-(4-Aminothieno[3,2-*d*]pyrimidin-6-yl)benzenesulfonamide (OSM-S-106)

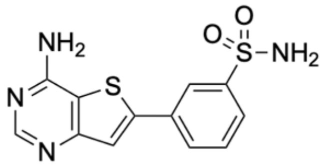

OSM-S-106

Prepared according to General Procedure 1 from: compound **4** (195 mg, 0.847 mmol, 1 equiv.), compound **5** (265 mg, 0.936 mmol, 1.1 equiv.) and $PdCl_2$(dppf)·DCM (69 mg, 0.085 mmol, 0.10 equiv.), *i*-PrOH (17 mL, 0.5 M) and 1 M aq. K_2CO_3 (2.50 mL, 0.25 mmol, 3 equiv.) under microwave irradiation at 90 °C for 1.5 h to give the crude title compound as a brown solid; purified by automated flash chromatography on silica (Biotage Isolera, 5 – 20% MeOH (with 10% MeOH): DCM) to give *the title compound* as a light brown solid (181 mg, 69%). R_F = 0.12 (10% MeOH (with 10% NH_4OH) in DCM); M.P. decomposes at 278 °C; ^{1}H NMR (400 MHz, DMSO-d_6) δ 8.39 (s, 1 H), 8.21 (s, 1 H), 8.11 (apparent dt, *J* = 8.1, 1.3 Hz, 1 H), 7.89 – 7.87 (m, 2 H), 7.72 (apparent t, *J* = 7.8 Hz, 1 H), 7.52 (s, 2 H, NH_2), 7.50 (s, 2 H, NH_2) ppm; ^{13}C NMR (101 MHz, DMSO-d_6) δ 160.4, 158.0, 155.4, 147.0, 145.2, 133.5, 130.3, 129.4, 126.3, 123.0, 121.7, 113.8; *m/z* (ESI+) 307 ($[M+H]^+$, 100%); HRMS (ESI+) found 307.0322 ($[M+H]^+$), $C_{12}H_{11}N_4O_2S_2^+$ requires 307.0318.

Synthesis of OSM-E-32

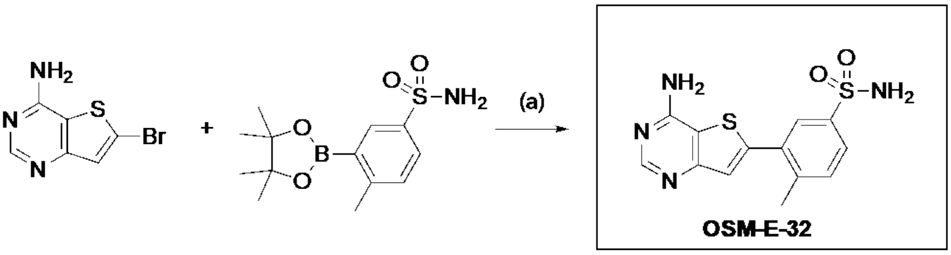

Supplementary Figure 15: Synthetic route to OSM-E-32. Reagents and conditions: (a) General procedure 1: $PdCl_2$(dppf)·DCM, 1 M aq. K_2CO_3, *i*-PrOH, microwave, 90 °C, 30 min.

3-(4-Aminothieno[3,2-*d*]pyrimidin-6-yl)-4-methylbenzenesulfonamide (OSM-E-32)

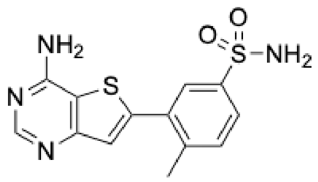

OSM-E-32

Prepared according to General Procedure 1 from: Compound **4** (28 mg, 0.10 mmol, 1 equiv.), 4-methyl-3 (4,4,5,5-tetramethyl-1,3,2-dioxaborolan-2-yl)benzenesulfonamide (47 mg, 0.12 mmol, 1.1 equiv.), and $PdCl_2$(dppf)·DCM (16 mg, 20 µmol, 20 mol%, 0.1 equiv.) were dissolved in *i*-PrOH (2 mL) and 1 M aq. K_2CO_3 (0.2 mL, 0.2 mmol, 2 equiv.) under microwave irradiation at 90 °C for 30 min to give the crude title compound; purified using column chromatography on silica (0 – 10% MeOH in DCM) to give *the title compound* as a tan solid (16.8

mg, 52%). M.P. decomposes at 293 °C; ^{1}H NMR (600 MHz, methanol-d_4) δ 8.41 – 8.42 (m, 1 H), 8.00 (s, 1 H), 7.87 – 7.89 (m, 1 H), 7.55 (d, *J* = 6.0 Hz, 1 H), 7.37 (s, 1H), 2.52 (s, 3 H) ppm (both amine and sulfonamide NH_2 signals not observed); ^{13}C NMR (151 MHz, methanol-d_4) δ 160.8, 160.3, 156.3, 150.4, 143.7, 142.8, 135.5, 133.2, 139.2, 126.3, 125.5, 116.9, 21.4; *m/z* (ESI+) 321 ([M+H]$^+$, 100%); HRMS (ESI+) found 321.0474 ([M+H]$^+$), $C_{13}H_{13}N_4O_2S_2^+$ requires 321.0480.

Synthesis of OSM-S-488

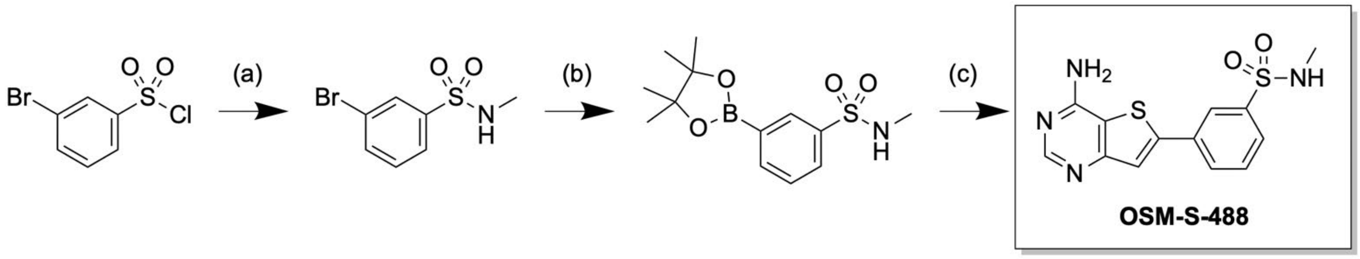

Supplementary Figure 16: Synthetic route to OSM-S-488. Reagents and conditions: (a) methylamine, Et_3N, THF, 0 °C then rt, 90 min; (b) bis(pinacolatodiboron), potassium acetate, dioxane, $PdCl_2$(dppf)·DCM, 100 °C, 18 h; (c) General procedure 1: $PdCl_2$(dppf)·DCM, 1 M aq. K_2CO_3, *i*-PrOH, microwave, 90 °C, 30 min.

3-Bromo-*N*-methylbenzenesulfonamide (9)

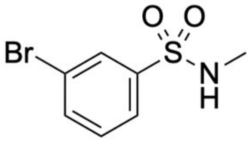

9

Methylamine (1.3 mL, 15 mmol, 5.0 equiv) and Et_3N (2.0 mL, 15 mmol, 5.0 equiv) were dissolved in 6 mL THF and the mixture was cooled to 0 °C. 3-Bromobenzenesulfonylchloride (750 mg, 2.90 mmol, 1.0 equiv) was added and the reaction was allowed to warm to rt for 90 min. The aqueous phase was extracted with EtOAc (3 ×). The combined organic layers were washed with brine (2 ×), dried ($MgSO_4$), filtered and concentrated under reduced pressure to give *the title compound* as a white solid (607 mg, 91%). ^{1}H NMR (500 MHz, $CDCl_3$) δ 8.04 (apparent t, *J* = 1.8, 1 H), 7.86 – 7.80 (m, 1 H), 7.75 (ddd, *J* = 8.0, 1.8, 1.0, 1 H), 7.44 (apparent t, *J* = 7.9, 1 H), 2.73 (d, *J* = 5.5, 3 H) ppm; ^{13}C NMR (126 MHz, $CDCl_3$) δ 140.87, 135.80, 130.66, 130.16, 125.76, 123.18, 29.39; *m/z* (ESI+) 363.3 ([M+H]$^+$, 100%). The spectroscopic data matched those in the literature (Liang et al., 2017).

N-Methyl-3-(4,4,5,5-tetramethyl-1,3,2-dioxaborolan-2-yl)benzenesulfonamide (10)

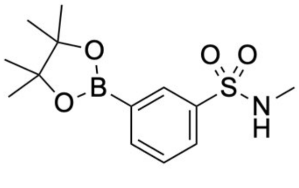

10

Compound **9** (93 mg, 0.37 mmol, 1 equiv.), *bis*(pinacolatodiboron) (284 mg, 1.12 mmol, 4.5 equiv.), potassium acetate (294 mg, 3.00 mmol, 12 equiv.) and $PdCl_2$(dppf)·DCM (30 mg, 0.070 mmol, 0.2 equiv.) were dissolved in 1,4-dioxane (4 mL) and heated at 100 °C in a sealed tube for 18 h. The mixture was concentrated under vacuum and the residue was purified by flash column chromatography (0–10% methanol in chloroform) to give *the title compound as* a brown crude solid (167.3 mg); which carried forward without further purification. R_F = 0.15 (50% EtOAc in hexane); *m/z* (ESI+) 321.1 ([M+Na]$^+$, 85%)

3-(4-Aminothieno[3,2-*d*]pyrimidin-6-yl)-*N*-methylbenzenesulfonamide (OSM-S-488)

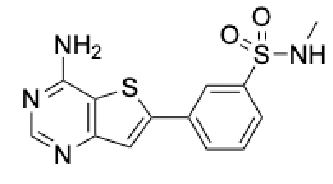

OSM-S-488

Prepared according to General Procedure 1 from: compound **4** (46 mg, 0.20 mmol, 1 equiv.), compound **10** (120 mg, 0.40 mmol, 2 equiv.), $PdCl_2$(dppf)·DCM (32 mg, 39 µmol, 20 mol%, 0.1 equiv.), *i*-PrOH (4 mL) and 1 M aq. K_2CO_3 (0.4 mL, 0.4 mmol) under microwave irradiation at 90 °C for 30 min to give the crude title compound; purified using column chromatography on silica (0 – 10% MeOH in DCM) to give *the title compound* as a pale brown solid (38.9 mg, 80%); $\mathbf{R_F}$ = 0.51 (10% MeOH in DCM); **M.P.** decomposes at 287.6 °C; ^{1}**H NMR** (400 MHz, DMSO-d_6) δ 8.40 (s, 1H), 8.18 – 8.13 (m, 2H), 7.92 (s, 1H), 7.85 (apparent dt, *J* = 7.9, 1.3 Hz, 1H), 7.77 (apparent t, *J* = 7.8 Hz, 1H), 7.62 (br d, *J* = 4.7 Hz, 1H), 7.53 (s, 2H), 2.47 (d, *J* = 3.6 Hz, 5H) ppm; ^{13}**C NMR** (101 MHz, DMSO-d_6) δ 160.4, 158.0, 155.4, 146.8, 140.4, 133.8, 130.5, 130.0, 127.3, 123.8, 121.9, 113.8, 28.7; ***m/z*** (ESI+) 321.1 ([M+H]$^+$, 100%); HRMS (ESI+) found 321.0478 ([M+H]$^+$), $C_{13}H_{20}NO_4S^+$ requires 321.0474.

Synthesis of OSM-LO-80

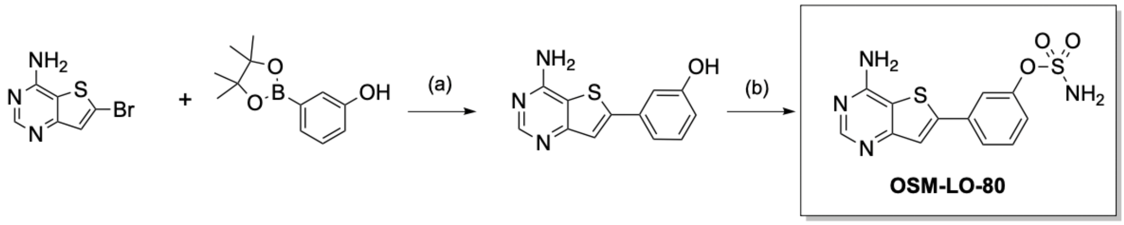

Supplementary Figure 17: Synthetic route to OSM-LO-80. Reagents and conditions: (a) $PdCl_2$(dppf)·DCM, potassium acetate, dioxane, microwave, 90 °C, 90 min; (b) sulfamoyl chloride, PhMe, DMA, 0 °C, overnight.

3-(4-Aminothieno[3,2-*d*]pyrimidin-6-yl)phenol (11)

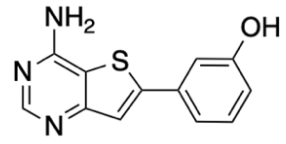

11

Prepared according to General Procedure 1 from: compound **4** (200 mg, 0.900 mmol, 1 equiv.), 3-(4,4,5,5-tetramethyl-1,3,2-dioxaborolan-2-yl)phenol (233 mg, 1.06 mmol, 1.22 equiv.), $PdCl_2$(dppf)·DCM (71 mg, 0.090 mmol, 0.1 equiv.), *i*-PrOH (17 mL, 0.5 M) and 1 M K_2CO_3 (2.61 mL, 2.61 mmol, 3 equiv.) under microwave irradiation at 90 °C for 90 min to give the crude title compound as a dark brown solid; purified by automated flash chromatography on silica (Biotage Isolera, 5 – 20% MeOH in DCM) to give *the title compound* as a brown solid (88 mg, 42%). R_F = 0.2 (10% MeOH in DCM); M.P. decomposes at 175.1 °C (no lit. M.P.); ^{1}H NMR (400 MHz, DMSO-d_6) δ 9.78 (s, 1 H), 8.36 (s, 1 H), 7.68 (s, 1 H), 7.42 (s, 2 H, NH_2), 7.33 – 7.36 (m, 2 H), 7.18 (s, 1 H), 6.87 – 6.84 (m, 1 H) ppm; ^{13}C NMR (101 MHz, DMSO-d_6) δ 160.5, 158.0, 157.9, 155.2, 149.0, 134.0, 130.4, 120.2, 117.0, 116.6, 113.1, 112.8; *m/z* (ESI+) 243.9 ([M+H]$^{+,}$ 100%). HRMS (ESI+) found 244.0566 ([M+H]$^+$), $C_{12}H_9N_3OS^+$ requires 244.0466.

3-(4-Aminothieno[3,2-*d*]pyrimidin-6-yl)phenyl sulfamate (OSM-LO-80)

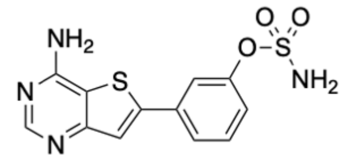

OSM-LO-80

A solution of sulfamoyl chloride (470 mg, 4.1 mmol, 10 equiv.) in PhMe (4 mL) was added dropwise to a solution of compound **6** (99 mg, 0.41 mmol, 1 equiv.) in DMA (4 mL) at 0 °C. The reaction mixture was stirred at rt overnight then quenched with H_2O (6 mL). The mixture was filtered and concentrated under reduced pressure to give a yellow crude oil. A few drops of DCM were added to form a white precipitate. The mixture was filtered and the solid was purified by reverse-phase automated flash chromatography on silica (Biotage Isolera, 5 – 50 % MeOH in H_2O) to give *the title compound* as a white solid (22 mg, 17 %). R_F = 0.55 (15% MeOH in

DCM); M.P. decomposes at 269.7 °C (no lit. M.P.); ^{1}H NMR (400 MHz, DMSO-d$_6$) δ 8.39 (s, 1 H), 8.12 (s, 2 H), 7.87 – 7.78 (m, 2 H), 7.71 (apparent t, J = 2.0 Hz, 1 H), 7.61 (apparent t, J = 8.0 Hz, 1 H), 7.51 (s, 2 H), 7.36 (ddd, J = 8.1, 2.3, 0.9 Hz, 1 H); ^{13}C NMR (101 MHz, DMSO-d$_6$) δ 160.8, 158.4, 155.6, 151.1, 147.7, 134.8, 131.2, 124.7, 123.5, 121.7, 120.1, 113.9; *m/z* (ESI+) 323 ([M+H]$^{+,}$ 100%); HRMS (ESI+) found 323.0274 ([M+H]$^+$), $C_{12}H_{11}N_4O_3S_2^+$ requires 323.0267. IR ν_{max} (film) /cm^{-1} 1580, 1350, 1150.

Synthesis of OSM-LO-81

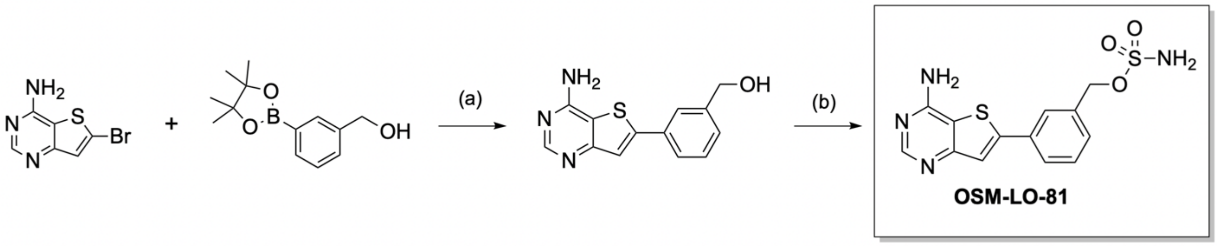

Supplementary Figure 18: Synthetic route to OSM-LO-81. Reagents and conditions: (a) PdCl$_2$(dppf)·DCM, potassium acetate, dioxane, 400 W microwave, 90 °C, 90 min; (b) Sulfamoyl chloride, DMF, rt, 5 h.

(3-(4-Aminothieno[3,2-*d*]pyrimidin-6-yl)phenyl)methanol (12)

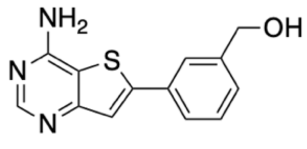

12

Prepared according to General Procedure 1 from: compound **4** (50.0 mg, 0.22 mmol, 1 equiv.), (3-(hydroxymethyl)phenyl)boronic acid (40 mg, 0.27 mmol, 1.2 equiv.), PdCl$_2$(dppf)·DCM (18 mg, 0.065 mmol, 0.1 equiv.), *i*-PrOH (6 mL, 0.5 M) and 1 M aq. K_2CO_3 (1.24 mL, 1.24 mmol) under microwave irradiation at 90 °C for 90 min to give the crude title compound as a dark brown solid; purified by automated flash chromatography on silica (Biotage Isolera, 5 – 20% MeOH in DCM) to give *the title compound* as a brown solid (33.7 mg, 60%). R_F = 0.3 (10% MeOH in DCM); M.P. decomposes at 201 °C (no lit. M.P.); ^{1}H NMR (400 MHz, DMSO-d$_6$) δ 8.37 (s, 1 H), 7.81 – 7.70 (m, 3 H), 7.52 – 7.24 (m, 4 H), 5.33 (apparent t, J = 5.8 Hz, 1 H), 4.59 (d, J = 5.7 Hz, 2 H); ^{13}C NMR (101 MHz, DMSO-d$_6$) δ 160.6, 157.9, 155.2, 149.0, 143.8, 132.6, 129.1, 127.5, 124.5, 124.0, 120.3, 113.2, 62.5; *m/z* (ESI+) 258 ([M+H]$^+$, 100 %); HRMS (ESI+) found 258.0707 ([M+H]$^+$), $C_{13}H_{12}N_3OS^+$ requires 258.0696.

3-(4-Aminothieno[3,2-*d*]pyrimidin-6-yl)benzyl sulfamate (OSM-LO-81)

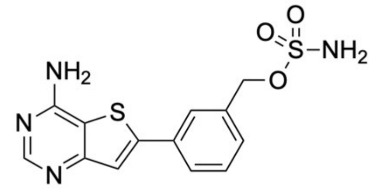

OSM-LO-81

Compound **7** (105 mg, 0.410 mmol, 1 equiv.) was dissolved in DMF (1.2 mL). Sulfamoyl chloride (143 mg, 1.24 mmol, 3.03 equiv) was added, and the mixture was stirred at rt for 5 h. The mixture was poured into a sat. aq. $Na_2S_2O_3$ solution and extracted with EtOAc (2 ×). The combined organic layers were washed with H_2O (2 ×), brine (2 ×), dried ($MgSO_4$), filtered and concentrated under reduced pressure to give the crude product which was purified by automated flash chromatography on silica (Biotage Isolera, 5 – 15% MeOH in DCM) to give *the title compound* as a white solid (41.3 mg, 30%). R_F = 0.4 (10% MeOH in DCM); M.P. decomposes at 240 °C (no lit. M.P.); ^{1}H NMR (400 MHz, DMSO-d$_6$) δ 8.36 (s, 1 H), 7.81 – 7.87 (m, 3 H), 7.68 (s, 2 H), 7.46 – 7.56 (m, 4 H), 5.17 (s, 2 H); ^{13}C NMR (101 MHz, DMSO-d$_6$) δ 160.3, 157.8, 155.1, 148.0, 135.9, 132.9, 129.4, 129.0, 126.1, 125.7, 120.6, 113.2, 69.5; *m/z* (ESI+) 337 ([M+H]$^+$, 100%); HRMS (ESI+) found 337.0430 ([M+H]$^+$), $C_{13}H_{11}N_4O_3S_2^+$ requires 327.0424.

Synthesis of OSM-S-137

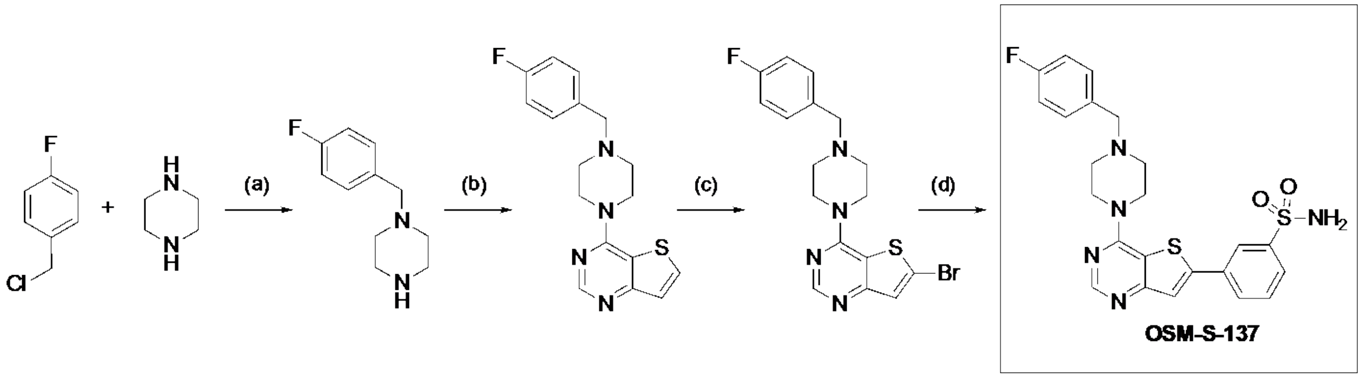

Supplementary Figure 19: Synthetic route to OSM-S-137. Reagents and conditions: (a) THF, reflux, 1 h; (b) compound 2, 80 °C under Ar atmosphere, 19 h; (c) THF, –78 °C, 10 min *then* *n*-BuLi, –78 °C, 40 min *then* Br_2, –78 °C, rt, 1 h; (d) compound **5**, $PdCl_2$(dppf)·DCM, potassium acetate, *i*-PrOH, 70 °C, 15 h under N_2.

1-(4-Fluorobenzyl)piperazine (13)

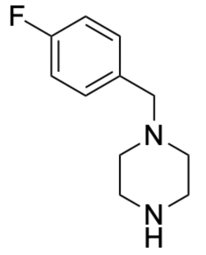

13

Anhydrous piperazine (4.98 g, 57.8 mmol, 6 equiv.) was added to THF (20 mL) and the mixture was heated at reflux until the piperazine was fully dissolved. 4-Fluorobenzyl chloride (1.2 mL, 10.0 mmol, 1 equiv.) was added dropwise and a white precipitate was formed immediately. The reaction mixture was heated at reflux for 1 h and then allowed to cool to rt. The mixture was filtered and washed with THF (100 mL) and then redissolved with EtOAc (100 mL). The combined organic layers were washed with 1 M KOH solution (2 ×), H_2O (2 ×), brine (2 ×), dried ($MgSO_4$), filtered and concentrated under reduced pressure to give a residue that was purified by automated flash chromatography on silica (Biotage Isolera, 0 – 100% MeOH in EtOAc) to give *the title compound* as a white solid (449 mg, 23%). R_F = 0.15 (50% MeOH in EtOAc); ^{1}H NMR (400 MHz, $CDCl_3$) δ 7.29 – 7.23 (m, 2 H), 7.02 – 6.95 (m, 2 H), 3.45 (s, 2 H), 3.18 (br s, 1 H), 2.94 – 2.89 (m, 4 H), 2.44 (s, 4 H); ^{13}C NMR (101 MHz, $CDCl_3$) δ 161.0 (d, *J* = 246.6 Hz), 133.8 (d, *J* = 4.3 Hz), 130.8, 115.2, 62.8, 53.8, 45.8; *m/z* (ESI+) 195.1 ($[M+H]^+$, 100%). The NMR data matched those in the literature (Zhang et al., 2011).

4-(4-(4-Fluorobenzyl)piperazin-1-yl)thieno[3,2-*d*]pyrimidine (14)

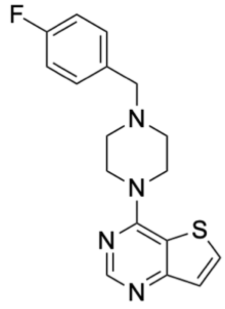

14

Compound **13** (1.97 g, 10.1 mmol, 2 equiv.) was dissolved in EtOH (24 mL) and compound **2** (870 mg, 5.09 mmol, 1 equiv.) was added. The reaction mixture was heated at 80 °C under Ar atmosphere for 19 h. The reaction mixture was cooled to rt and H_2O was added to give a white precipitate. Ethanol was removed under reduced pressure to give a precipitate that was filtered to give *the title compound* as a cream solid (1.4 g, 85%). R_F = 0.18 (70% EtOAc in hexane); M.P. 125 – 126 °C (no lit. M.P.); ^{1}H NMR (400 MHz, $CDCl_3$) δ 8.54 (s, 1 H), 7.70 – 7.68 (d, *J* = 5.6 Hz, 1 H), 7.41 – 7.40 (d, *J* = 5.6 Hz, 1 H), 7.32 – 7.28 (m, 2 H), 7.03 – 6.98 (m, 2 H), 4.02 – 3.97 (m, 4 H), 3.51 (s, 2 H), 2.59 – 2.53 (m, 4 H) ppm; ^{13}C NMR (101 MHz, $CDCl_3$) δ 162.4 (d, *J* = 246.6 Hz), 161.5, 158.1, 154.4, 133.48 (d, *J* = 4.1 Hz), 131.4, 130.66, 125.4, 115.4 (d, *J* = 22.7 Hz), 114.5, 62.2, 53.0, 46.1; *m/z* (ESI+) 329.4 ($[M+H]^+$, 100 %).

6-Bromo-4-(4-(4-fluorobenzyl)piperazin-1-yl)thieno[3,2-*d*]pyrimidine (15)

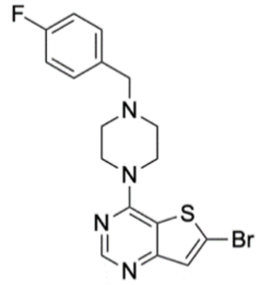

15

Compound **14** (1.04 g, 3.15 mmol, 1 equiv.) was dissolved in dry THF (60 mL) and stirred at – 78 °C under an Ar atmosphere for 20 min. *n*-Butyllithium (1.6 M in hexane hexane (Burchat et al., 1997), 3.1 mL, 5.0 mmol, 1.6 equiv.) was added to the solution at –78 °C. The stirring was continued at –78 °C for 40 min before bromine (0.34 mL, 6.6 mmol, 2.1 equiv.) was added. The reaction was stirred at rt for a further 2 h and quenched with sat. aq. $Na_2S_2O_3$ solution (100 mL). The solvent was removed under reduced pressure. Sat. aq. $Na_2S_2O_3$ solution (20 mL) and EtOAc (20 mL) were added to the residue, and the organic layer was separated. The aqueous phase was extracted with EtOAc (3 ×) and the combined organic layers were washed with H_2O (2 ×), brine (2 ×), dried ($MgSO_4$), filtered and concentrated under reduced pressure to give a brown crude product which was purified by automated flash chromatography on silica (Biotage Isolera, 50 – 100% EtOAc in hexane) to give *the title compound* as an orange solid (514 mg, 40%). R_F = 0.18 (50% EtOAc in hexane); M.P. 91 – 93 °C (no lit. M.P.); ^{1}H NMR (400 MHz, $CDCl_3$) δ 8.50 – 8.48 (m, 1 H), 7.40 (s, 1 H), 7.32 – 7.27 (m, 2 H), 7.03 (d, *J* = 8.6 Hz, 2 H), 3.93 (d, *J* = 4.8 Hz, 4 H), 3.53 – 3.51 (m, 2 H), 2.57 (d, *J* = 4.8 Hz, 4 H) ppm; ^{13}C NMR (101 MHz, $CDCl_3$) δ 162.4 (d, *J* = 246.6 Hz), 161.2, 156.8, 154.8, 133.3 (d, *J* = 4.1 Hz), 130.8, 128.1, 122.8, 116.2, 115.5 (d, *J* = 22.7 Hz), 62.2, 52.9, 46.1. *m/z* (ESI+) 407.0 ($[M+H]^+$, 100 %).

3-(4-(4-(4-Fluorobenzyl)piperazin-1-yl)thieno[3,2-*d*]pyrimidin-6-yl)benzenesulfonamide (OSM-S-137)

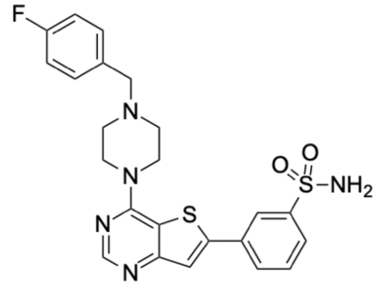

OSM-S-137

Prepared according to General Procedure 1 from: Compound **15** (51 mg, 0.13 mmol, 1 equiv.), compound **5** (38 mg, 0.13 mmol, 1 equiv.) and $PdCl_2$(dppf)·DCM (14 mg, 0.020 mmol, 0.15 equiv.), *i*-PrOH (3 mL) and K_2CO_3 solution (1 M, 0.2 mL, 0.2 mmol, 1.5 equiv.), heated at 70 °C for 15 h under Ar. The reaction was cooled and dissolved in ethyl acetate and water (1:1) (20 mL) and filtered through celite. The aqueous phase was extracted with EtOAc (3 ×) and the combined organic layers were washed with H_2O (2 ×), brine (2 ×), dried ($MgSO_4$), filtered and concentrated under reduced pressure to give *the title compound as* a brown crude product which was purified by automated flash chromatography on silica (Biotage Isolera, 10 – 100% EtOAc in hexane) and repurified by reversed-phase automated flash chromatography on silica (Biotage Isolera, 5 – 100 % MeOH in H_2O) to give *the title compound* as a brown solid (20 mg, 32%). R_F = 0.18 (100% EtOAc); M.P. decomposes at 226 °C (no lit. M.P.); ^{1}H NMR (500 MHz, DMSO-d_6) δ 8.51 (s, 1 H), 8.25 (s, 1 H), 8.16 (d, *J* = 8.1 Hz, 1 H), 7.97 (s, 1 H), 7.89 (d, *J* = 8.2 Hz, 1 H), 7.72 (apparent t, *J* = 7.8 Hz, 1 H), 7.50 (br s, 2 H), 7.38 (dd, *J* = 8.3, 5.6 Hz, 2 H), 7.17 (apparent t, *J* = 8.8 Hz, 2 H), 3.96 (s, 4 H), 3.54 (s, 2 H), 2.55 (s, 4 H) ppm; ^{13}C NMR (126 MHz, DMSO-d_6) δ 161.8 (d, *J* = 246.6 Hz), 161.3, 157.0, 154.5, 146.8, 145.2, 133.9 (d, *J* = 4.1 Hz), 132.9, 130.8, 129.5, 126.5, 123.0, 121.8, 115.0 (d, *J* = 22.7 Hz), 114.9, 113.4, 60.8, 52.3, 45.6; *m/z* (ESI+) 484.1 ($[M+H]^+$, 100 %); HRMS (ESI+) found 485.1306 ($[M+H]^+$), $C_{23}H_{23}FN_5O_2S_2^+$ requires 485.1306.

Synthesis of OSM-LO-87

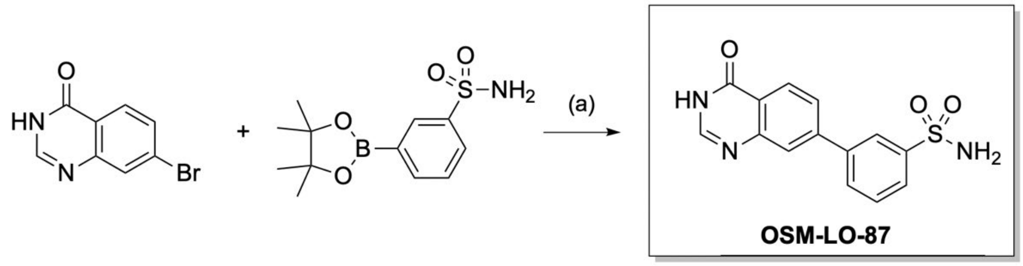

Supplementary Figure 20: Synthetic route to OSM-LO-87. Reagents and conditions: (a) General procedure 1: $PdCl_2$(dppf)·DCM, 1 M aq. K_2CO_3, *i*-PrOH, microwave, 90 °C, 90 min.

3-(4-Oxo-3,4-dihydroquinazolin-7-yl)benzenesulfonamide (OSM-LO-87)

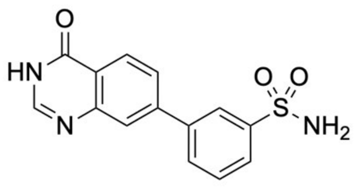

OSM-LO-87

Prepared according to General Procedure 1 from: 7-bromoquinazolin-4(3*H*)-one (60 mg, 0.030 mmol, 1 equiv.), 3-(4,4,5,5-tetramethyl-1,3,2-dioxaborolan-2-yl)benzenesulfonamide (90 mg, 0.030 mmol, 1.22 equiv.) and $PdCl_2$(dppf)·DCM (20 mg, 0.0030 mmol, 0.10 equiv.), *i*-PrOH (2 mL, 0.5 M) and 1 M aq. K_2CO_3 (0.8 mL, 0.8 mmol, 3 equiv.) under microwave irradiation at 90 °C for 1.5 h to give the crude title compound as a brown solid; purified by automated flash chromatography on silica (Biotage Isolera, 5 – 20% MeOH (with 10% MeOH): DCM) to give *the title compound* as a dark brown solid (30 mg, 43%). R_F = 0.25 (10% MeOH in DCM) ; M.P. decomposes at 269 °C; ^{1}H NMR (400 MHz, DMSO-d_6) δ 12.31 (s, 1 H), 8.29 – 8.21 (m, 2 H), 8.17 (s, 1 H), 8.06 (m, 1 H), 7.99 (d, *J* = 1.8 Hz, 1 H), 7.88 (td, J = 7.7, 1.7 Hz, 2 H), 7.73 (t, *J* = 7.8 Hz, 1H), 7.4 (s, 2 H) ppm; ^{13}C NMR (101 MHz, DMSO-d_6) δ 160.8, 149.4, 146.5, 145.0, 144.2, 139.4, 130.4, 129.9, 126.9, 125.4, 125.1, 124.9, 124.3, 122.1; *m/z* (ESI+) 302 ([M+H]$^+$, 100%); HRMS (ESI+) found 302.0600 ([M+H]$^+$), $C_{14}H_{11}N_3O_3S^+$ requires 302.0594.

Synthesis of OSM-LO-88

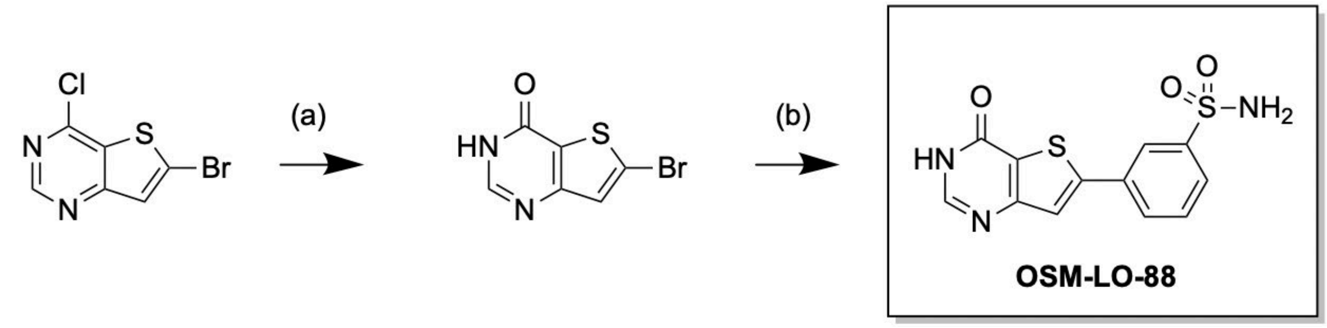

Supplementary Figure 21: Synthetic route to OSM-LO-88. Reagents and conditions: (a) 1 M NaOH, THF, 50 °C, overnight; (b) General procedure 1: $PdCl_2$(dppf)·DCM, 1 M aq. K_2CO_3, *i*-PrOH, microwave, 90 °C, 90 min.

6-Bromothieno[3,2-*d*]pyrimidin-4(3*H*)-one (16)

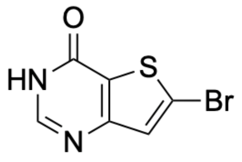

16

To a solution of 6-bromo-4-chlorothieno[3,2-d]pyrimidine (250 mg, 1.0 mmol, 1 equiv.) in THF (5 mL) was added 1 M NaOH (aq) (1.5 mL, 1.5 mmol, 1.5 equiv.). The reaction was heated at 50 °C overnight. The reaction was allowed to cool to rt and 1 M HCl (aq) was added until a neutral pH was achieved. The mixture was concentrated under reduced pressure to give the crude product which was purified by automated flash chromatography on silica (Biotage Isolera, 0 – 15% EtOAc in hexane) to give *the title compound* as a yellow solid (471 mg, 84%). R_F = 0.32 (50% EtOAc in hexane); **^{1}H NMR** (400 MHz, DMSO-d_6) δ 12.64 (s, 1 H), 8.15 (s, 1 H), 7.62 (s, 1 H) ppm; *m/z* (ESI+) 230.0 ([M+H]$^+$, 100%); The NMR data matched those in the literature (De Schutter et al., 2014).

3-(4-Oxo-3,4-dihydrothieno[3,2-*d*]pyrimidin-6-yl)benzenesulfonamide (OSM-LO-88)

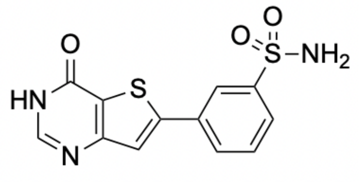

OSM-LO-88

Prepared according to General Procedure 1 from: compound **16** (45 mg, 0.20 mmol, 1 equiv.), 3-(4,4,5,5-tetramethyl-1,3,2-dioxaborolan-2-yl)benzenesulfonamide (67 mg, 0.24 mmol, 1.2 equiv.) and $PdCl_2$(dppf)·DCM (16 mg, 0.020 mmol, 0.10 equiv.), *i*-PrOH (6 mL, 0.5 M) and 1 M aq. K_2CO_3 (0.5 mL, 0.5 mmol, 3 equiv.) under microwave irradiation at 90 °C for 1.5 h to give the crude title compound as a brown solid which purified by automated flash chromatography on silica (Biotage Isolera, 5 – 20% MeOH (with 10% MeOH): DCM) to give *the title compound* as a dark brown solid (24.3 mg, 41%). R_F = 0.23 (5% MeOH in DCM); **^{1}H NMR** (400 MHz, DMSO-d$_6$) δ 8.23 (apparent t, *J* = 1.9 Hz, 1H), 8.20 (s, 1H), 8.12 (apparent dt, *J* = 8.0, 1.2 Hz, 1H), 7.93 (s, 1H), 7.90 – 7.87 (m, 1H), 7.71 (apparent t, *J* = 7.8 Hz, 1H), 7.50 (s, 2H); **^{13}C NMR** (101 MHz, DMSO-d$_6$) δ 158.4 156.9, 148.8, 147.3, 145.3, 133.2, 130.2, 129.4, 126.3, 123.0, 122.4; *m/z* (ESI+) 308 ([M+H]$^+$, 100%); **HRMS** (ESI+) found 308.0168 ([M+H]$^+$), $C_{12}H_9N_3O_3S_2^+$ requires 308.0158.

Synthesis of Asn-OSM-S-106 adduct (UCL)

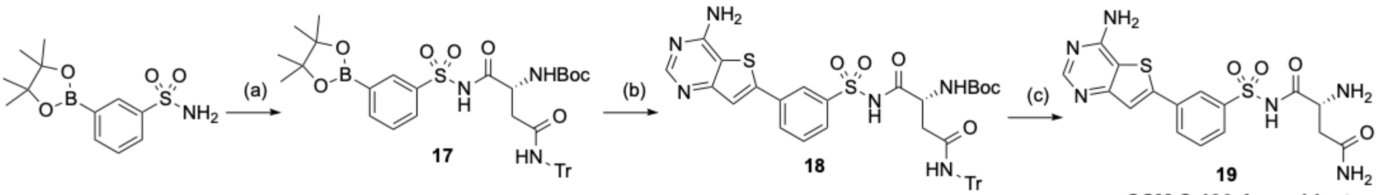

OSM-S-106-Asn adduct

Supplementary Figure 22: Synthetic route of Asn-OSM-S-106 adduct synthesis in UCL. Reagents and conditions: (a) Nα-Boc-Nγ-trityl-L-asparagine, DMAP, DCM, EDCl, 50 °C, 3 d; (b) 6-bromothieno[3,2-*d*] pyrimidin-4-amine, $PdCl_2$(dppf)•DCM, KOAc, dioxane, 400 W microwave, 90 °C, 90 min; (c) 4 M HCl, dioxane, 50 °C, overnight.

tert-Butyl (*R*)-(1,4-dioxo-1-((3-(4,4,5,5-tetramethyl-1,3,2-dioxaborolan-2-yl)phenyl) sulfonamido)-4-(tritylamino)butan-2-yl)carbamate (17)

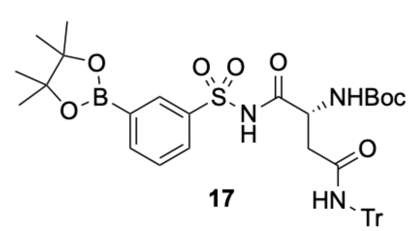

Compound **5** (250 mg, 0.883 mmol, 1 equiv.), Nα-Boc-Nγ-trityl-L-asparagine (625 mg, 1.32 mmol, 1.49 equiv.) and DMAP (479 mg, 3.92 mmol, 4.44 equiv.) were dissolved in DCM (9.0 mL, 0.1 M). EDCI (0.50 mL, 2.82 mmol, 3.2 equiv.) was added and the reaction was stirred at 50 °C for 3 d. The reaction mixture was diluted with DCM and poured into 1 M HCl (10 mL). The aqueous phase was extracted with DCM (2 ×) and the combined organic layers were washed with brine, dried ($MgSO_4$), and concentrated to give the crude material as a yellow residue (846 mg) that was used in the next reaction without further purification. *m/z* (ESI+) 762.3 ([M+Na]$^+$, 100%).

tert-Butyl (*R*)-(1-((3-(4-aminothieno[3,2-d] pyrimidin-6-yl) phenyl)sulfonamido)-1,4-dioxo-4-(tritylamino)butan-2-yl)carbamate (18)

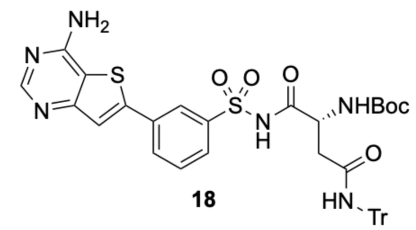

Prepared according to General Procedure 1 from: Compound **4** (180 mg, 0.782 mmol, 1 equiv.), crude **11** (653 mg, *ca.* 0.883 mmol, *ca.* 1.13 equiv.), $PdCl_2$(dppf)•DCM (64 mg, 0.078 mmol, 0.1 equiv.), *i*-PrOH (16.0 mL, 0.05 M) and 1 M aq. K_2CO_3 (2.4 mL, 2.40 mmol) under microwave irradiation at 90 °C for 90 min; purified by flash chromatography (5–10% MeOH (10% NH_4OH) in DCM), then repurified by reverse phase chromatography (5–100% MeOH in H_2O) to give *the title compound* as a light brown solid (207 mg, 35%); used in the next reaction without further purification. ^{1}H NMR (500 MHz, methanol-d$_4$) δ 8.37 (s,

1 H), 8.36 – 8.32 (m, 1 H), 8.01 – 7.86 (m, 2 H), 7.67 (s, 1 H), 7.60 – 7.51 (m, 1 H), 7.11 – 7.31 (m, 19 H), 4.31 – 4.41 (m, 1 H), 2.80 (s, 2 H), 2.40 – 2.54 (m, 1 H), 1.37 (s, 9 H) ppm.

(*R*)-2-amino-*N*1-((3-(4-aminothieno[3,2-*d*]pyrimidin-6-yl)phenyl)sulfonyl)succinamide (19)

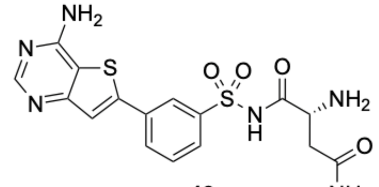

19

OSM-S-106-Asn adduct

Compound **12** (200 mg, 0.262 mmol, 1 equiv.) was dissolved in dioxane (2.2 mL, 0.12 M) and cooled to 0 °C. 4 M HCl in dioxane (1.3 mL, 5.2 mmol, 20 equiv.) was added dropwise and the reaction mixture was allowed to stir at 50 °C overnight. The volatiles were removed, and the crude material was purified by reverse phase chromatography (5 – 100% MeOH in H_2O). This material was then repurified by flash chromatography (5–40% 10% NH_4OH/ MeOH in DCM to give *the title compound* a white solid (11 mg, 10 %). ^{1}H NMR (400 MHz, DMSO-d$_6$) δ 8.38 (s, 1 H), 8.18 (s, 1 H), 7.96 (d, *J* = 7.6 Hz, 1 H), 7.84 (d, *J* = 7.6 Hz, 1 H), 7.80 (s, 1 H), 7.71 (s, 3 H), 7.60 – 7.51 (m, 2 H), 7.48 (s, 2 H), 7.10 (s, 1 H), 3.67 (dd, *J* = 9.3, 3.0 Hz, 1 H), 2.79 (dd, *J* = 17.1, 3.2 Hz, 1 H), 2.39 (dd, *J* = 16.9, 9.3 Hz, 1 H) ppm; ^{13}C NMR (500 MHz, DMSO-d$_6$) δ 171.7, 160.5, 158.0, 155.3, 148.0, 146.4, 132.2, 128.9, 127.9, 127.8, 124.6, 120.9, 113.4, 51.6, 35.3 ppm. *m/z* (ESI) 420.8 ([M+H]$^+$, 100%); HRMS (ESI+) found 421.0786 ([M+H]$^+$), $C_{16}H_{17}N_6O_4S_2$ requires 421.0747.

Alternative synthesis of Asn-OSM-S-106 adduct (TCGLS)

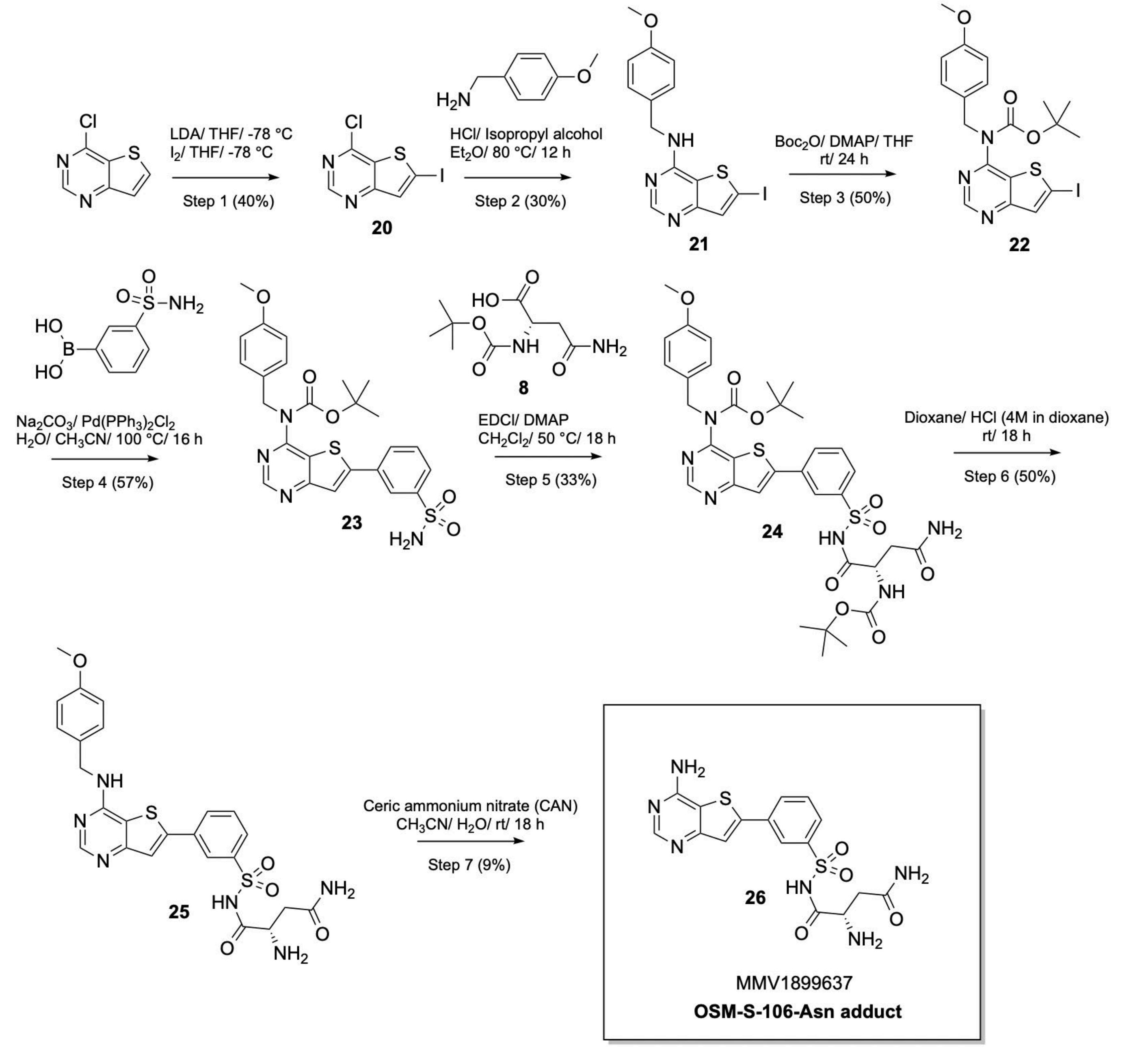

Supplementary Figure 23: Synthetic route of Asn-OSM-S-106 adduct synthesis in TCGLS. Reagents and conditions are shown above**.** Compound 26 (also named as MMV1899637/ Asn-OSM-S-106 adduct) is identical in structure to compound 19 and shares the same data.

4-Chloro-6-iodothieno[3,2-*d*]pyrimidine (20):

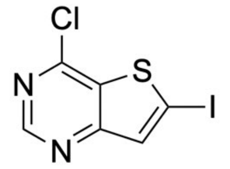

20

To a cooled solution of LDA (2 M in hexane, 10.7 mL, 21.4 mmol) in THF (30 mL) at -78 °C was added solution of 4-chlorothieno[3,2-*d*]pyrimidine (3 g, 17.6 mmol) in THF (20 mL). After stirring at -78 °C for 20 mins, solution of Iodine (5.42 g, 21.4 mmol) in THF (20 mL) was added. The reaction mixture was warmed to room temperature and stirred at room temperature for 30 mins. The reaction was quenched by adding 200 mL of chloroform. The reaction mixture was partitioned between chloroform and H_2O. The organic layer was collected, and the aqueous layer was extracted with 100 ml of chloroform. The combined organic layers were washed with of aqueous sodium thiosulfate twice and brine. The organic layer was dried over Na_2SO_4 and concentrated and purified by flash chromatography (20% EtOAc in hexane) to

give *the title compound* a white solid (2.1 g, 40% yield). ^{1}H NMR (400 MHz, DMSO) δ 8.96 (s, 1 H), 8.13 (s, 1 H) ppm; *m/z* (ESI) 297.06 ([M+H]$^+$, 100%). The NMR data matched those in the literature (Ni et al., 2011).

6-Iodo-N-(4-methoxybenzyl)thieno[3,2-*d*]pyrimidin-4-amine (21):

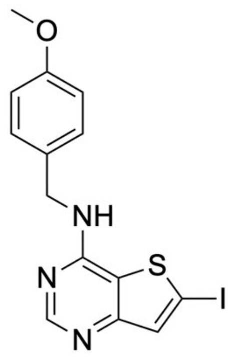

21

To the stirred solution of compound **20** (1.0 g, 3.4 mmol) in 2-propanol (12 mL) in a sealed tube was added compound (4-methoxyphenyl)methanamine (926 mg, 6.75 mmol) followed by HCl (2 mL, 2 M in Et_2O). The reaction mixture was heated at 80°C for 12 h. The reaction mixture was diluted with EtOAc and washed with saturated $NaHCO_3$ solution, organic extracts concentrated in vacuo to obtain a crude residue, which was purified by flash column chromatography (elution with 30 – 40% EtOAc in hexane) to *give the title compound* a white solid (400 mg, 30% yield). *m/z* (ESI) 398.2 ([M+H]$^+$, 100%). ^{1}H NMR (400 MHz, $CDCl_3$) δ 8.54 (s, 1 H), 7.6 (s, 1 H), 7.29 (d, *J* = 8.4 Hz, 2 H), 6.88 (d, *J* = 8.5 Hz, 2 H), 4.97 (s, 1 H), 4.75 (s, 2 H), 3.80 (s, 3 H). The NMR data matched those in the literature (Cai, 2007).

tert-Butyl (6-iodothieno[3,2-*d*]pyrimidin-4-yl)(4-methoxybenzyl)carbamate (22):

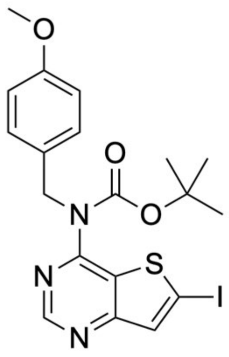

22

To the stirred solution of compound **21** (1.87 g, 4.72 mmol) in dry THF (20 mL), DMAP (633 mg, 5.18 mmol) was added at room temperature, the reaction mixture was stirred for 5 min at this temperature. To the reaction mixture was added Boc_2O (1.62 g, 7.07 mmol) and the reaction mixture was allowed to stir at rt for 24 h. The reaction mixture was partitioned between EtOAc and H_2O, organic extracts concentrated in vacuo to obtain a crude residue, which was purified by silica column chromatography (elution with 5 – 10% EtOAc in hexane) to *give the title compound* as a white solid (1.16 g, 50% yield). *m/z* (ESI) 497.5 ([M+H]$^+$, 100%); ^{1}H NMR (400 MHz, DMSO) δ 8.92 (s, 1 H), 7.90 (s, 1 H), 7.20 (d, *J* = 8.4 Hz, 2 H), 6.85 (d, *J* = 8.4 Hz, 2 H), 5.08 (s, 2 H), 3.69 (s, 3 H), 1.40 (s, 9 H).

tert-Butyl (4-methoxybenzyl)(6-(3-sulfamoylphenyl)thieno[3,2-*d*]pyrimidin-4-yl)carbamate (23):

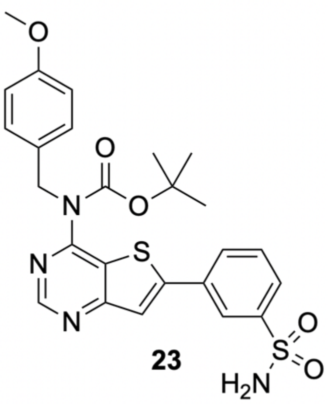

23

To a solution of compound 22 (750 mg, 1.5 mmol) and compound (3-sulfamoylphenyl)boronic acid (333 mg, 1.66 mmol) in MeCN (5 mL) in a sealed tube was added aqueous solution of Na2CO3 (2 M, 2.62 mL, 4.52 mmol) and the reaction mixture was purged with Ar for 20 mins. To the reaction mixture was added bis(triphenylphosphine)palladium(II) dichloride (105 mg, 0.151 mmol) and again purged with Ar for 5 mins. The reaction mixture was heated at 100 °C for 16 h. The reaction mixture was cooled to rt and filtered through the bed of celite, washed and extracted with EtOAc (2 x 20 mL). The combined organic layer was washed with brine, dried over over Na_2SO_4 and concentrated under reduced pressure to get crude which was purified by flash chromatography (50% EtOAc in hexane) to *give the title compound* a light brown solid (450 mg, 57% yield). *m/z* (ESI) 527 ([M+H]$^+$, 100%).

tert-Butyl (6-(3-(*N*-((*tert*-butoxycarbonyl)-*L*-asparaginyl)sulfamoyl)phenyl)thieno[3,2-*d*]pyrimidin-4-yl)(4-methoxybenzyl)carbamate (24):

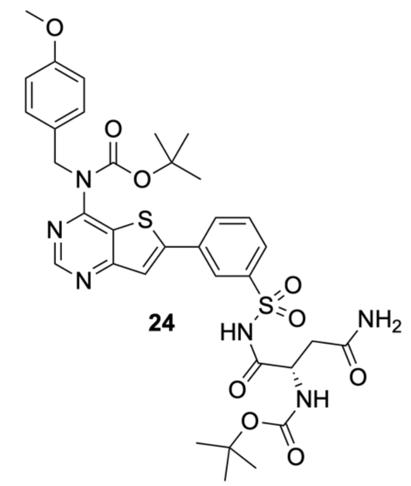

24

To a solution of compound (tert-butoxycarbonyl)-L-asparagine (595 mg, 2.56 mmol) in DCM (10 mL) was added EDCI (492 mg, 2.57 mmol), DMAP (627 mg, 5.13 mmol) and compound 23 (450 mg, 0.86 mmol) (10 mL) at rt and the reaction mixture was stirred at 50 °C for 18 h. The reaction mixture was concentrated under reduced pressure and crude compound was diluted with EtOAc and washed with H_2O and brine solution, dried over Na_2SO_4 and concentrated under reduced pressure to get crude which was purified by column chromatography (10% MeOH in DCM) to *give the title compound* as a light brown gummy solid (210 mg, 33% yield). *m/z* (ESI) 741.5 ($[M+H]^+$, 100%);

tert-Butyl (6-(3-(*N*-(*L*-asparaginyl)sulfamoyl)phenyl)thieno[3,2-*d*]pyrimidin-4-yl)(4-methoxybenzyl)carbamate (25):

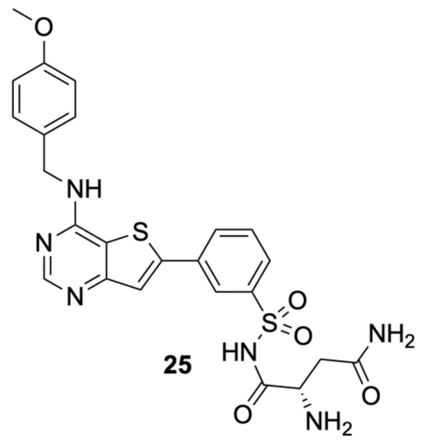

25

To a cooled solution of compound 24 (900 mg, 1.2 mmol) in dioxane (10 mL) at 0 °C was added 4 M HCl in dioxane (6 mL) dropwise. The reaction mixture was allowed to stir at rt for 18 h. The volatiles were removed, and the resultant residue was neutralized with NH_4OH, extracted with 10% MeOH in DCM. The combined organic layer was concentrated to *give the title compound* as a crude solid (330 mg, crude compound, 69% by LCMS) which was used of the next step as such. *m/z* (ESI) 541.5 ($[M+H]^+$, 100%);

(*S*)-2-amino-N^1-((3-(4-aminothieno[3,2-*d*]pyrimidin-6-yl)phenyl)sulfonyl)succinamide (26):

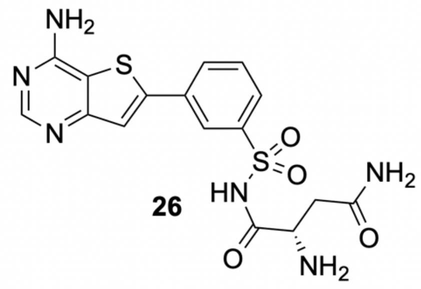

26

MMV1899637

OSM-S-106-Asn adduct

Compound 26 (also named as MMV1899637/ Asn-OSM-S-106 adduct) is identical in structure to compound 19 and shares the same data.

Synthesis of Asn-AMS (via General information 2)

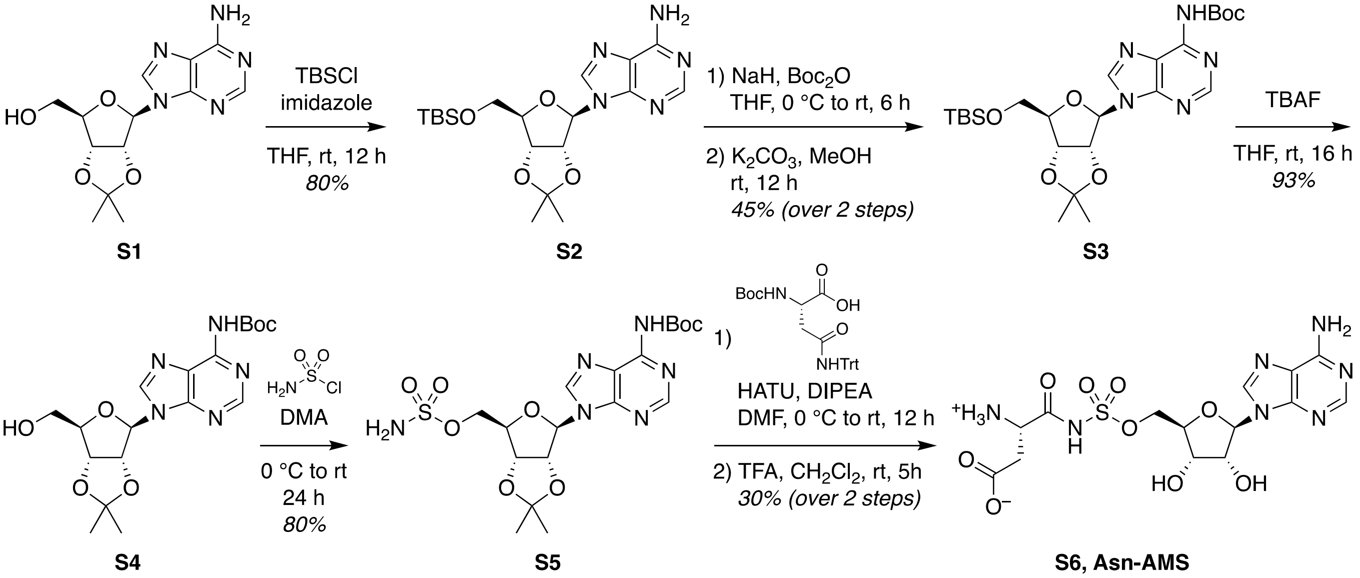

Supplementary Figure 24. Synthetic route of Asn-AMS. Reagents and conditions are shown above.

9-((3*aR*,4*R*,6*R*,6*aR*)-6-(((*tert*-Butyldimethylsilyl)oxy)methyl)-2,2-dimethyltetrahydrofuro[3,4-*d*][1,3]dioxol-4-yl)-9*H*-purin-6-amine (S2)

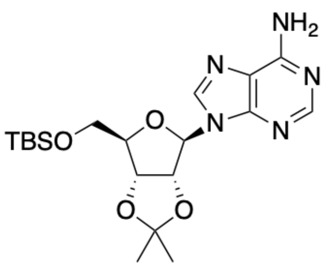

In a 250-mL round bottom flask, 2´,3´-*O*-isopropylideneadenosine **S1** (1 g, 3.25 mmol, 1.0 equiv) was dissolved in THF (20 mL) at rt, followed by addition of TBSCl (0.74 g, 4.88 mmol, 1.5 equiv) and imidazole (0.44 g, 6.5 mmol, 2.0 equiv). After 5 h, MeOH (2 mL) was added to quench excess TBSCl and the mixture was concentrated by rotary evaporation. The residue was diluted with EtOAc, washed with sat. aq. $NaHCO_3$ and brine, dried (Na_2SO_4), filtered, and concentrated by rotary evaporation. Purification by silica flash chromatography (50% EtOAc in hexane) to give *the title compound* as a white solid (1.1 g, 80%). Analytical data agreed with those reported previously (Ishikawa and Kakeya, 2014).

tert-Butyl (9-((3*aR*,4*R*,6*R*,6*aR*)-6-(((*tert*-butyldimethylsilyl)oxy)methyl)-2,2-dimethyltetrahydrofuro[3,4-*d*][1,3]dioxol-4-yl)-9*H*-purin-6-yl)carbamate (S3)

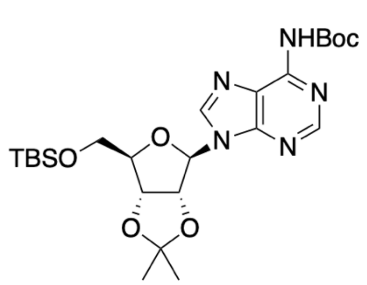

In a 250-mL round bottom flask, 5´-*O*-TBS adenosine **S2** (1 g, 2.37 mmol) was dissolved in THF (15 mL) and cooled to 0 °C, followed by addition of NaH (60% suspension in mineral oil, 164 mg, 3.55 mmol, 1.50 equiv). Once evolution of gas (H_2) had ceased, a solution of Boc_2O (815 µL, 3.55 mmol, 1.50 equiv) in THF (15 mL) was added with vigorous stirring at 0 °C. The reaction mixture was warmed to rt and stirred for 12 h. The reaction mixture was diluted with EtOAc and washed with sat. aq. $NaHCO_3$, then the aq layer was back-extracted with EtOAc. The combined organic extracts were washed with brine, dried (Na_2SO_4), and concentrated by rotary evaporation to afford a mixture of mono- and bis-Boc protected adenosines. The crude product was dissolved in MeOH (30 mL) at rt and K_2CO_3 (624 mg, 4.52 mmol, 2.00 equiv) was added. After 12 h, the reaction mixture was concentrated by rotary evaporation. The residue was extracted with EtOAc, washed with brine, dried (Na_2SO_4), and concentrated by rotary evaporation. Purification by silica flash chromatography (33% EtOAc in hexane) to *give the title*

compound as a white powder (480 mg, 40% over two steps). Analytical data agreed with those reported previously (Ishikawa and Kakeya, 2014).

tert-Butyl (9-((3*aR*,4*R*,6*R*,6*aR*)-6-(((*tert*-butyldimethylsilyl)oxy)methyl)-2,2-dimethyltetrahydrofuro[3,4-*d*][1,3]dioxol-4-yl)-9*H*-purin-6-yl)carbamate (S4)

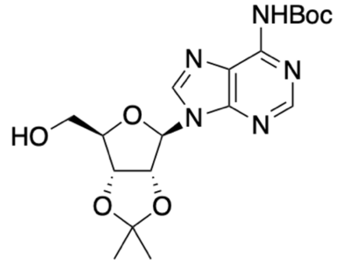

In a 250-mL round bottomed flask 5´-*O*-TBS adenosine **S3** (400 mg, 0.76 mmol) was dissolved in THF (10 mL) at rt, and TBAF (1 M in THF, 1.5 mL, 1.53 mmol, 2 equiv) was added. After 12 h, the reaction mixture was extracted with EtOAc, washed with brine, dried (Na_2SO_4), and concentrated by rotary evaporation. Purification by silica flash chromatography (75% EtOAc in hexane) to *give the title compound* as a white powder (287 mg, 93%). Analytical data agreed with those reported previously (Ishikawa and Kakeya, 2014).

tert-Butyl (9-((3*aR*,4*R*,6*R*,6*aR*)-2,2-dimethyl-6-((sulfamoylamino)methyl)tetrahydrofuro[3,4-*d*][1,3]dioxol-4-yl)-9*H*-purin-6-yl)carbamate (S5)

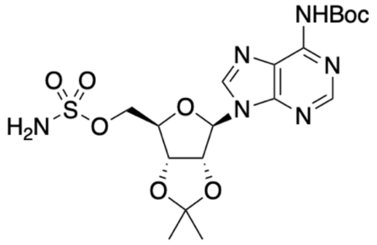

In a 50-mL round bottomed flask, protected adenosine **S4** (200 mg, 0.5 mmol, 1.00 equiv) was dissolved in DMA (10 mL) and cooled to 0 °C, followed by addition of sulfamoyl chloride (85 mg, 0.75 mmol, 1.5 equiv). The mixture was warmed to rt. After 12 h, sat. aq $NaHCO_3$ was added and the resulting mixture was extracted with EtOAc, washed with brine, dried (Na_2SO_4), and concentrated by rotary evaporation. Purification by silica flash chromatography (75% EtOAc in hexane) to *give the title compound* as a white powder (194 mg, 80%). Analytical data agreed with those reported previously (Ishikawa and Kakeya, 2014).

(*S*)-2-Amino-*N*1-(*N*-(((2*R*,3*S*,4*R*,5*R*)-5-(6-amino-9*H*-purin-9-yl)-3,4-dihydroxytetrahydrofuran-2-yl)-methyl)sulfamoyl)succinamide (S6, Asn-AMS)

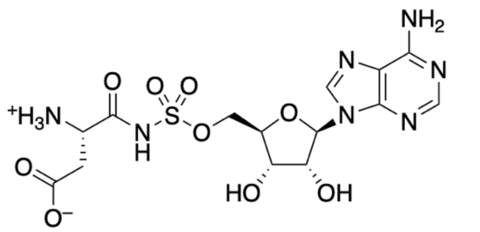

In a 10-mL round bottomed flask, protected AMS **S5** (80 mg, 0.016 mmol, 1.00 equiv) was dissolved in DMF (5 mL) and cooled to 0 °C, followed by addition of Boc-Asn(Trt)-OH (78 mg, 0.016 mmol, 1.00 equiv), HATU (62.5 mg, 0.016 mmol, 1.00 equiv) and DIPEA (86 µL, 0.048 mmol, 3.00 equiv). The reaction mixture was warmed to rt. After 12 h, the reaction mixture was extracted with EtOAc, washed with brine, dried (Na_2SO_4), and concentrated by rotary evaporation to afford the protected Asn-AMS intermediate. The crude product was dissolved in DCM (5 mL) at rt and TFA (1 mL) was added. After 5 h, the mixture was concentrated by rotary evaporation. Purification by reverse-phase HPLC (t_{ret} = 5.3 min) and lyophilization to *give the title compound* as a a clear colourless white solid (26 mg, 30% over two steps). HPLC (of 5–95% CH_3CN in 0.1% aq TFA): t_{ret} = 5.3 min, λ_{max} = 202, 240 nm; ^{1}H-NMR (600 MHz, methanol-d$_4$): δ 8.64 (s, 1H), 8.33 (s, 1H), 6.12 (d, *J* = 5.2 Hz, 1 H), 4.62 (t, *J* = 5.1 Hz, 1 H), 4.36 (m, 4 H), 3.96 (dd, *J* = 8.6, 3.6 Hz, 1 H), 2.97 (dd, *J* = 17.0, 3.6 Hz, 1 H), 2.79 (dd, *J* = 17.0, 8.6 Hz, 1 H); ^{13}C-NMR (151 MHz, methanol-d$_4$): δ 174.6, 174.2, 153.8, 150.3, 148.5, 142.9, 120.1, 89.8, 84.6, 76.5, 72.0, 69.1, 53.8, 35.6; MS (ESI) *m/z* calcd for $C_{14}H_{22}N_9O_7S$ ([M+H]$^+$) 460.95; found 460.97

Supplementary References

Abdelsamie, A.S., C.J. van Koppen, E. Bey, M. Salah, C. Börger, L. Siebenbürger, M.W. Laschke, M.D. Menger, and M. Frotscher. 2017. Treatment of estrogen-dependent diseases: Design, synthesis and profiling of a selective 17β-HSD1 inhibitor with sub-nanomolar IC(50) for a proof-of-principle study. *European journal of medicinal chemistry*. 127:944-957.

Burchat, A.F., J.M. Chong, and N. Nielsen. 1997. Titration of alkyllithiums with a simple reagent to a blue endpoint. *Journal of Organometallic Chemistry*. 542:281-283.

Cai, S.X.D., John A.;Kemnitzer, William E.;Sirisoma, Nilantha Sudath. 2007. N-arylalkyl-thienopyrimidin-4-amines and analogs as activators of caspases and inducers of apoptosis and the use thereof. Vol. US2007/99941. I.P. INC, editor. Cytovia, Inc., US.

De Schutter, J.W., J. Park, C.Y. Leung, P. Gormley, Y.-S. Lin, Z. Hu, A.M. Berghuis, J. Poirier, and Y.S. Tsantrizos. 2014. Multistage screening reveals chameleon ligands of the human farnesyl pyrophosphate synthase: Implications to srug discovery for neurodegenerative diseases. *Journal of medicinal chemistry*. 57:5764-5776.

Eiler, S., A. Dock-Bregeon, L. Moulinier, J.C. Thierry, and D. Moras. 1999. Synthesis of aspartyl-tRNA(Asp) in *Escherichia coli* - a snapshot of the second step. *Embo J*. 18:6532-6541.

Ham, Y.J., D.-H. Lee, H.G. Choi, J.-M. Hah, and T. Sim. 2010. The efficient one-step chlorination of methylsulfanyl group on pyrimidine ring system with sulfuryl chloride. *Tetrahedron Letters*. 51:4609-4611.

Ishikawa, F., and H. Kakeya. 2014. Specific enrichment of nonribosomal peptide synthetase module by an affinity probe for adenylation domains. *Bioorg Med Chem Lett*. 24:865-869.

Liang, R., S. Li, R. Wang, L. Lu, and F. Li. 2017. N-Methylation of amines with methanol catalyzed by a Cp*Ir complex bearing a functional 2,2'-bibenzimidazole ligand. *Org Lett*. 19:5790-5793.

Ni, Y., A. Gopalsamy, D. Cole, Y. Hu, R. Denny, M. Ipek, J. Liu, J. Lee, J.P. Hall, M. Luong, J.B. Telliez, and L.L. Lin. 2011. Identification and SAR of a new series of thieno[3,2-d]pyrimidines as Tpl2 kinase inhibitors. *Bioorg Med Chem Lett*. 21:5952-5956.

Schmitt, E., L. Moulinier, S. Fujiwara, T. Imanaka, J.C. Thierry, and D. Moras. 1998. Crystal structure of aspartyl-tRNA synthetase from *Pyrococcus kodakaraensis* KOD: archaeon specificity and catalytic mechanism of adenylate formation. *Embo J*. 17:5227-5237.

Song, Y.-H. 2007. A facile synthesis of new 4-(phenylamino)thieno[3,2-d]pyrimidines using 3-aminothiophene-2-carboxamide. *Heterocyclic Communications*. 13:33-34.

Wang, J., M. Su, T. Li, A. Gao, W. Yang, L. Sheng, Y. Zang, J. Li, and H. Liu. 2017. Design, synthesis and biological evaluation of thienopyrimidine hydroxamic acid based derivatives as structurally novel histone deacetylase (HDAC) inhibitors. *European journal of medicinal chemistry*. 128:293-299.

Woodring, J.L., G. Patel, J. Erath, R. Behera, P.J. Lee, S.E. Leed, A. Rodriguez, R.J. Sciotti, K. Mensa-Wilmot, and M.P. Pollastri. 2015. Evaluation of aromatic 6-substituted thienopyrimidines as scaffolds against parasites that cause trypanosomiasis, leishmaniasis, and malaria. *MedChemComm*. 6:339-346.

Zhang, C., C. Tan, X. Zu, X. Zhai, F. Liu, B. Chu, X. Ma, Y. Chen, P. Gong, and Y. Jiang. 2011. Exploration of (S)-3-aminopyrrolidine as a potentially interesting scaffold for discovery of novel Abl and PI3K dual inhibitors. *European Journal of Medicinal Chemistry*. 46:1404-1414.
