# Supplementary material for: Reaction hijacking inhibition of Plasmodium falciparum asparagine tRNA synthetase
Source: Nat Commun. 2024 Jan 31;15:937. doi: 10.1038/s41467-024-45224-z (PMC10831071; doi:10.1038/s41467-024-45224-z)
Supplement: Supplementary file 3 — Description of Additional Supplementary Files [file 41467_2024_45224_MOESM3_ESM.docx]

**Description of Additional Supplementary Files:**

**Supplementary Data 1:**

Description: Characterisation of compounds synthesized in this work.

**Supplementary Data 2:**

Description: Whole-genome sequence analysis of amplification events encompassing the dihydroorotate dehydrogenase (dhodh) locus in DSM265-selected parasites.

**Supplementary Data 3:**

Description: Per-sample SNV and INDEL calls in core genes in the OSM-S-106-selected parasite samples that were whole genome sequenced.

**Supplementary Data 4:**

Description: CNV analyses in Dd2 and 3D7 parasites selected with OSM-S-106.
