# Supplementary material for: Reaction hijacking inhibition of Plasmodium falciparum asparagine tRNA synthetase
Source: Nat Commun. 2024 Jan 31;15:937. doi: 10.1038/s41467-024-45224-z (PMC10831071; doi:10.1038/s41467-024-45224-z)

**Nature Communications**

***Supplementary data 1***

**Spectra for compounds synthesized in this work:**

**OSM-S-106:**

**OSM-E-32:**

**OSM-S-488:**

**OSM-LO-80:**

**OSM-LO-81:**

**OSM-S-137:**

**
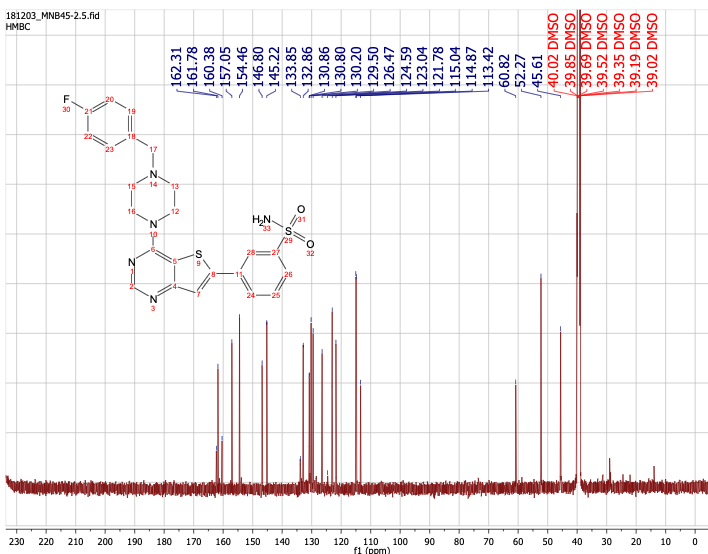
**

**OSM-LO-87:**

**OSM-LO-88:**

**OSM-S-106-Asn adduct:**

**
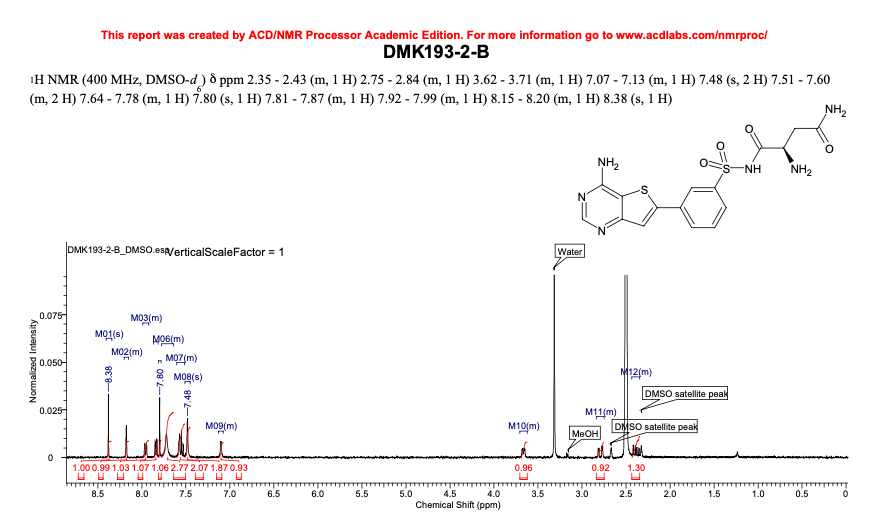
**

**
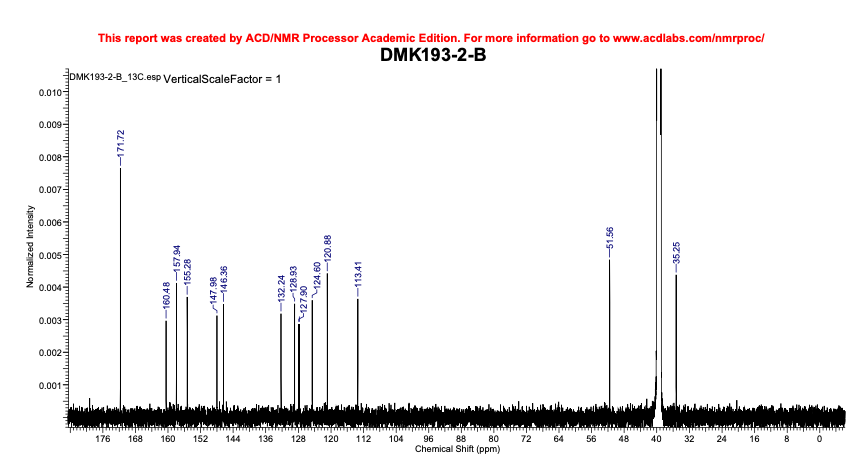
**

**Asn-AMS adduct:**


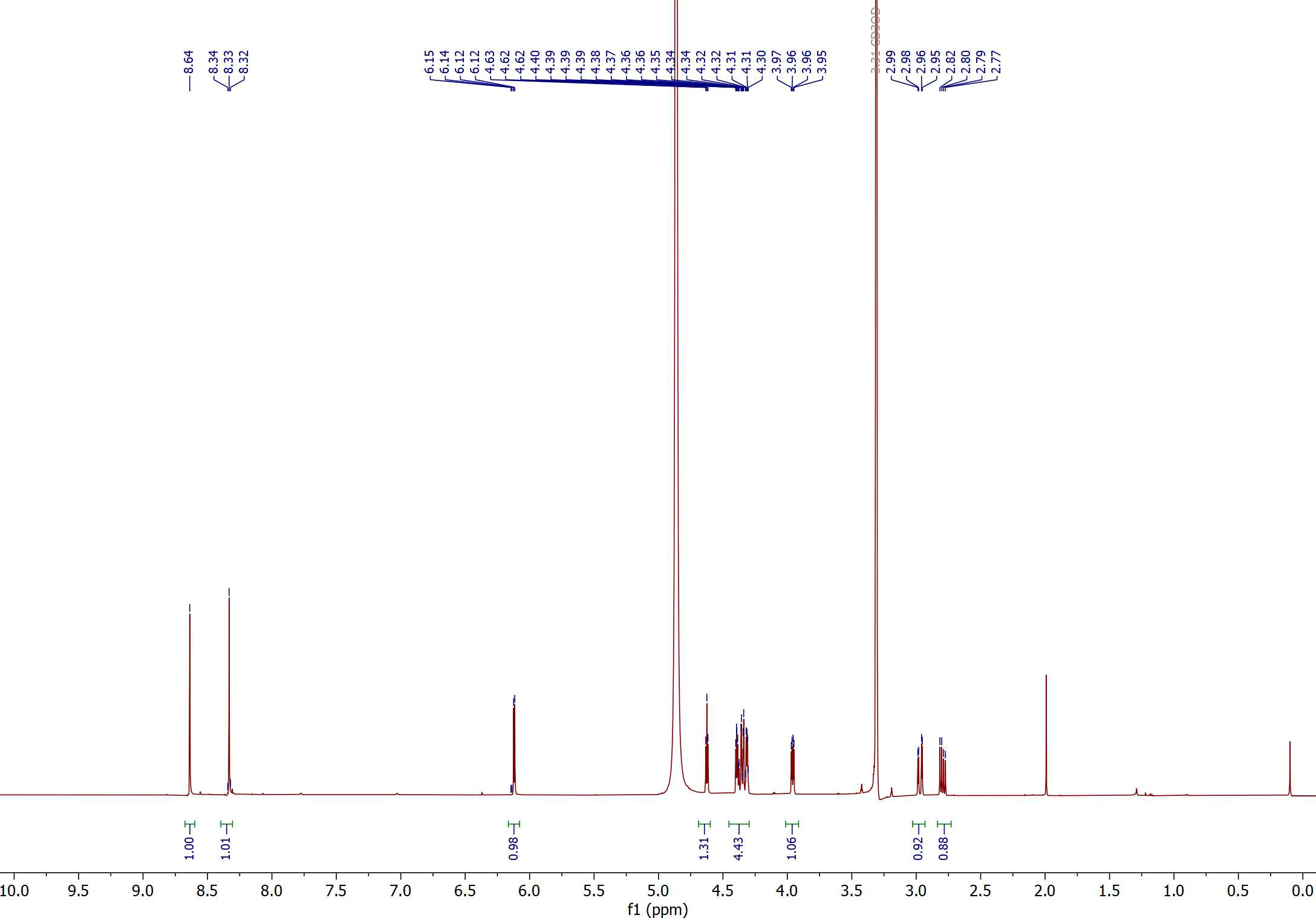


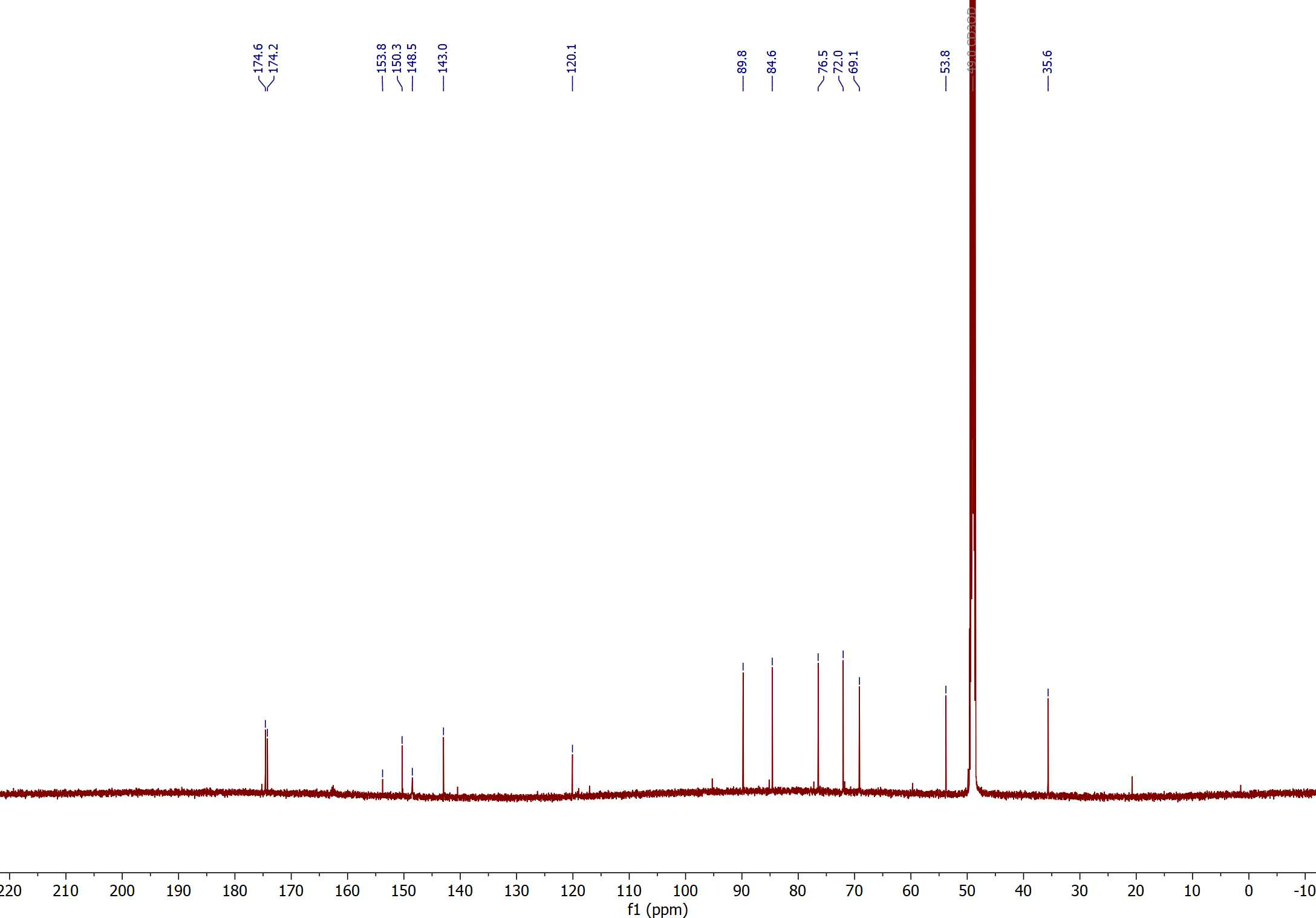


**Asn-OSM-S-106：**


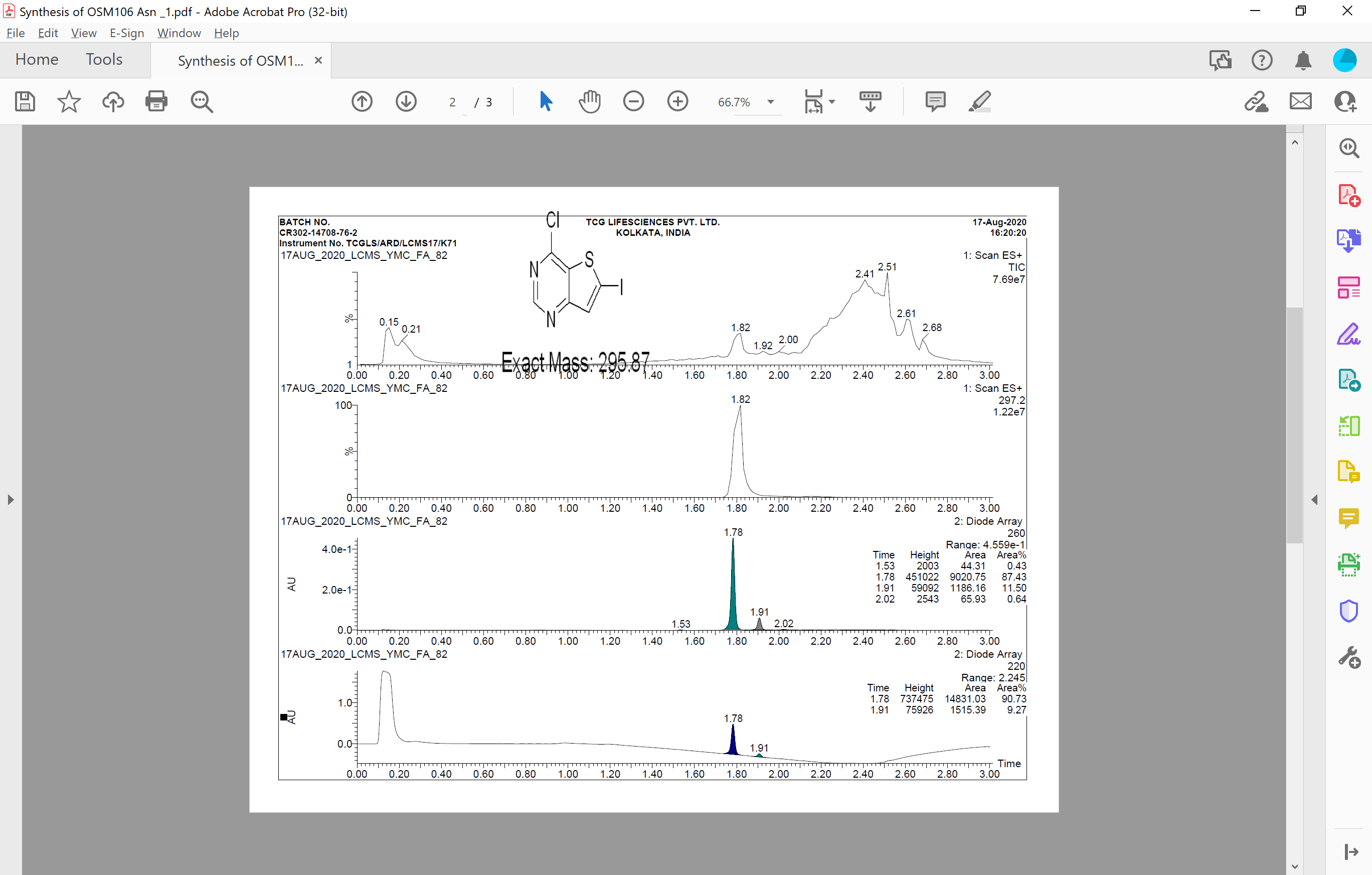

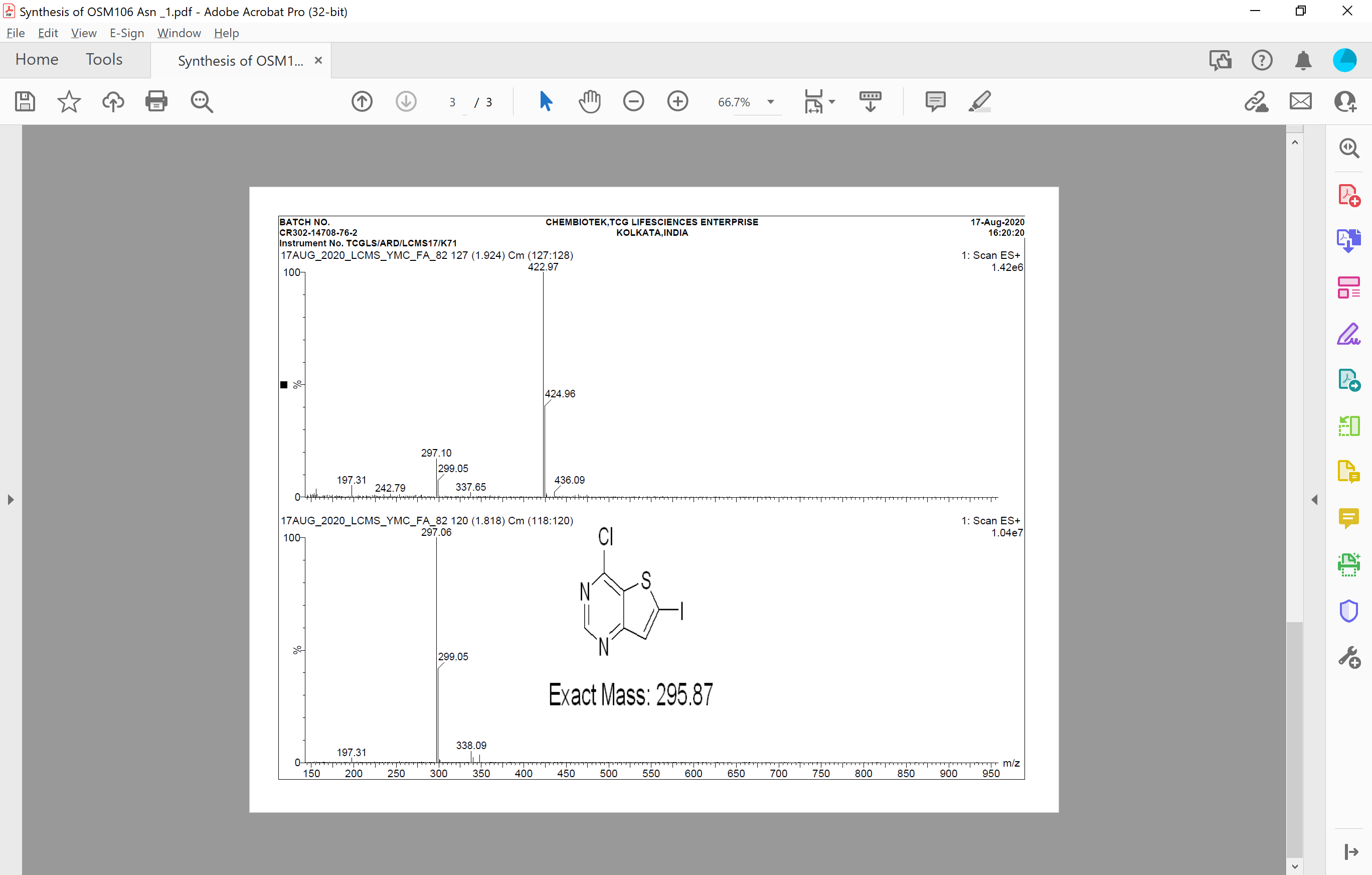

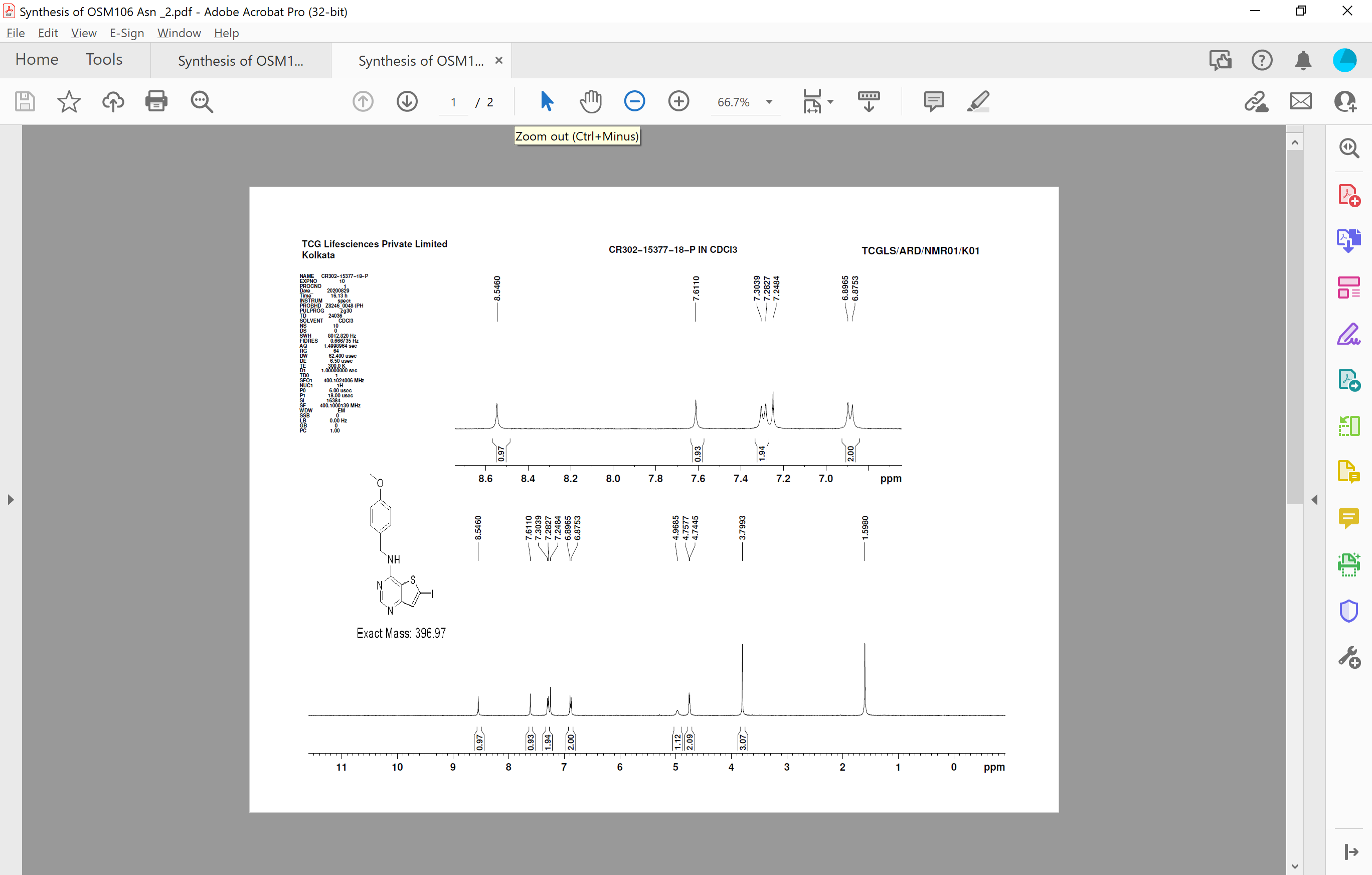

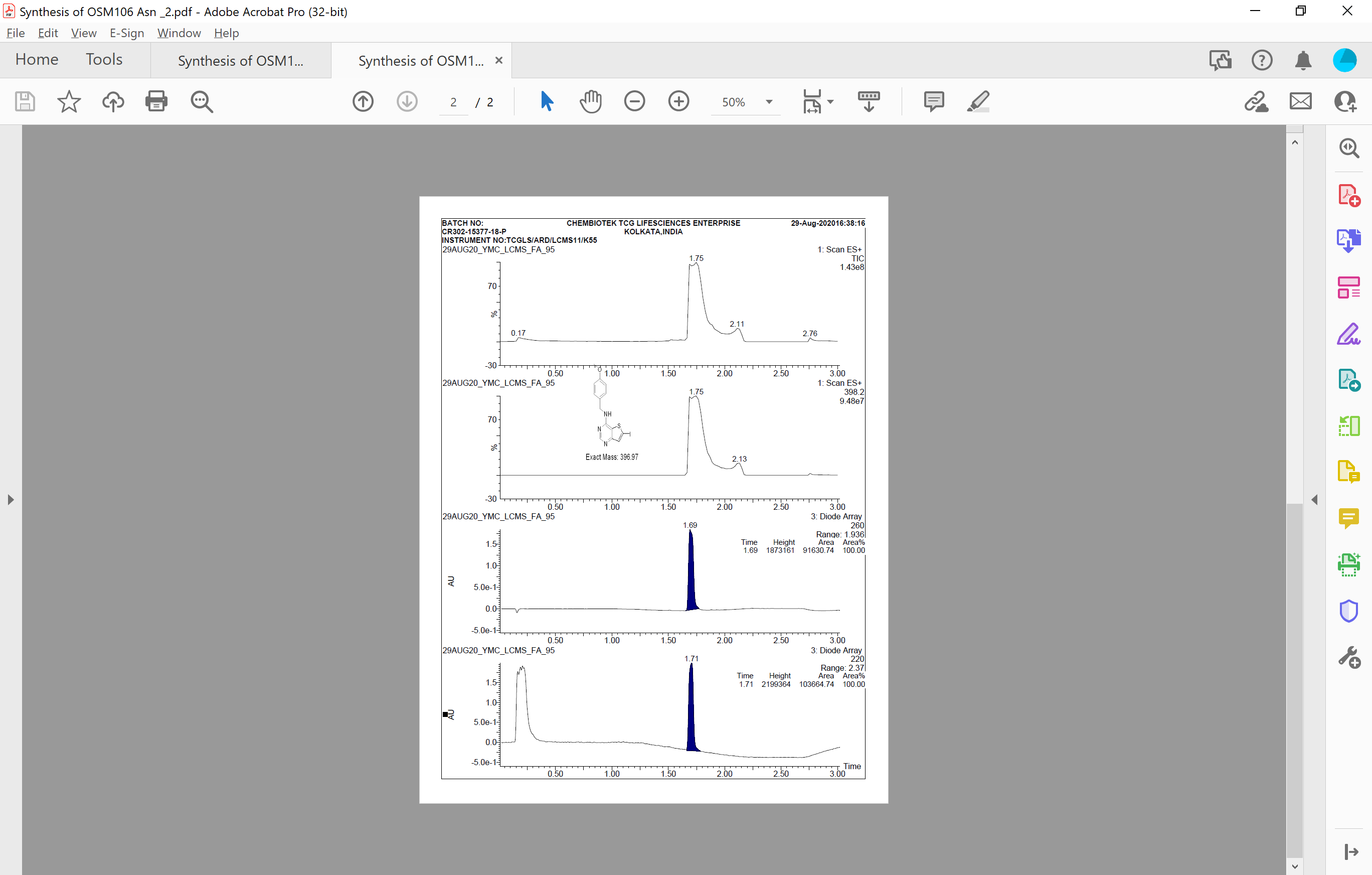

Supplement: Supplementary file 4 — Supplementary Dataset 1 [file 41467_2024_45224_MOESM4_ESM.docx]
