# Supplementary material for: Reaction hijacking inhibition of Plasmodium falciparum asparagine tRNA synthetase
Source: Nat Commun. 2024 Jan 31;15:937. doi: 10.1038/s41467-024-45224-z (PMC10831071; doi:10.1038/s41467-024-45224-z)
Supplement: Supplementary file 8 — Reporting Summary [file 41467_2024_45224_MOESM8_ESM.pdf]

nature portfolio

Corresponding author(s): Elizabeth A. Winzeler, Michael D. W. Griffin, Matthew H. Todd, Leann Tilley

Last updated by author(s): Jan 1, 2024

Reporting Summary

Nature Portfolio wishes to improve the reproducibility of the work that we publish. This form provides structure for consistency and transparency in reporting. For further information on Nature Portfolio policies, see our Editorial Policies and the Editorial Policy Checklist.

Statistics

For all statistical analyses, confirm that the following items are present in the figure legend, table legend, main text, or Methods section.

n/a	Confirmed	
☐	☒	The exact sample size (*n*) for each experimental group/condition, given as a discrete number and unit of measurement
☐	☒	A statement on whether measurements were taken from distinct samples or whether the same sample was measured repeatedly
☒	☐	The statistical test(s) used AND whether they are one- or two-sided<br>*Only common tests should be described solely by name; describe more complex techniques in the Methods section.*
☒	☐	A description of all covariates tested
☒	☐	A description of any assumptions or corrections, such as tests of normality and adjustment for multiple comparisons
☐	☒	A full description of the statistical parameters including central tendency (e.g. means) or other basic estimates (e.g. regression coefficient) AND variation (e.g. standard deviation) or associated estimates of uncertainty (e.g. confidence intervals)
☒	☐	For null hypothesis testing, the test statistic (e.g. *F*, *t*, *r*) with confidence intervals, effect sizes, degrees of freedom and *P* value noted<br>*Give P values as exact values whenever suitable.*
☒	☐	For Bayesian analysis, information on the choice of priors and Markov chain Monte Carlo settings
☒	☐	For hierarchical and complex designs, identification of the appropriate level for tests and full reporting of outcomes
☐	☒	Estimates of effect sizes (e.g. Cohen's *d*, Pearson's *r*), indicating how they were calculated

Our web collection on statistics for biologists contains articles on many of the points above.

Software and code

Policy information about availability of computer code

Data collection	BD FACSDiva (version 8.0); Clariostar Read Control (version 5.40 R3, BMG LABTECH); OptimaAUC (version 1.11)
Data analysis	FlowJo (verison 10.9); GraphPad Prism (version 9); Xcalibur (version 4.4); Skyline (version 21.1.0.278); SEDFIT (version 16.1c); MARS Data Analysis Software (version 3.32, BMG LABTECH); CCP4 software suite (verison 8.0); Phenix (version 1.19.2); COOT (version 0.9.8.1); ChimeraX (version 1.2.5); SYBYL-X 2.1; Pymol (version 2.5); Genome Analysis Toolkit (version 3.5); SnpEff (version 4.3).

For manuscripts utilizing custom algorithms or software that are central to the research but not yet described in published literature, software must be made available to editors and reviewers. We strongly encourage code deposition in a community repository (e.g. GitHub). See the Nature Portfolio guidelines for submitting code & software for further information.

Data

Policy information about availability of data

All manuscripts must include a data availability statement. This statement should provide the following information, where applicable:

- Accession codes, unique identifiers, or web links for publicly available datasets
- A description of any restrictions on data availability
- For clinical datasets or third party data, please ensure that the statement adheres to our policy

Additional data are available in Supplementary Information. Source data are provided. The following structures have been deposited in the PDB: HsAsnRS/Asn-AMP - PDB 8H53; HsAsnRS (apo) – PDB 8TC7; HsAsnRS/Asn-AMS – PDB 8TC8; HsAsnRS/Asn-OSM-S-106 – PDB 8TC9.

Research involving human participants, their data, or biological material

Policy information about studies with human participants or human data. See also policy information about sex, gender (identity/presentation), and sexual orientation and race, ethnicity and racism.

Reporting on sex and gender	*Use the terms sex (biological attribute) and gender (shaped by social and cultural circumstances) carefully in order to avoid confusing both terms. Indicate if findings apply to only one sex or gender; describe whether sex and gender were considered in study design; whether sex and/or gender was determined based on self-reporting or assigned and methods used. Provide in the source data disaggregated sex and gender data, where this information has been collected, and if consent has been obtained for sharing of individual-level data; provide overall numbers in this Reporting Summary. Please state if this information has not been collected. Report sex- and gender-based analyses where performed, justify reasons for lack of sex- and gender-based analysis.*
Reporting on race, ethnicity, or other socially relevant groupings	*Please specify the socially constructed or socially relevant categorization variable(s) used in your manuscript and explain why they were used. Please note that such variables should not be used as proxies for other socially constructed/relevant variables (for example, race or ethnicity should not be used as a proxy for socioeconomic status). Provide clear definitions of the relevant terms used, how they were provided (by the participants/respondents, the researchers, or third parties), and the method(s) used to classify people into the different categories (e.g. self-report, census or administrative data, social media data, etc.) Please provide details about how you controlled for confounding variables in your analyses.*
Population characteristics	*Describe the covariate-relevant population characteristics of the human research participants (e.g. age, genotypic information, past and current diagnosis and treatment categories). If you filled out the behavioural & social sciences study design questions and have nothing to add here, write "See above."*
Recruitment	*Describe how participants were recruited. Outline any potential self-selection bias or other biases that may be present and how these are likely to impact results.*
Ethics oversight	*Identify the organization(s) that approved the study protocol.*

Note that full information on the approval of the study protocol must also be provided in the manuscript.

Field-specific reporting

Please select the one below that is the best fit for your research. If you are not sure, read the appropriate sections before making your selection.

☒ Life sciences ☐ Behavioural & social sciences ☐ Ecological, evolutionary & environmental sciences

For a reference copy of the document with all sections, see nature.com/documents/nr-reporting-summary-flat.pdf

Life sciences study design

All studies must disclose on these points even when the disclosure is negative.

Sample size	For flow cytometry based cellular assays, infected red blood cells were identified and parasitemia was calculated from 30,000 red blood cells for each condition. The number of experimental replicates is provided for all experiments. N = 3 or more was chosen to allow calculation of Standard Error.
Data exclusions	No data was excluded.
Replication	All biochemical experiments were repeated independently for at least three times, each repeat was performed with technical duplicates.<br>All cellular experiments were repeated independently for at least two times, each repeat was performed with technical duplicates.<br>All attempts at replication were successful and results are comparable from each experiment.
Randomization	No randomisation was used in this study as there are no known input co-variants. All biochemical analysis involved sample prepared in the same way.
Blinding	No blinding was used in this study. The biochemical samples were all prepared in the same way and replicability of the experiments was high. The data points represent instrument readouts which are not susceptible to experimenter bias.

Reporting for specific materials, systems and methods

We require information from authors about some types of materials, experimental systems and methods used in many studies. Here, indicate whether each material, system or method listed is relevant to your study. If you are not sure if a list item applies to your research, read the appropriate section before selecting a response.

Materials & experimental systems

n/a	Involved in the study
[ ]	[x] Antibodies
[ ]	[x] Eukaryotic cell lines
[x]	[ ] Palaeontology and archaeology
[x]	[ ] Animals and other organisms
[x]	[ ] Clinical data
[x]	[ ] Dual use research of concern
[x]	[ ] Plants

Methods

n/a	Involved in the study
[x]	[ ] ChIP-seq
[ ]	[x] Flow cytometry
[x]	[ ] MRI-based neuroimaging

Antibodies

Antibodies used

Primary antibodies: rabbit anti-phospho-eIF2α (Cell Signaling Technology-119A11; Lot 12 Ref no. 3597L; 1:1000); polyclonal mouse anti-PfBiP (WEHI; 1:1000).
Secondary antibodies: goat anti-rabbit IgG-HRP (Chemicon-AP132P; Lot 3584340; 1:20,000); goat anti-mouse IgG-HRP (Chemicon-AP181P; Lot 3557957; 1:50,000).

Validation

Antibodies were used to detect phosphorylated eIF2α and PfBiP in this study. Protein bands of the correct molecular mass were detected, with minimal background and non specific labeling. The antibodies were validated in Xie SC, et al, Reaction hijacking of tyrosine tRNA synthetase as a new whole-of-life-cycle antimalarial strategy. Science 376, 1074-1079 (2022).
All secondary antibodies have been validated for specificity and cross reactivity by the manufacturer.

Eukaryotic cell lines

Policy information about cell lines and Sex and Gender in Research

Cell line source(s)

P.falciparum 3D7, Dd2 and Cam3.IIRev strains (BEI Resources; Xie SC, et al, Reaction hijacking of tyrosine tRNA synthetase as a new whole-of-life-cycle antimalarial strategy. Science. 376, 1074-1079 (2022); K13-propeller mutations confer artemisinin resistance in Plasmodium falciparum clinical isolates. Science. 347, 428-31 (2015)).
PfAsnRSR487S transfectant cell line was generated based on P.falciparum Dd2 strain (CRISPR/Cas9 editing of the Plasmodium falciparum genome. Methods Mol Biol. 2470, 221-239 (2022)).
Conditional knockdown (cKD) P. falciparum lines were generated based on NF54 parasites (Synthetic RNA–protein modules integrated with native translation mechanisms to control gene expression in malaria parasites. Nature Communications. 7, 10727(2016)).
P.berghei and hepatic human transformed cell line with GFP-CD81 fusion (HepG2-A16-CD81-GFP) (Swann J, et al, High-throughput luciferase-based assay for the discovery of therapeutics that prevent malaria. ACS infectious diseases. 2, 281-293 (2016)).

Authentication

Transfectants validated using PCR and sequencing

Mycoplasma contamination

Cultures tested and shown to be mycoplasma-free.

Commonly misidentified lines
(See ICLAC register)

No commonly misidentified lines were used.

Flow Cytometry

Plots

Confirm that:

- [ ] The axis labels state the marker and fluorochrome used (e.g. CD4-FITC).
- [ ] The axis scales are clearly visible. Include numbers along axes only for bottom left plot of group (a 'group' is an analysis of identical markers).
- [ ] All plots are contour plots with outliers or pseudocolor plots.
- [x] A numerical value for number of cells or percentage (with statistics) is provided.

Methodology

Sample preparation

Live infected RBCs were labelled with nucleic acid stain 2 uM Syto-61 or 25 µg/ml propidium iodide

Instrument

FACSCanto™ II cytometer; Becton Dickinson

Software

FlowJo

Cell population abundance

Uninfected red blood cells (no nucleic acid) give background signal. Infected red blood cells are separated based on uptake of

the nucleic acid stain.

Gating strategy

The gating strategy was described previously in Fu, Y., L. Tilley, S. Kenny, and N. Klonis. 2010. Dual labeling with a far red probe permits analysis of growth and oxidative stress in P. falciparum-infected erythrocytes. Cytometry A. 77:253-263.

☒ Tick this box to confirm that a figure exemplifying the gating strategy is provided in the Supplementary Information.
